# Correction: Mechanisms of synergy creation for social-ecological transformation: Leverage point analysis of the emergence of autonomous innovations

**DOI:** 10.1371/journal.pone.0337698

**Published:** 2025-11-25

**Authors:** Hidetomo Tajima, Tetsu Sato, John Banana Matewere, Dorothea Agnes Rampisela, Motoko Shimagami, Shion Takemura, Mitsutaku Makino

The references 9, 20, 24, 27, 28, 40, 67, and 70 are incorrect. The correct references are:

9 Sato T, Chabay I, Helgeson J. Conclusion and Way Forward. In: Sato T, Chabay I, Helgeson J, editors. Transformations of Social-Ecological Systems: Studies in Co-Creating Integrated Knowledge Toward Sustainable Futures. Springer Singapore; 2018. pp. 409−16. https://doi.org/10.1007/978-981-13-2327-0_22
20 Pryshlakivsky J, Cory S. Sustainable development as a wicked problem. In: Kovacic SF, Sousa-Poza A, editors. Managing and Engineering in Complex Situations. Topics in Safety, Risk, Reliability and Quality Volume 21. Dordrecht: Springer Nature; 2012. pp. 109−28. https://doi.org/10.1007/978-94-007-5515-4_6
24 Makino M, Hori M, Nanami A, Hori J, Tajima, H. Mapping the Policy Interventions on Marine Social-Ecological Systems: Case Study of Sekisei Lagoon, Southwest Japan. In: Saito O, Subramanian S, Hashimoto S, Takeuchi K. editors. Managing Socio-ecological Production Landscapes and Seascapes for Sustainable Communities in Asia. Science for Sustainable Societies. Springer Singapore; 2020. pp. 11−30. https://doi.org/10.1007/978-981-15-1133-2_2
27 Sato T, Pemba D, Nakagawa C. Innovation Emerging from Livelihoods: Natural Resource Management in Lake Malawi. In: Sato T, Chabay I, Helgeson J, editors. Transformations of Social-Ecological Systems: Studies in Co-Creating Integrated Knowledge Toward Sustainable Futures. Springer Singapore; 2018. pp. 137–55. https://doi.org/10.1007/978-981-13-2327-0_8
28 Sato T, Pemba D. Villagers managing lake fisheries resources by themselves: Mbenji Islands in Lake Malawi. In; Kakuma S, Yanagi T, Sato T, editors. Satoumi Science: Co-Creating Social-Ecological Harmony Between Human and the Sea, Springer Singapore; 2022. pp. 145−67. https://doi.org/10.1007/978-981-16-7491-4_9
40 Carmichael T, Hadžikadić M. The Fundamentals of Complex Adaptive Systems. In; Carmichael T, Collins A, Hadžikadić M, editors. Complex Adaptive Systems. Understanding Complex Systems, Springer, Cham; 2019. pp. 1−19. https://doi.org/10.1007/978-3-030-20309-2_1
67 Helgeson J. Institutional support for combining multiple knowledge systems in planning for community resilience to natural and anthropogenic hazards. In: Sato T, Chabay I, Helgeson J, editors. Transformations of Social-Ecological Systems: Studies in Co-Creating Integrated Knowledge toward Sustainable Futures. Springer Singapore; 2018. pp. 373−91. https://doi.org/10.1007/978-981-13-2327-0_20
70 White DD, Larson KL, Wutich A. Boundary Organizations and Objects Supporting Stakeholders for Decision Making on Sustainable Water Management in Phoenix, Arizona USA. In: Sato T, Chabay I, Helgeson J, editors. Transformations of Social-Ecological Systems: Studies in Co-Creating Integrated Knowledge Toward Sustainable Futures. Springer Singapore. 2018. pp. 333−52. https://doi.org/10.1007/978-981-13-2327-0_18
