# Supplementary material for: Mechanisms of synergy creation for social-ecological transformation: Leverage point analysis of the emergence of autonomous innovations
Source: PLoS One. 2025 May 14;20(5):e0323451. doi: 10.1371/journal.pone.0323451 (PMC12077674; doi:10.1371/journal.pone.0323451)

No.1：Community-based marine tourism

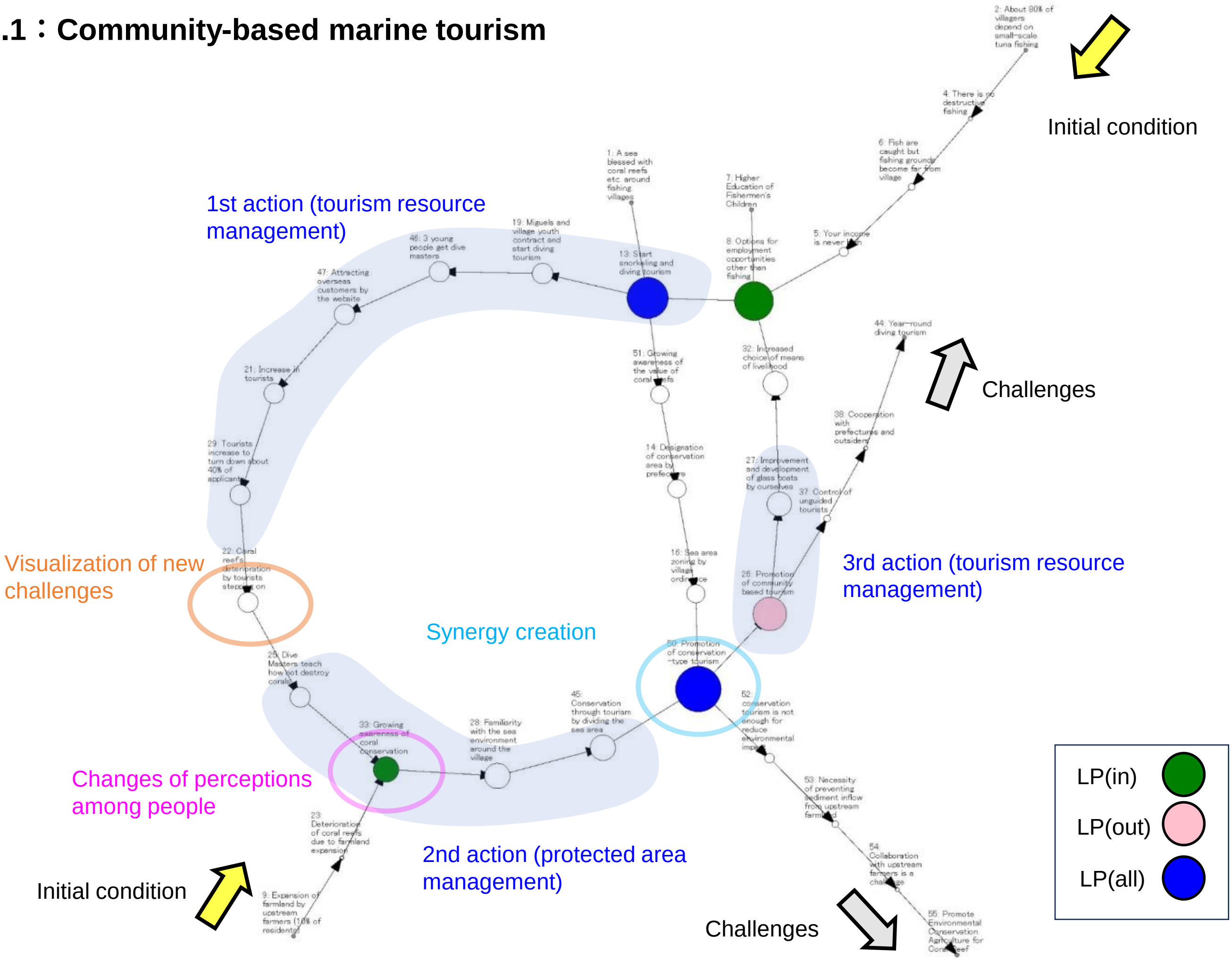

No.2：Improving the quality of cacao raw materials and high value-added distribution

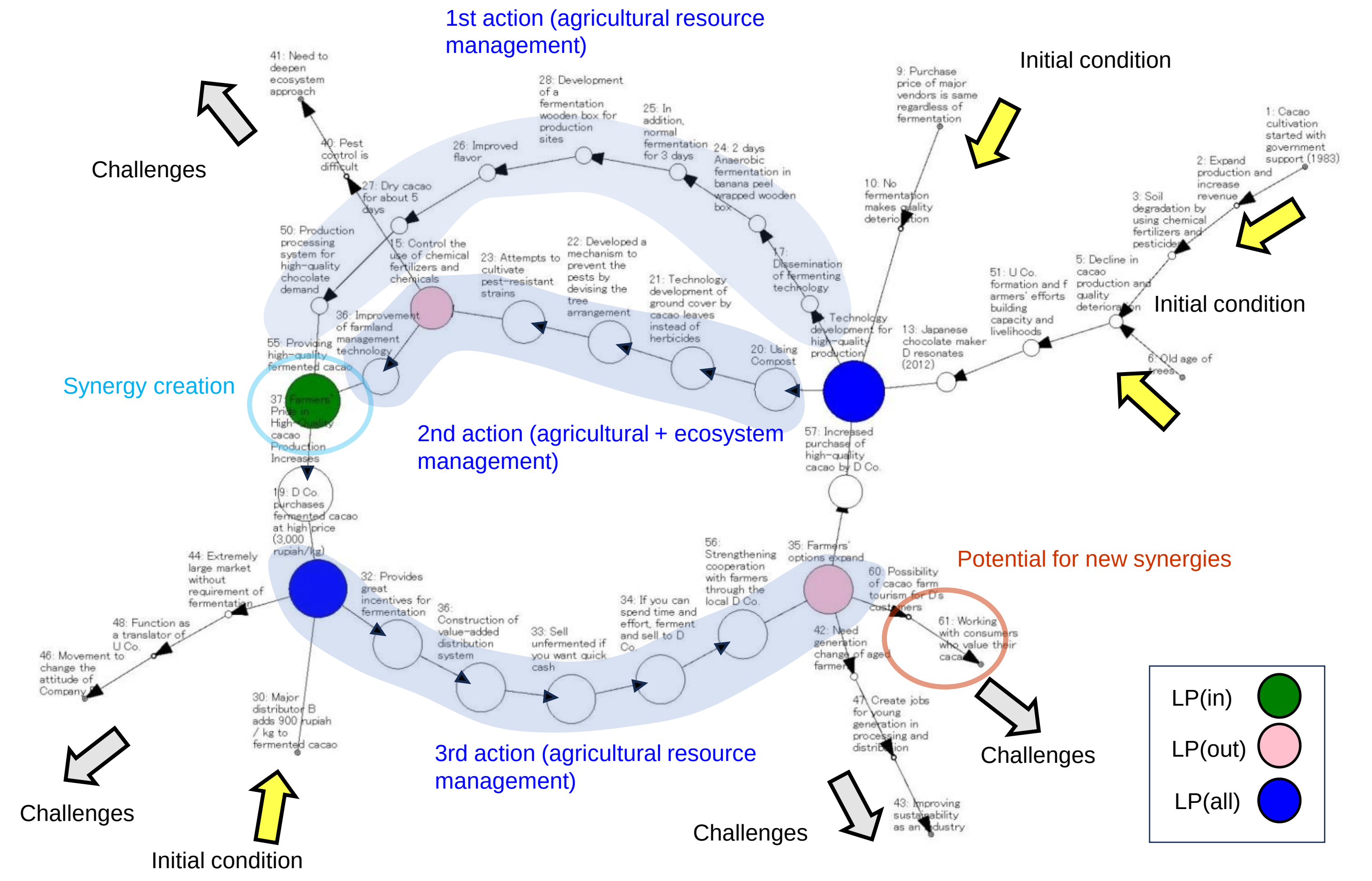

No.3：Improving cacao farm management

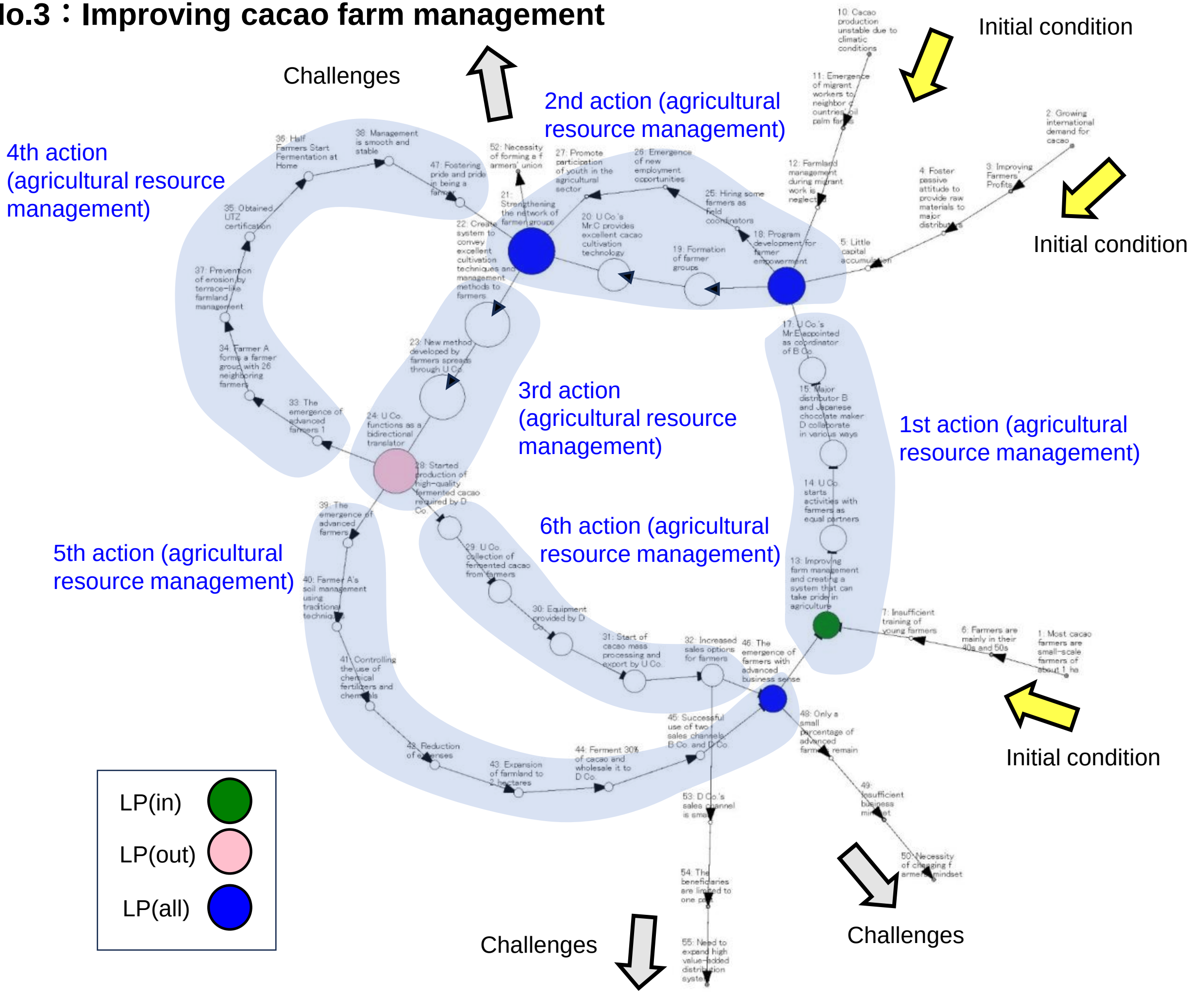

No.4：Multi-species cultivation on cacao farmland

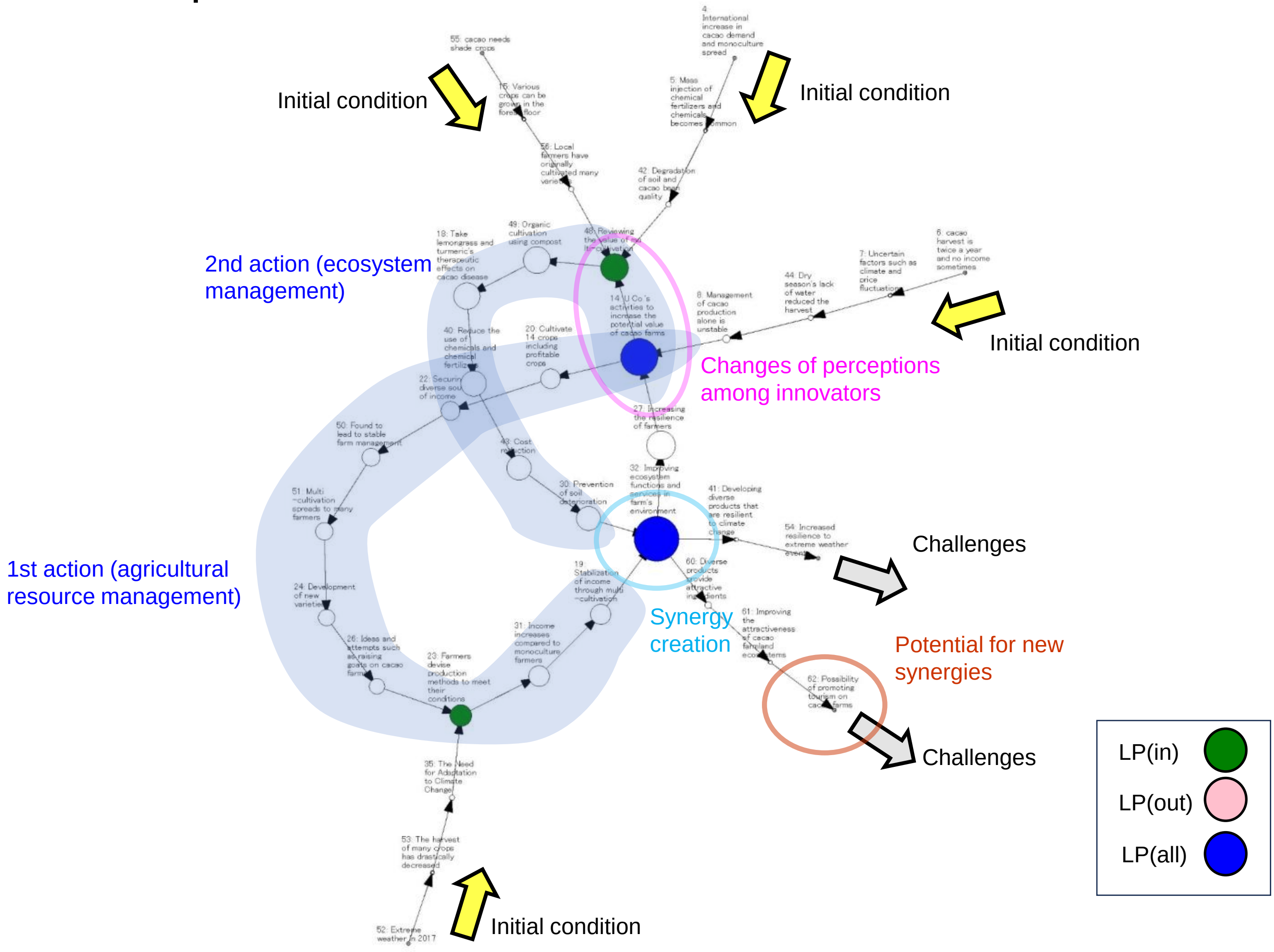

No.5：Development of cacao farm tourism

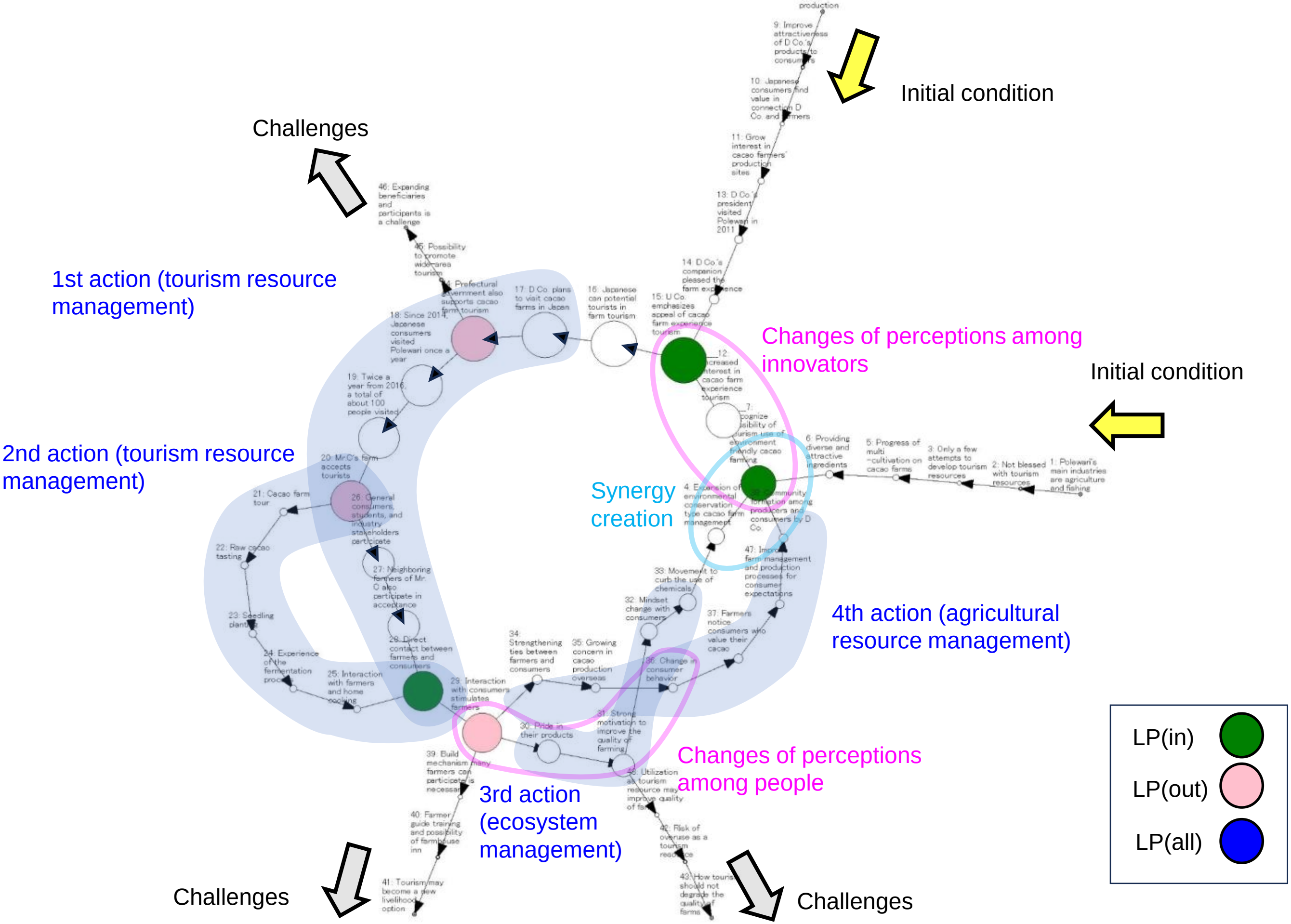

No.6：Collaborative network construction

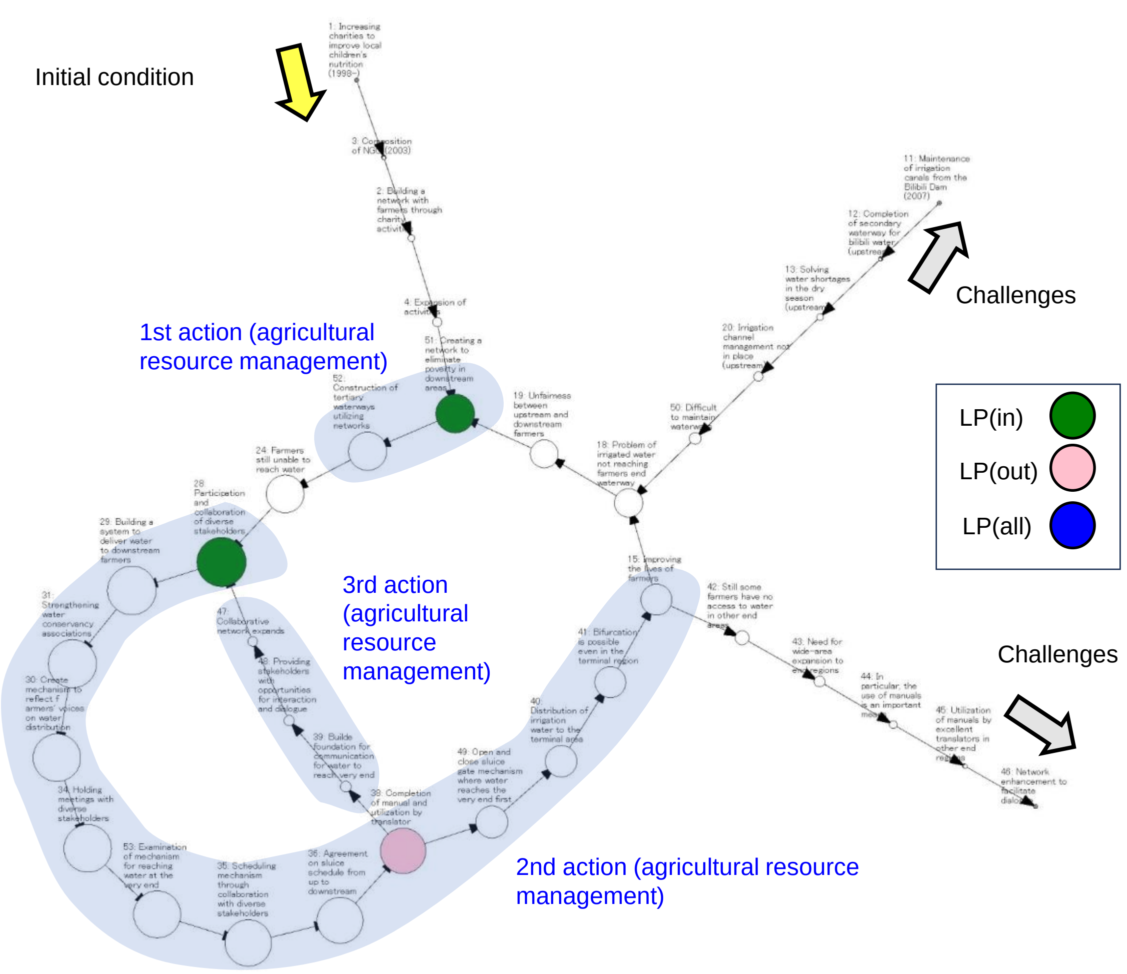

No.7：Waste recycling and Tourism development

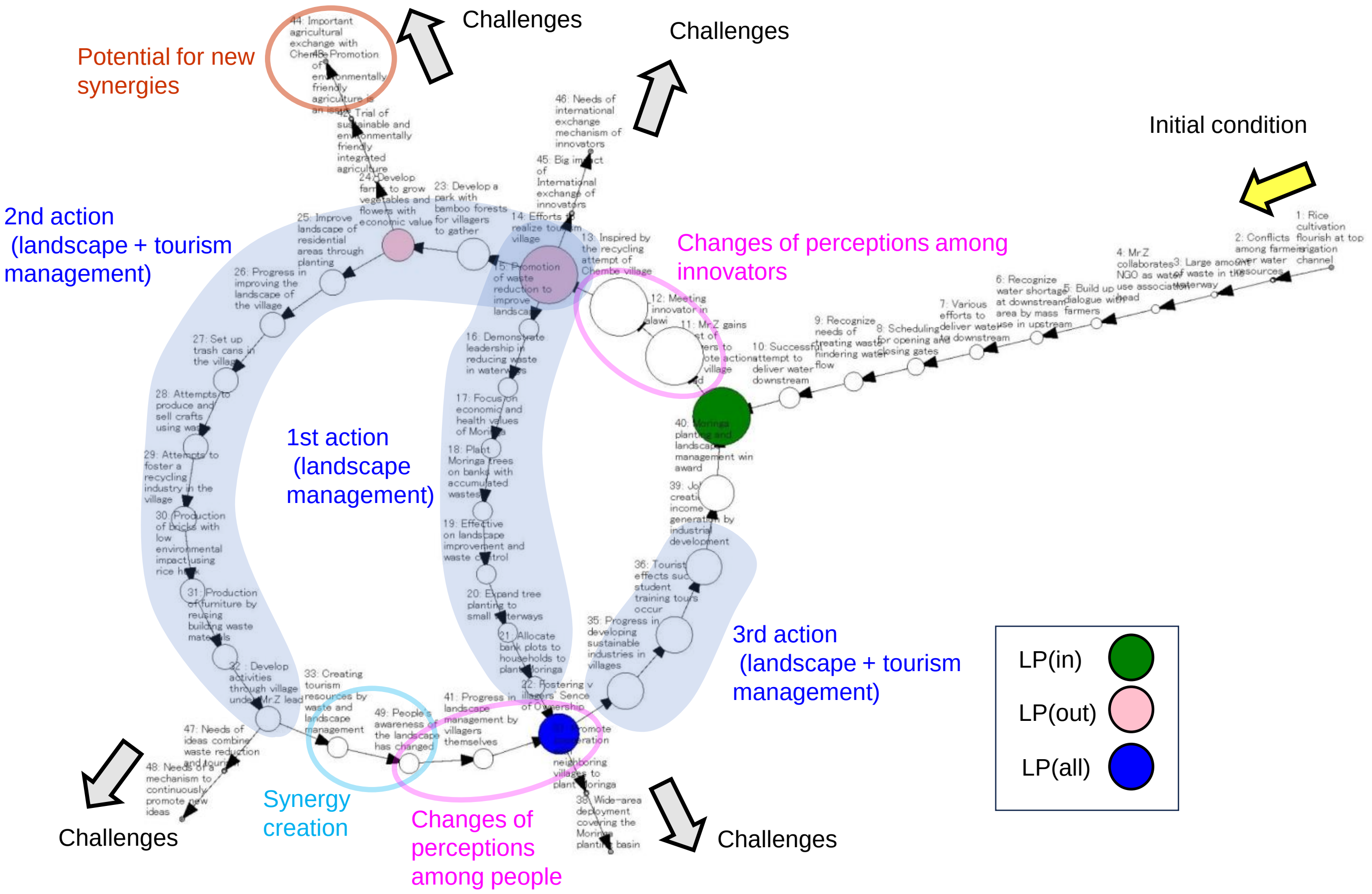

No.8：Improvement of rice planting method through international exchange

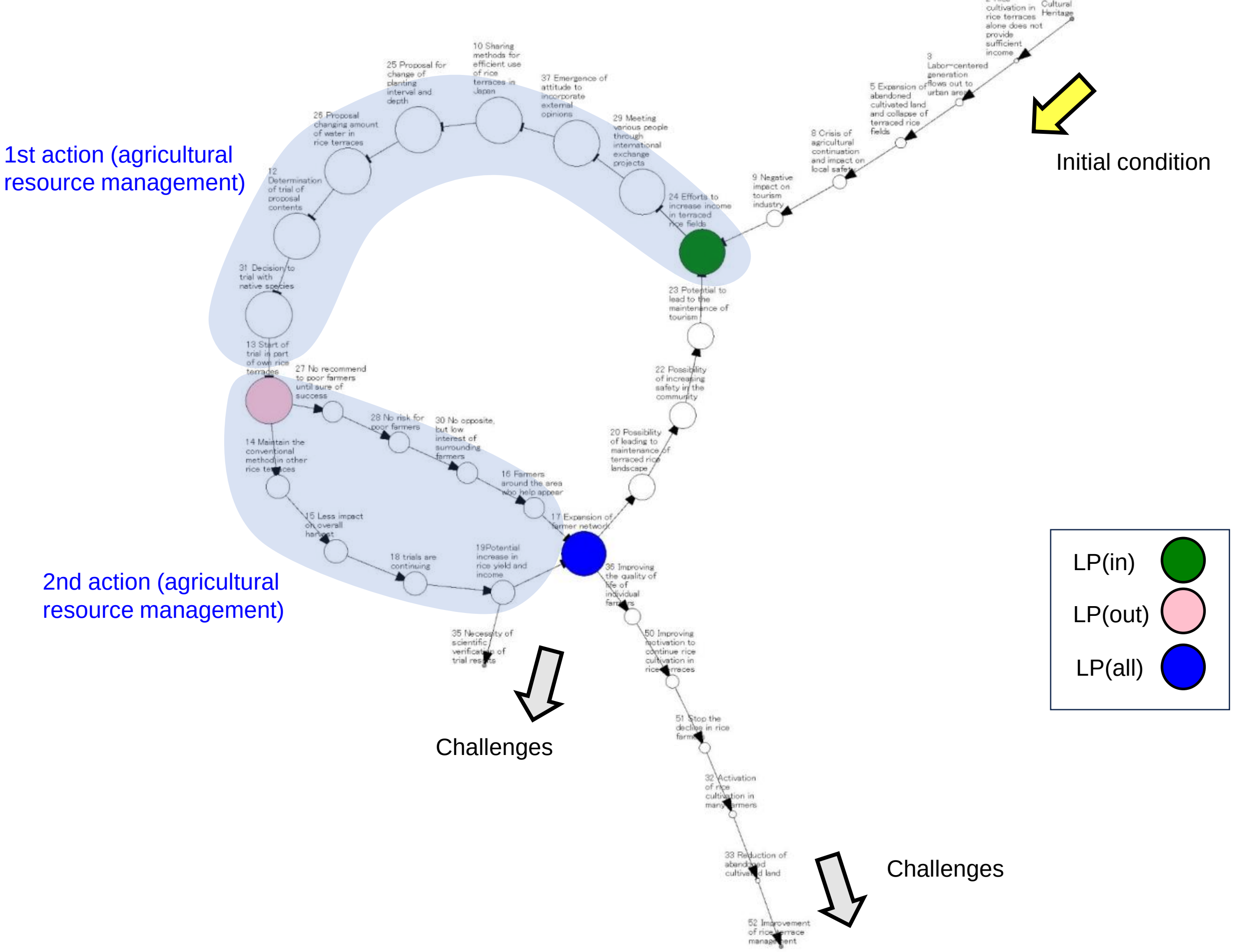

No.9：Diversification of production activities of natural rubber plantations

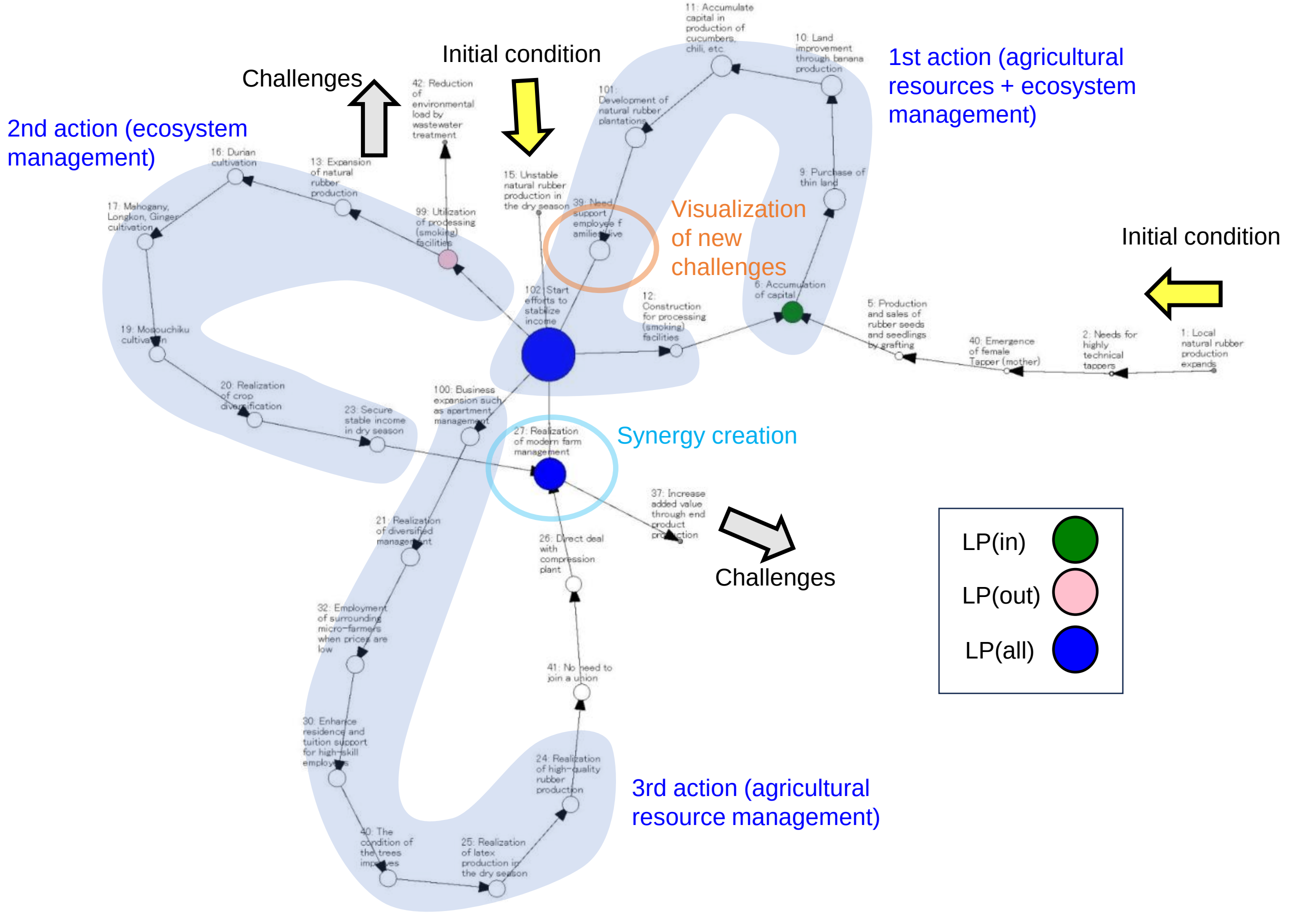

No.10：Reorganization and utilization of traditional salt making techniques

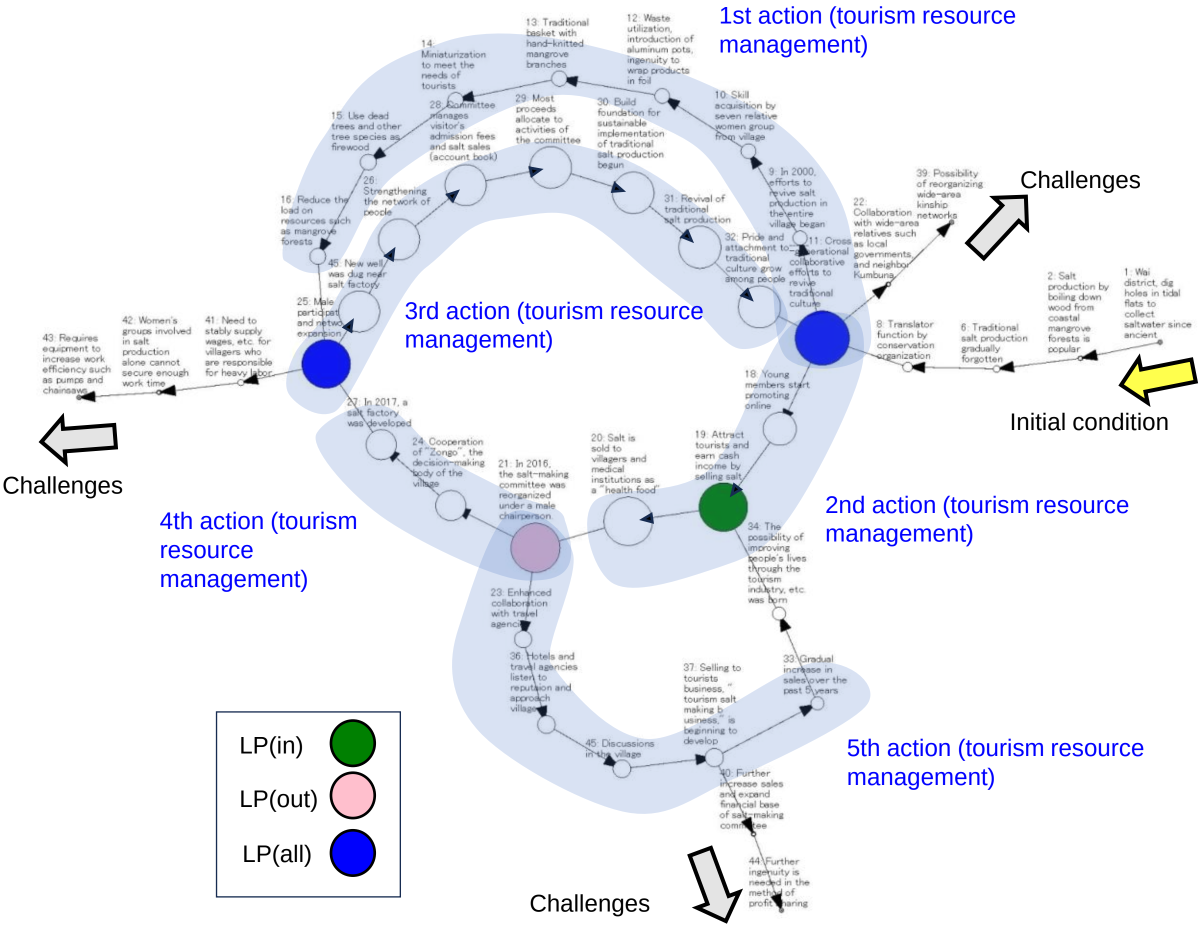

No.11：Small-scale aquaculture and multi-species cultivation

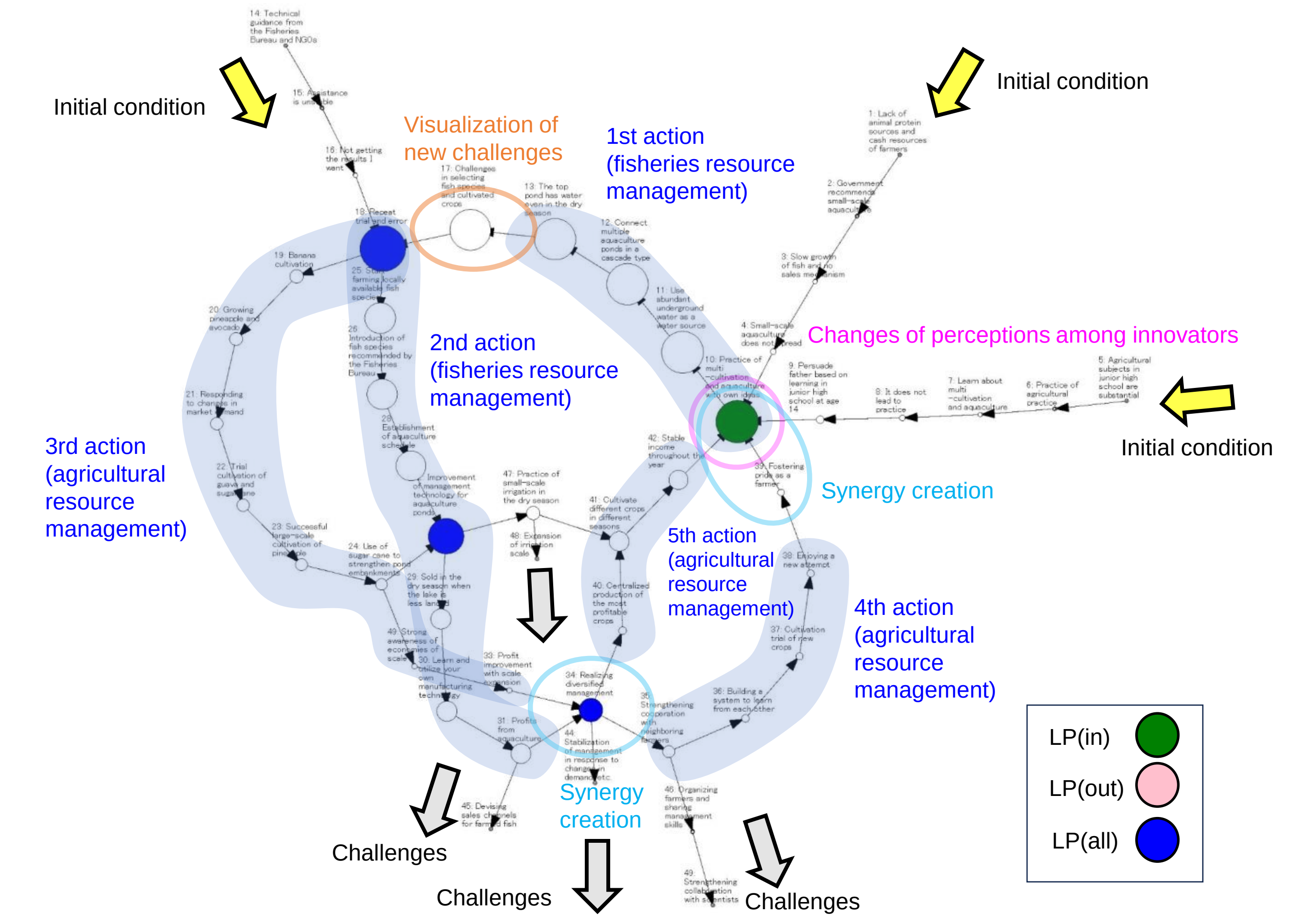

No.12：Seasonal fishing bans around Mbenji Island by traditional chiefs and communities

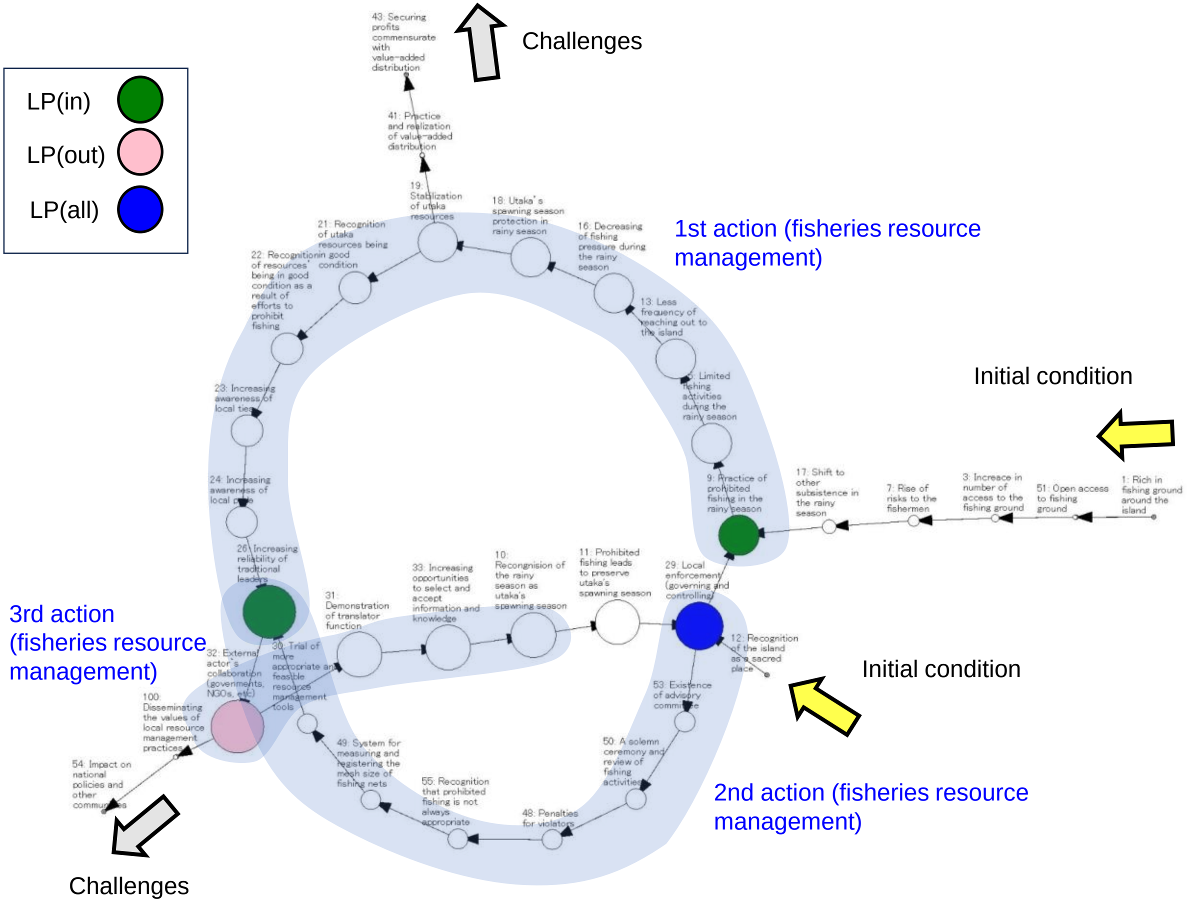

No.13：Formation and operation of a tour guide association by local residents

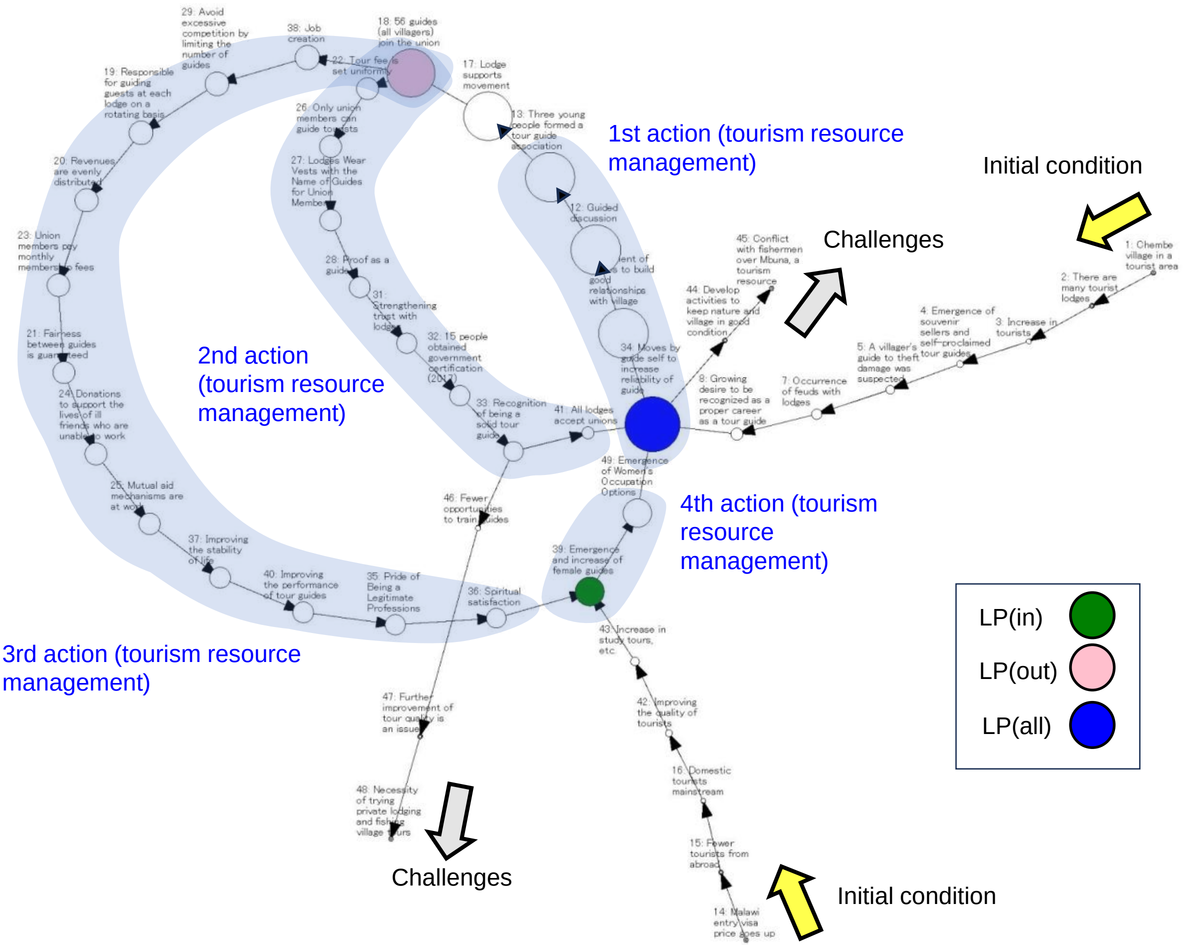

No.14：Cape Maclear Cleanup project and recycling center

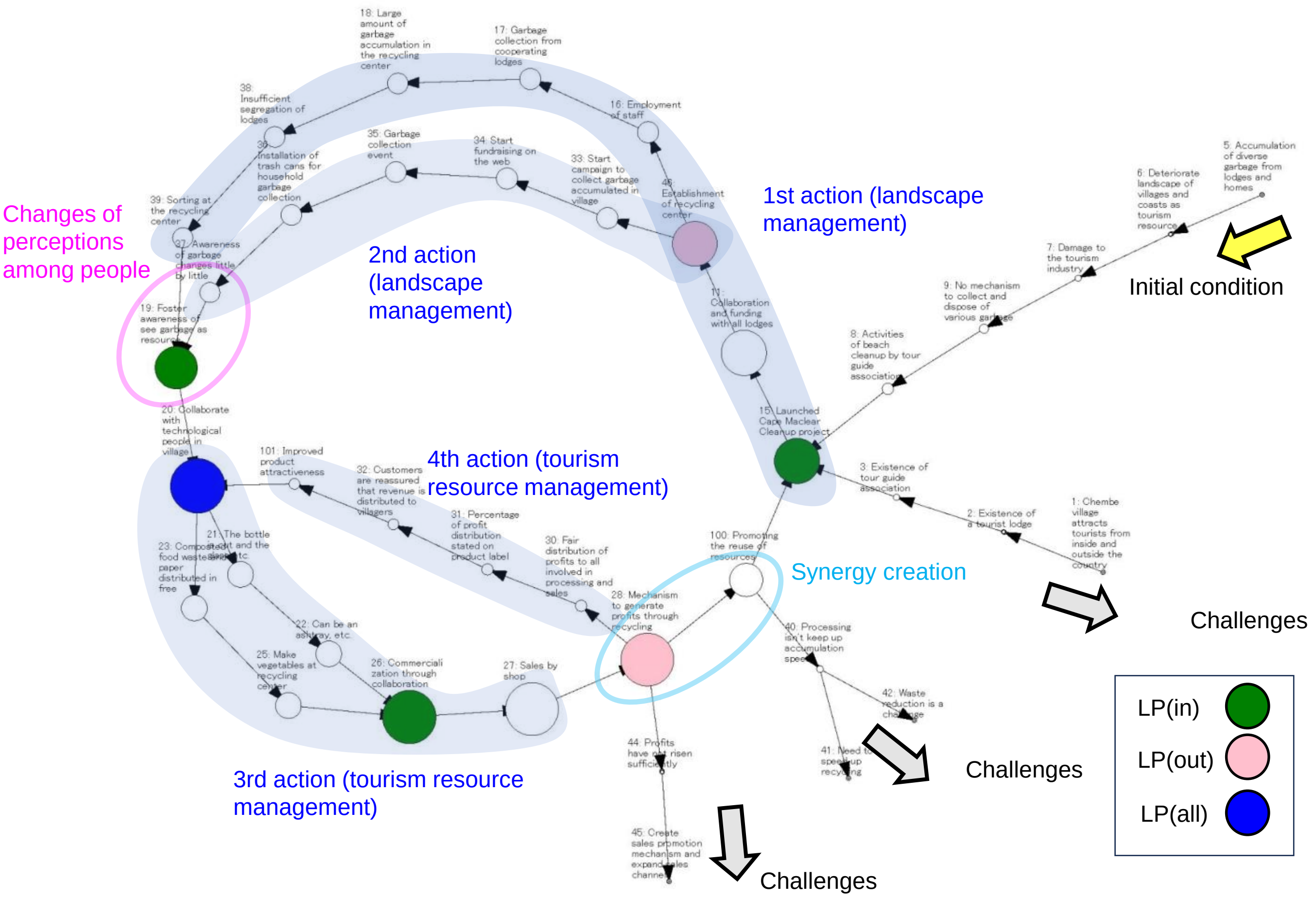

No.15：Organic farming by small-scale irrigation linked to educational activities

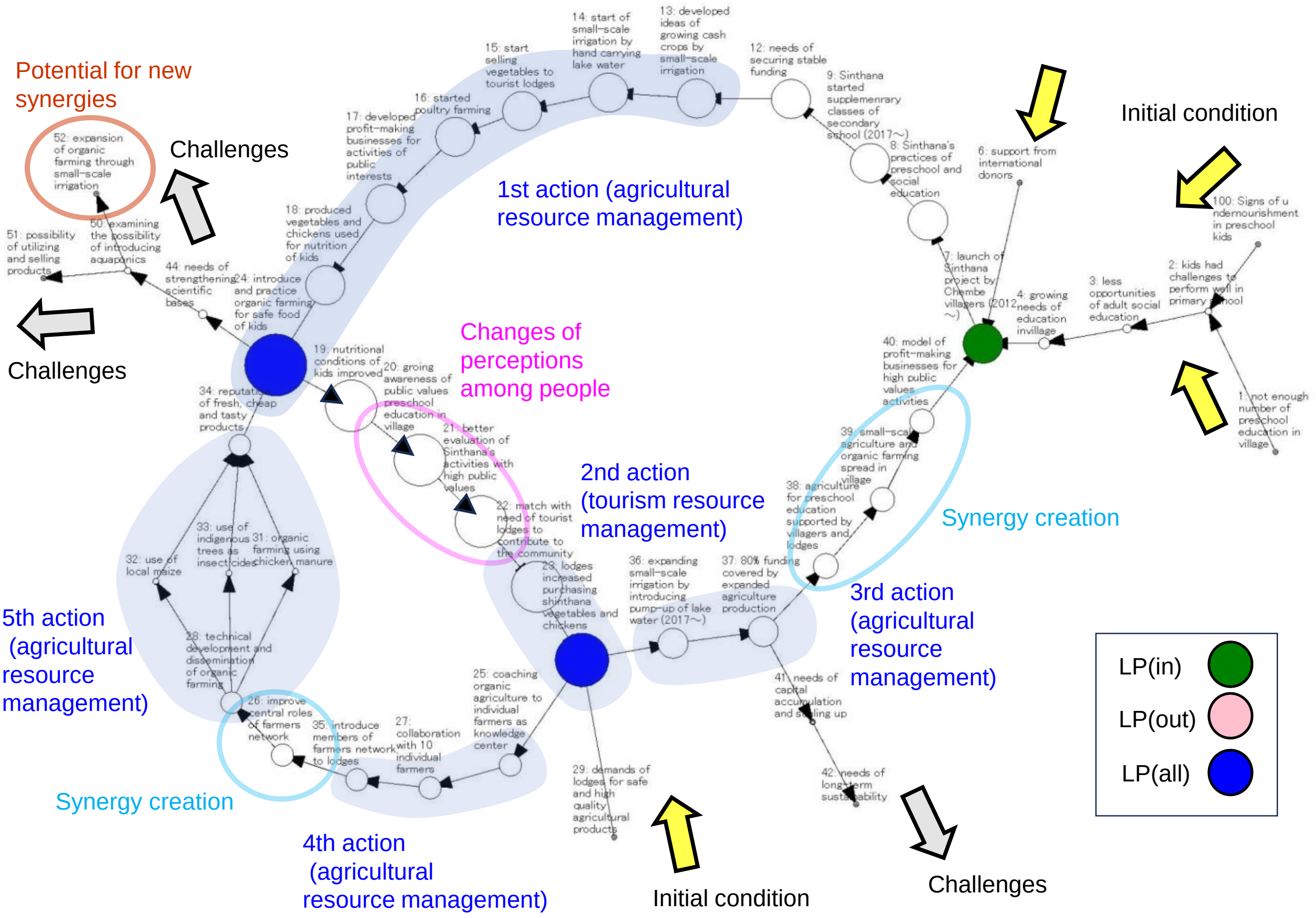

No.16：Efforts by fishers to create satoumi-type fishing grounds

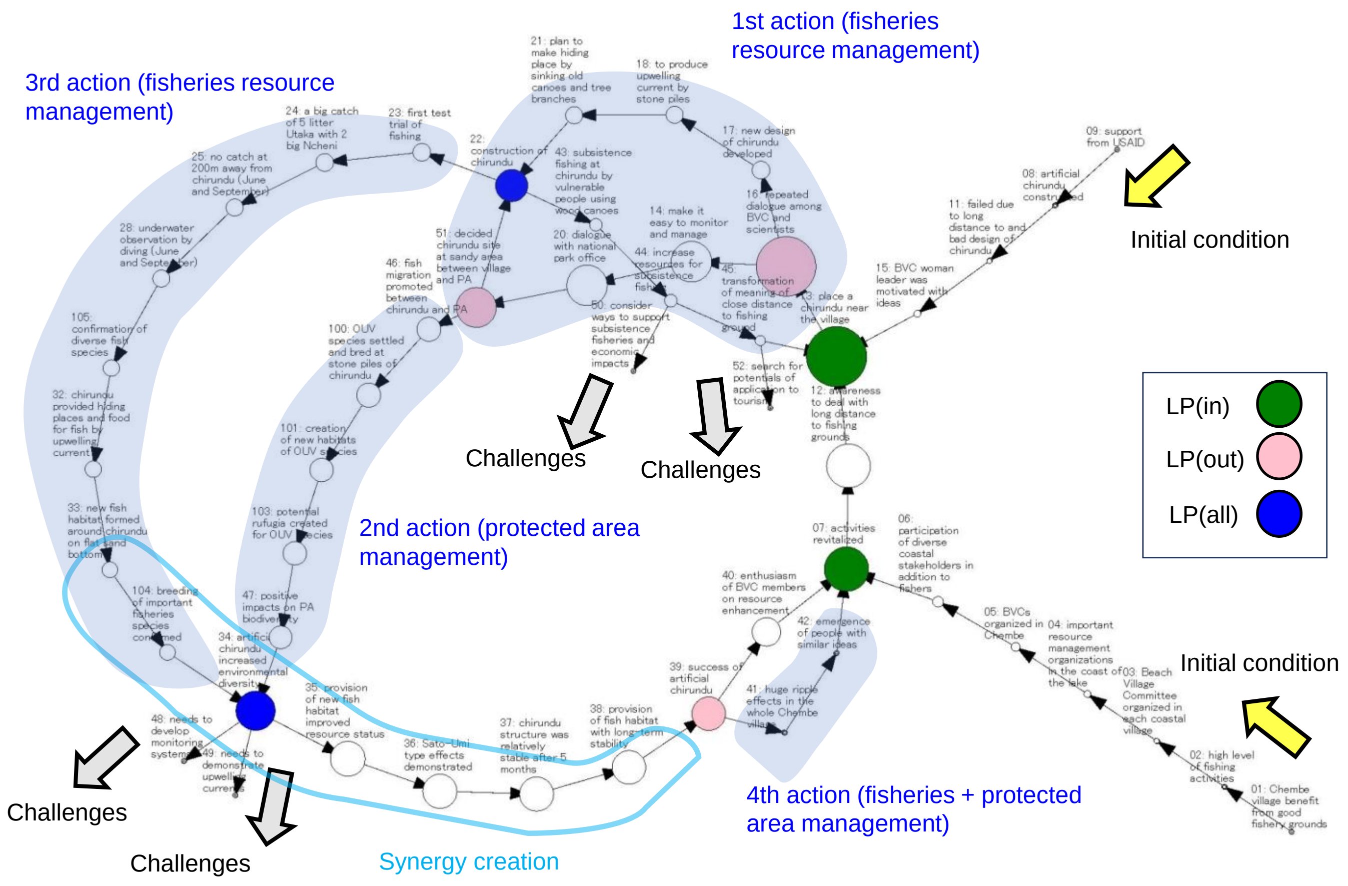

No.17：Cultivation and sale of pickled salad melons requiring small amount of irrigation water

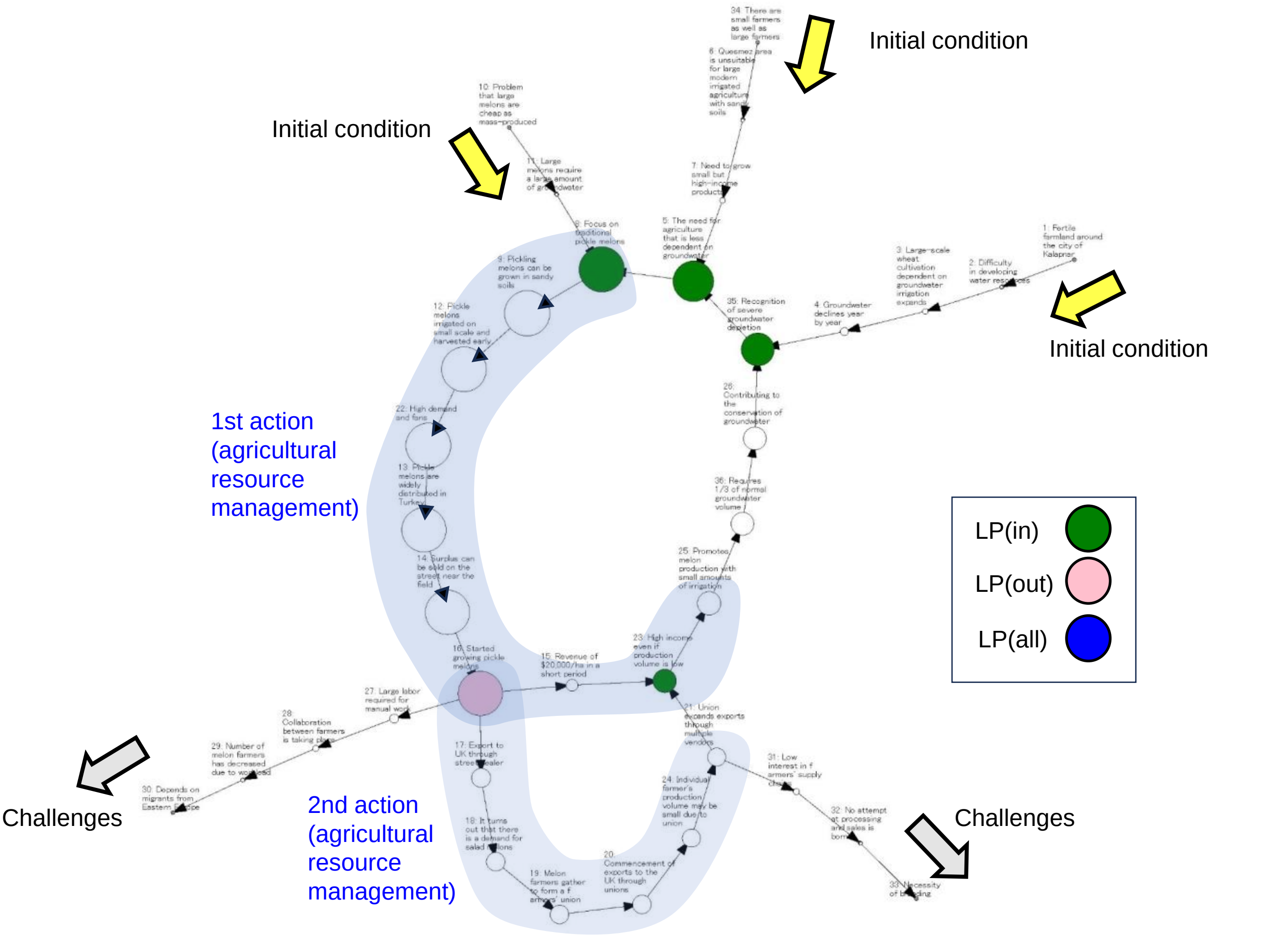

Supplement: S1 — Initial conditions, collective actions, remaining challenges, leverage points, timing of synergy creation, factors of synergy creation, and potential for new synergies are presented for each autonomous innovation. These diagrams were used for the analysis to develop Fig 5. (PDF) [file pone.0323451.s001.pdf]
