# Supplementary material for: Mechanisms of synergy creation for social-ecological transformation: Leverage point analysis of the emergence of autonomous innovations
Source: PLoS One. 2025 May 14;20(5):e0323451. doi: 10.1371/journal.pone.0323451 (PMC12077674; doi:10.1371/journal.pone.0323451)
Supplement: S2 — The nodes and links for causal network analyses are represented for each autonomous innovation. (PDF) [file pone.0323451.s002.pdf]

No.1 Community-based marine tourism

link_id	from_id	from_label	to_id	to_label
1	2	2: About 80% of villagers depend on small-scale tuna fishing	4	4: There is no destructive fishing
2	4	4: There is no destructive fishing	6	6: Fish are caught but fishing grounds become far from village
3	6	6: Fish are caught but fishing grounds become far from village	5	5: Your income is never high
4	1	1: A sea blessed with coral reefs etc. around fishing villages	13	13: Start snorkeling and diving tourism
5	8	8: Options for employment opportunities other than fishing	13	13: Start snorkeling and diving tourism
6	5	5: Your income is never high	8	8: Options for employment opportunities other than fishing
7	7	7: Higher Education of Fishermen's Children	8	8: Options for employment opportunities other than fishing
8	9	9: Expansion of farmland by upstream farmers (10% of residents)	23	23: Deterioration of coral reefs due to farmland expansion
9	23	23: Deterioration of coral reefs due to farmland expansion	33	33: Growing awareness of coral conservation
10	26	26: Promotion of community based tourism	27	27: Improvement and development of glass boats by ourselves
11	19	19: Miguels and village youth contract and start diving tourism	46	46: 3 young people get dive masters
12	46	46: 3 young people get dive masters	47	47: Attracting overseas customers by the website
13	47	47: Attracting overseas customers by the website	21	21: Increase in tourists
14	14	14: Designation of conservation area by prefecture	16	16: Sea area zoning by village ordinance
15	21	21: Increase in tourists	29	29: Tourists increase to turn down about 40% of applicants
16	22	22: Coral reef's deterioration by tourists stepping on	25	25: Dive Masters teach how not destroy corals
17	33	33: Growing awareness of coral conservation	28	28: Familiarity with the sea environment around the village
18	26	26: Promotion of community based tourism	37	37: Control of unguided tourists
19	37	37: Control of unguided tourists	38	38: Cooperation with prefectures and outsiders
20	28	28: Familiarity with the sea environment around the village	45	45: Conservation through tourism by dividing the sea area
21	27	27: Improvement and development of glass boats by ourselves	32	32: Increased choice of means of livelihood
22	38	38: Cooperation with prefectures and outsiders	44	44: Year-round diving tourism
23	32	32: Increased choice of means of livelihood	8	8: Options for employment opportunities other than fishing
24	25	25: Dive Masters teach how not destroy corals	33	33: Growing awareness of coral conservation
25	16	16: Sea area zoning by village ordinance	50	50: Promotion of conservation-type tourism
26	50	50: Promotion of conservation-type tourism	26	26: Promotion of community based tourism
27	29	29: Tourists increase to turn down about 40% of applicants	22	22: Coral reef's deterioration by tourists stepping on
28	45	45: Conservation through tourism by dividing the sea area	50	50: Promotion of conservation-type tourism
29	13	13: Start snorkeling and diving tourism	19	19: Miguels and village youth contract and start diving tourism
30	13	13: Start snorkeling and diving tourism	51	51: Growing awareness of the value of coral reefs
31	51	51: Growing awareness of the value of coral reefs	14	14: Designation of conservation area by prefecture
32	50	50: Promotion of conservation-type tourism	52	52: conservation tourism is not enough for reduce environmental impact
33	52	52: conservation tourism is not enough for reduce environmental impact	53	53: Necessity of preventing sediment inflow from upstream farmland
34	53	53: Necessity of preventing sediment inflow from upstream farmland	54	54: Collaboration with upstream farmers is a challenge
35	54	54: Collaboration with upstream farmers is a challenge	55	55: Promote Environmental Conservation Agriculture for Coral Reef

No.2 Improving the quality of cacao raw materials and high value-added distribution

link_id	from_id	from_label	to_id	to_label
1	1	1: Cacao cultivation started with government support (1983)	2	2: Expand production and increase revenue
2	2	2: Expand production and increase revenue	3	3: Soil degradation by using chemical fertilizers and pesticides
3	3	3: Soil degradation by using chemical fertilizers and pesticides	5	5: Decline in cacao production and quality deterioration
4	6	6: Old age of trees	5	5: Decline in cacao production and quality deterioration
5	5	5: Decline in cacao production and quality deterioration	51	51: U Co. formation and farmers' efforts building capacity and livelihoods
6	51	51: U Co. formation and farmers' efforts building capacity and livelihoods	13	13: Japanese chocolate maker D resonates (2012)
7	13	13: Japanese chocolate maker D resonates (2012)	16	16: Technology development for high-quality production
8	9	9: Purchase price of major vendors is same regardless of fermentation	10	10: No fermentation makes quality deterioration
9	10	10: No fermentation makes quality deterioration	16	16: Technology development for high-quality production
10	30	30: Major distributor B adds 900 rupiah / kg to fermented cacao	19	19: D Co. purchases fermented cacao at high price (3,000 rupiah/kg)
11	16	16: Technology development for high-quality production	17	17: Dissemination of fermenting technology
12	17	17: Dissemination of fermenting technology	24	24: 2 days Anaerobic fermentation in banana peel wrapped wooden box
13	24	24: 2 days Anaerobic fermentation in banana peel wrapped wooden box	25	25: In addition, normal fermentation for 3 days
14	25	25: In addition, normal fermentation for 3 days	28	28: Development of a fermentation wooden box for production sites
15	28	28: Development of a fermentation wooden box for production sites	26	26: Improved flavor
16	26	26: Improved flavor	27	27: Dry cacao for about 5 days
17	27	27: Dry cacao for about 5 days	50	50: Production processing system for high-quality chocolate demand
18	50	50: Production processing system for high-quality chocolate demand	55	55: Providing high-quality fermented cacao
19	55	55: Providing high-quality fermented cacao	37	37: Farmers’ Pride in High-Quality cacao Production Increases
20	37	37: Farmers’ Pride in High-Quality cacao Production Increases	19	19: D Co. purchases fermented cacao at high price (3,000 rupiah/kg)
21	19	19: D Co. purchases fermented cacao at high price (3,000 rupiah/kg)	32	32: Provides great incentives for fermentation
22	32	32: Provides great incentives for fermentation	36	36: Construction of value-added distribution system
23	36	36: Construction of value-added distribution system	33	33: Sell unfermented if you want quick cash
24	33	33: Sell unfermented if you want quick cash	34	34: If you can spend time and effort, ferment and sell to D Co.
25	34	34: If you can spend time and effort, ferment and sell to D Co.	56	56: Strengthening cooperation with farmers through the local D Co.
26	56	56: Strengthening cooperation with farmers through the local D Co.	35	35: Farmers' options expand
27	35	35: Farmers' options expand	57	57: Increased purchase of high-quality cacao by D Co.
28	57	57: Increased purchase of high-quality cacao by D Co.	16	16: Technology development for high-quality production
29	16	16: Technology development for high-quality production	20	20: Using Compost
30	20	20: Using Compost	21	21: Technology development of ground cover by cacao leaves instead of herbicides
31	21	21: Technology development of ground cover by cacao leaves instead of herbicides	22	22: Developed a mechanism to prevent the pests by devising the tree arrangement
32	22	22: Developed a mechanism to prevent the pests by devising the tree arrangement	23	23: Attempts to cultivate pest-resistant strains
33	23	23: Attempts to cultivate pest-resistant strains	15	15: Control the use of chemical fertilizers and chemicals
34	15	15: Control the use of chemical fertilizers and chemicals	36	36: Improvement of farmland management technology
35	36	36: Improvement of farmland management technology	55	55: Providing high-quality fermented cacao
36	15	15: Control the use of chemical fertilizers and chemicals	40	40: Pest control is difficult
37	40	40: Pest control is difficult	41	41: Need to deepen ecosystem approach
38	35	35: Farmers' options expand	42	42: Need generation change of aged farmers
39	42	42: Need generation change of aged farmers	47	47: Create jobs for young generation in processing and distribution
40	47	47: Create jobs for young generation in processing and distribution	43	43: Improving sustainability as an industry
41	35	35: Farmers' options expand	60	60: Possibility of cacao farm tourism for D's customers
42	60	60: Possibility of cacao farm tourism for D's customers	61	61: Working with consumers who value their cacao
43	19	19: D Co. purchases fermented cacao at high price (3,000 rupiah/kg)	44	44: Extremely large market without requirement of fermentation
44	44	44: Extremely large market without requirement of fermentation	48	48: Function as a translator of U Co.
45	48	48: Function as a translator of U Co.	46	46: Movement to change the attitude of Company B

No.3 Improving cacao farm management

link_id	from_id	from_label	to_id	to_label
1	2	2: Growing international demand for cacao	3	3: Improving Farmers' Profits
2	3	3: Improving Farmers' Profits	4	4: Foster passive attitude to provide raw materials to major distributors
3	4	4: Foster passive attitude to provide raw materials to major distributors	5	5: Little capital accumulation
4	5	5: Little capital accumulation	18	18: Program development for farmer empowerment
5	1	1: Most cacao farmers are small-scale farmers of about 1 ha	6	6: Farmers are mainly in their 40s and 50s
6	6	6: Farmers are mainly in their 40s and 50s	7	7: Insufficient training of young farmers
7	7	7: Insufficient training of young farmers	13	13: Improving farm management and creating a system that can take pride in agriculture
8	10	10: Cacao production unstable due to climatic conditions	11	11: Emergence of migrant workers to neighbor countries' oil palm farms
9	11	11: Emergence of migrant workers to neighbor countries' oil palm farms	12	12: Farmland management during migrant work is neglected
10	12	12: Farmland management during migrant work is neglected	18	18: Program development for farmer empowerment
11	13	13: Improving farm management and creating a system that can take pride in agriculture	14	14: U Co. starts activities with farmers as equal partners
12	14	14: U Co. starts activities with farmers as equal partners	15	15: Major distributor B and Japanese chocolate maker D collaborate in various ways
13	15	15: Major distributor B and Japanese chocolate maker D collaborate in various ways	17	17: U Co.'s Mr.E appointed as coordinator of B Co.
14	17	17: U Co.'s Mr.E appointed as coordinator of B Co.	18	18: Program development for farmer empowerment
15	18	18: Program development for farmer empowerment	19	19: Formation of farmer groups
16	19	19: Formation of farmer groups	20	20: U Co.'s Mr.C provides excellent cacao cultivation technology
17	20	20: U Co.'s Mr.C provides excellent cacao cultivation technology	21	21: Strengthening the network of farmer groups
18	21	21: Strengthening the network of farmer groups	22	22: Create system to convey excellent cultivation techniques and management methods to farmers.
19	22	22: Create system to convey excellent cultivation techniques and management methods to farmers.	23	23: New method developed by farmers spreads through U Co.
20	23	23: New method developed by farmers spreads through U Co.	24	24: U Co. functions as a bidirectional translator
21	24	24: U Co. functions as a bidirectional translator	33	33: The emergence of advanced farmers 1
22	33	33: The emergence of advanced farmers 1	34	34: Farmer A forms a farmer group with 26 neighboring farmers
23	34	34: Farmer A forms a farmer group with 26 neighboring farmers	37	37: Prevention of erosion by terrace-like farmland management
24	37	37: Prevention of erosion by terrace-like farmland management	35	35: Obtained UTZ certification
25	35	35: Obtained UTZ certification	36	36: Half Farmers Start Fermentation at Home
26	36	36: Half Farmers Start Fermentation at Home	38	38: Management is smooth and stable
27	38	38: Management is smooth and stable	47	47: Fostering pride and pride in being a farmer
28	18	18: Program development for farmer empowerment	25	25: Hiring some farmers as field coordinators
29	25	25: Hiring some farmers as field coordinators	26	26: Emergence of new employment opportunities
30	26	26: Emergence of new employment opportunities	27	27: Promote participation of youth in the agricultural sector
31	24	24: U Co. functions as a bidirectional translator	28	28: Started production of high-quality fermented cacao required by D Co.
32	28	28: Started production of high-quality fermented cacao required by D Co.	29	29: U Co. collection of fermented cacao from farmers
33	29	29: U Co. collection of fermented cacao from farmers	30	30: Equipment provided by D Co.
34	30	30: Equipment provided by D Co.	31	31: Start of cacao mass processing and export by U Co.
35	31	31: Start of cacao mass processing and export by U Co.	32	32: Increased sales options for farmers
36	32	32: Increased sales options for farmers	46	46: The emergence of farmers with advanced business sense
37	45	45: Successful use of two sales channels, B Co. and D Co.	46	46: The emergence of farmers with advanced business sense
38	46	46: The emergence of farmers with advanced business sense	13	13: Improving farm management and creating a system that can take pride in agriculture
39	24	24: U Co. functions as a bidirectional translator	39	39: The emergence of advanced farmers 2
40	39	39: The emergence of advanced farmers 2	40	40: Farmer A's soil management using traditional techniques
41	40	40: Farmer A's soil management using traditional techniques	41	41: Controlling the use of chemical fertilizers and chemicals
42	41	41: Controlling the use of chemical fertilizers and chemicals	42	42: Reduction of expenses
43	42	42: Reduction of expenses	43	43: Expansion of farmland to 2 hectares
44	43	43: Expansion of farmland to 2 hectares	44	44: Ferment 30% of cacao and wholesale it to D Co.
45	44	44: Ferment 30% of cacao and wholesale it to D Co.	45	45: Successful use of two sales channels, B Co. and D Co.
46	47	47: Fostering pride and pride in being a farmer	21	21: Strengthening the network of farmer groups
47	27	27: Promote participation of youth in the agricultural sector	21	21: Strengthening the network of farmer groups
48	46	46: The emergence of farmers with advanced business sense	48	48: Only a small percentage of advanced farmers remain
49	48	48: Only a small percentage of advanced farmers remain	49	49: Insufficient business mindset
50	49	49: Insufficient business mindset	50	50: Necessity of changing farmers' mindset
51	21	21: Strengthening the network of farmer groups	52	52: Necessity of forming a farmers' union
52	32	32: Increased sales options for farmers	53	53: D Co.'s sales channel is small
53	53	53: D Co.'s sales channel is small	54	54: The beneficiaries are limited to one part
54	54	54: The beneficiaries are limited to one part	55	55: Need to expand high value-added distribution system

No.4 Multi-species cultivation on cacao farmland

link_id	from_id	from_label	to_id	to_label
1	4	4: International increase in cacao demand and monoculture spread	5	5: Mass injection of chemical fertilizers and chemicals becomes common
2	5	5: Mass injection of chemical fertilizers and chemicals becomes common	42	42: Degradation of soil and cacao bean quality
3	6	6: cacao harvest is twice a year and no income sometimes	7	7: Uncertain factors such as climate and price fluctuations
4	7	7: Uncertain factors such as climate and price fluctuations	44	44: Dry season's lack of water reduced the harvest
5	44	44: Dry season's lack of water reduced the harvest	8	8: Management of cacao production alone is unstable
6	8	8: Management of cacao production alone is unstable	14	14: U Co.'s activities to increase the potential value of cacao farms
7	55	55: cacao needs shade crops	15	15: Various crops can be grown in the forest floor
8	15	15: Various crops can be grown in the forest floor	56	56: Local farmers have originally cultivated many varieties
9	56	56: Local farmers have originally cultivated many varieties	48	48: Reviewing the value of multi-cultivation
10	14	14: U Co.'s activities to increase the potential value of cacao farms	20	20: Cultivate 14 crops including profitable crops
11	14	14: U Co.'s activities to increase the potential value of cacao farms	48	48: Reviewing the value of multi-cultivation
12	48	48: Reviewing the value of multi-cultivation	49	49: Organic cultivation using compost
13	42	42: Degradation of soil and cacao bean quality	48	48: Reviewing the value of multi-cultivation
14	49	49: Organic cultivation using compost	18	18: Take lemongrass and turmeric's therapeutic effects on cacao disease
15	18	18: Take lemongrass and turmeric's therapeutic effects on cacao disease	40	40: Reduce the use of chemicals and chemical fertilizers
16	20	20: Cultivate 14 crops including profitable crops	22	22: Securing diverse sources of income
17	22	22: Securing diverse sources of income	50	50: Found to lead to stable farm management
18	50	50: Found to lead to stable farm management	51	51: Multi-cultivation spreads to many farmers
19	51	51: Multi-cultivation spreads to many farmers	24	24: Development of new varieties
20	24	24: Development of new varieties	26	26: Ideas and attempts such as raising goats on cacao farms
21	26	26: Ideas and attempts such as raising goats on cacao farms	23	23: Farmers devise production methods to meet their conditions
22	23	23: Farmers devise production methods to meet their conditions	31	31: Income increases compared to monoculture farmers
23	31	31: Income increases compared to monoculture farmers	19	19: Stabilization of income through multi-cultivation
24	40	40: Reduce the use of chemicals and chemical fertilizers	43	43: Cost reduction
25	43	43: Cost reduction	30	30: Prevention of soil deterioration
26	30	30: Prevention of soil deterioration	32	32: Improving ecosystem functions and services in farm's environment
27	19	19: Stabilization of income through multi-cultivation	32	32: Improving ecosystem functions and services in farm's environment
28	32	32: Improving ecosystem functions and services in farm's environment	27	27: Increasing the resilience of farmers
29	27	27: Increasing the resilience of farmers	14	14: U Co.'s activities to increase the potential value of cacao farms
30	52	52: Extreme weather in 2017	53	53: The harvest of many crops has drastically decreased
31	53	53: The harvest of many crops has drastically decreased	35	35: The Need for Adaptation to Climate Change
32	35	35: The Need for Adaptation to Climate Change	23	23: Farmers devise production methods to meet their conditions
33	32	32: Improving ecosystem functions and services in farm's environment	41	41: Developing diverse products that are resilient to climate change
34	41	41: Developing diverse products that are resilient to climate change	54	54: Increased resilience to extreme weather events
35	32	32: Improving ecosystem functions and services in farm's environment	60	60: Diverse products provide attractive ingredients
36	60	60: Diverse products provide attractive ingredients	61	61: Improving the attractiveness of cacao farmland ecosystems
37	61	61: Improving the attractiveness of cacao farmland ecosystems	62	62: Possibility of promoting tourism on cacao farms

No.5 Development of cacao farm tourism

link_id	from_id	from_label	to_id	to_label
1	1	1: Polewari's main industries are agriculture and fishing	2	2: Not blessed with tourism resources
2	2	2: Not blessed with tourism resources	3	3: Only a few attempts to develop tourism resources
3	3	3: Only a few attempts to develop tourism resources	5	5: Progress of multi-cultivation on cacao farms
4	5	5: Progress of multi-cultivation on cacao farms	6	6: Providing diverse and attractive ingredients
5	6	6: Providing diverse and attractive ingredients	7	__7: Recognize possibility of tourism use of environment friendly cacao farming
6	7	__7: Recognize possibility of tourism use of environment friendly cacao farming	12	__12: Increased interest in cacao farm experience tourism
7	8	8: Strength cooperation with D Co. through high-quality cacao production	9	9: Improve attractiveness of D Co.'s products to consumers
8	9	9: Improve attractiveness of D Co.'s products to consumers	10	10: Japanese consumers find value in connection D Co. and farmers
9	10	10: Japanese consumers find value in connection D Co. and farmers	11	11: Grow interest in cacao farmers' production sites
10	11	11: Grow interest in cacao farmers' production sites	13	13: D Co.'s president visited Polewari in 2011
11	13	13: D Co.'s president visited Polewari in 2011	14	14: D Co.'s companion pleased the farm experience
12	14	14: D Co.'s companion pleased the farm experience	15	15: U Co. emphasizes appeal of cacao farm experience tourism
13	12	__12: Increased interest in cacao farm experience tourism	15	15: U Co. emphasizes appeal of cacao farm experience tourism
14	15	15: U Co. emphasizes appeal of cacao farm experience tourism	16	16: Japanese can potential tourists in farm tourism
15	16	16: Japanese can potential tourists in farm tourism	17	17: D Co. plans to visit cacao farms in Japan
16	17	17: D Co. plans to visit cacao farms in Japan	44	44: Prefectural government also supports cacao farm tourism
17	44	44: Prefectural government also supports cacao farm tourism	18	18: Since 2014, Japanese consumers visited Polewari once a year
18	18	18: Since 2014, Japanese consumers visited Polewari once a year	19	19: Twice a year from 2016, a total of about 100 people visited
19	19	19: Twice a year from 2016, a total of about 100 people visited	20	20: Mr.C's farm accepts tourists
20	20	20: Mr.C's farm accepts tourists	21	21: Cacao farm tour
21	21	21: Cacao farm tour	22	22: Raw cacao tasting
22	22	22: Raw cacao tasting	23	23: Seedling planting
23	23	23: Seedling planting	24	24: Experience of the fermentation process
24	24	24: Experience of the fermentation process	25	25: Interaction with farmers and home cooking
25	25	25: Interaction with farmers and home cooking	28	28: Direct contact between farmers and consumers
26	20	20: Mr.C's farm accepts tourists	26	26: General consumers, students, and industry stakeholders participate
27	26	26: General consumers, students, and industry stakeholders participate	27	27: Neighboring farmers of Mr. C also participate in acceptance
28	27	27: Neighboring farmers of Mr. C also participate in acceptance	28	28: Direct contact between farmers and consumers
29	28	28: Direct contact between farmers and consumers	29	29: Interaction with consumers stimulates farmers
30	29	29: Interaction with consumers stimulates farmers	30	30: Pride in their products
31	30	30: Pride in their products	31	31: Strong motivation to improve the quality of farming
32	31	31: Strong motivation to improve the quality of farming	32	32: Mindset change with consumers
33	32	32: Mindset change with consumers	33	33: Movement to curb the use of chemicals
34	33	33: Movement to curb the use of chemicals	4	4: Expansion of environmental conservation type cacao farm management
35	4	4: Expansion of environmental conservation type cacao farm management	7	__7: Recognize possibility of tourism use of environment friendly cacao farming
36	29	29: Interaction with consumers stimulates farmers	34	34: Strengthening ties between farmers and consumers
37	34	34: Strengthening ties between farmers and consumers	35	35: Growing concern in cacao production overseas
38	35	35: Growing concern in cacao production overseas	36	36: Change in consumer behavior
39	36	36: Change in consumer behavior	37	37: Farmers notice consumers who value their cacao
40	37	37: Farmers notice consumers who value their cacao	47	47: Improve farm management and production processes for consumer expectations
41	47	47: Improve farm management and production processes for consumer expectations	38	38: Community formation among producers and consumers by D Co.
42	38	38: Community formation among producers and consumers by D Co.	7	__7: Recognize possibility of tourism use of environment friendly cacao farming
43	29	29: Interaction with consumers stimulates farmers	39	39: Build mechanism many farmers can participate is necessary
44	39	39: Build mechanism many farmers can participate is necessary	40	40: Farmer guide training and possibility of farmhouse inn
45	40	40: Farmer guide training and possibility of farmhouse inn	41	41: Tourism may become a new livelihood option
46	44	44: Prefectural government also supports cacao farm tourism	45	45: Possibility to promote wide-area tourism
47	45	45: Possibility to promote wide-area tourism	46	46: Expanding beneficiaries and participants is a challenge
48	31	31: Strong motivation to improve the quality of farming	48	48: Utilization as tourism resource may improve quality of farm
49	48	48: Utilization as tourism resource may improve quality of farm	42	42: Risk of overuse as a tourism resource
50	42	42: Risk of overuse as a tourism resource	43	43: How tourism should not degrade the quality of farms

No.6 Collaborative network construction

link_id	from_id	from_label	to_id	to_label
1	1	1: Increasing charities to improve local children's nutrition (1998-)	3	3: Composition of NGO (2003)
2	3	3: Composition of NGO (2003)	2	2: Building a network with farmers through charity activities
3	2	2: Building a network with farmers through charity activities	4	4: Expansion of activities
4	4	4: Expansion of activities	51	51: Creating a network to eliminate poverty in downstream areas
5	11	11: Maintenance of irrigation canals from the Bilibili Dam (2007)	12	12: Completion of secondary waterway for bilibili water (upstream)
6	12	12: Completion of secondary waterway for bilibili water (upstream)	13	13: Solving water shortages in the dry season (upstream)
7	13	13: Solving water shortages in the dry season (upstream)	20	20: Irrigation channel management not in place (upstream)
8	15	15: Improving the lives of farmers	18	18: Problem of irrigated water not reaching farmers end waterway
9	20	20: Irrigation channel management not in place (upstream)	50	50: Difficult to maintain waterways
10	50	50: Difficult to maintain waterways	18	18: Problem of irrigated water not reaching farmers end waterway
11	18	18: Problem of irrigated water not reaching farmers end waterway	19	19: Unfairness between upstream and downstream farmers
12	19	19: Unfairness between upstream and downstream farmers	51	51: Creating a network to eliminate poverty in downstream areas
13	51	51: Creating a network to eliminate poverty in downstream areas	52	52: Construction of tertiary waterways utilizing networks
14	52	52: Construction of tertiary waterways utilizing networks	24	24: Farmers still unable to reach water
15	24	24: Farmers still unable to reach water	28	28: Participation and collaboration of diverse stakeholders
16	28	28: Participation and collaboration of diverse stakeholders	29	29: Building a system to deliver water to downstream farmers
17	29	29: Building a system to deliver water to downstream farmers	31	31: Strengthening water conservancy associations
18	31	31: Strengthening water conservancy associations	30	30: Create mechanism to reflect farmers' voices on water distribution
19	30	30: Create mechanism to reflect farmers' voices on water distribution	34	34: Holding meetings with diverse stakeholders
20	34	34: Holding meetings with diverse stakeholders	53	53: Examination of mechanism for reaching water at the very end
21	53	53: Examination of mechanism for reaching water at the very end	35	35: Scheduling mechanism through collaboration with diverse stakeholders
22	35	35: Scheduling mechanism through collaboration with diverse stakeholders	36	36: Agreement on sluice schedule from up to downstream
23	36	36: Agreement on sluice schedule from up to downstream	38	38: Completion of manual and utilization by translator
24	38	38: Completion of manual and utilization by translator	39	39: Builde foundation for communication for water to reach very end
25	39	39: Builde foundation for communication for water to reach very end	48	48: Providing stakeholders with opportunities for interaction and dialogue
26	48	48: Providing stakeholders with opportunities for interaction and dialogue	47	47: Collaborative network expands
28	40	40: Distribution of irrigation water to the terminal area	41	41: Bifurcation is possible even in the terminal region
29	41	41: Bifurcation is possible even in the terminal region	15	15: Improving the lives of farmers
30	15	15: Improving the lives of farmers	42	42: Still some farmers have no access to water in other end areas
31	42	42: Still some farmers have no access to water in other end areas	43	43: Need for wide-area expansion to end regions
32	43	43: Need for wide-area expansion to end regions	44	44: In particular, the use of manuals is an important means
33	44	44: In particular, the use of manuals is an important means	45	45: Utilization of manuals by excellent translators in other end regions
34	45	45: Utilization of manuals by excellent translators in other end regions	46	46: Network enhancement to facilitate dialogue
35	47	47: Collaborative network expands	28	28: Participation and collaboration of diverse stakeholders
36	38	38: Completion of manual and utilization by translator	49	49: Open and close sluice gate mechanism where water reaches the very end first
37	49	49: Open and close sluice gate mechanism where water reaches the very end first	40	40: Distribution of irrigation water to the terminal area

No.7 Waste recycling and Tourism development

link_id	from_id	from_label	to_id	to_label
1	1	1: Rice cultivation flourish at top irrigation channel	2	2: Conflicts among farmers over water resources
2	2	2: Conflicts among farmers over water resources	3	3: Large amount of waste in the waterway
3	3	3: Large amount of waste in the waterway	4	4: Mr.Z collaborates NGO as water use association head
4	4	4: Mr.Z collaborates NGO as water use association head	5	5: Build up dialogue with farmers
5	5	5: Build up dialogue with farmers	6	6: Recognize water shortage at downstream area by mass use in upstream
6	6	6: Recognize water shortage at downstream area by mass use in upstream	7	7: Various efforts to deliver water to downstream
7	7	7: Various efforts to deliver water to downstream	8	8: Scheduling for opening and closing gates
8	8	8: Scheduling for opening and closing gates	9	9: Recognize needs of treating waste hindering water flow
9	9	9: Recognize needs of treating waste hindering water flow	10	10: Successful attempt to deliver water downstream
10	10	10: Successful attempt to deliver water downstream	11	11: Mr.Z gains trust of villagers to promote actions as a village head
11	11	11: Mr.Z gains trust of villagers to promote actions as a village head	12	____12: Meeting an innovator in Malawi
12	12	____12: Meeting an innovator in Malawi	13	13: Inspired by the recycling attempt of Chembe village
13	13	13: Inspired by the recycling attempt of Chembe village	14	14: Efforts to realize tourism village
14	14	14: Efforts to realize tourism village	15	15: Promotion of waste reduction to improve landscape
15	15	15: Promotion of waste reduction to improve landscape	16	16: Demonstrate leadership in reducing waste in waterways
16	16	16: Demonstrate leadership in reducing waste in waterways	17	17: Focus on economic and health values of Moringa
17	17	17: Focus on economic and health values of Moringa	18	18: Plant Moringa trees on banks with accumulated wastes
18	18	18: Plant Moringa trees on banks with accumulated wastes	19	19: Effective on landscape improvement and waste control
19	19	19: Effective on landscape improvement and waste control	20	20: Expand tree planting to small waterways
20	20	20: Expand tree planting to small waterways	21	21: Allocate bank plots to households to plant Moringa
21	21	21: Allocate bank plots to households to plant Moringa	22	22: Fostering villagers' Sence of Ownership
22	14	14: Efforts to realize tourism village	23	23: Develop a park with bamboo forests for villagers to gather
23	23	23: Develop a park with bamboo forests for villagers to gather	24	24: Develop farms to grow vegetables and flowers with economic value
24	24	24: Develop farms to grow vegetables and flowers with economic value	25	25: Improve landscape of residential areas through planting
25	25	25: Improve landscape of residential areas through planting	26	26: Progress in improving the landscape of the village
26	26	26: Progress in improving the landscape of the village	27	27: Set up trash cans in the village
27	27	27: Set up trash cans in the village	28	28: Attempts to produce and sell crafts using waste
28	28	28: Attempts to produce and sell crafts using waste	29	29: Attempts to foster a recycling industry in the village
29	29	29: Attempts to foster a recycling industry in the village	30	30: Production of bricks with low environmental impact using rice husk
30	30	30: Production of bricks with low environmental impact using rice husk	31	31: Production of furniture by reusing building waste materials
31	31	31: Production of furniture by reusing building waste materials	32	32 : Develop activities through village under Mr.Z lead
32	32	32 : Develop activities through village under Mr.Z lead	33	33: Creating tourism resources by waste and landscape management
33	33	33: Creating tourism resources by waste and landscape management	49	49: People's awareness of the landscape has changed
34	49	49: People's awareness of the landscape has changed	41	41: Progress in landscape management by villagers themselves
35	41	41: Progress in landscape management by villagers themselves	22	22: Fostering villagers' Sence of Ownership
36	22	22: Fostering villagers' Sence of Ownership	35	35: Progress in developing sustainable industries in villages
37	35	35: Progress in developing sustainable industries in villages	36	36: Tourist effects such as student training tours occur
38	22	22: Fostering villagers' Sence of Ownership	37	37: Promote cooperation with neighboring villages to plant Moringa
39	37	37: Promote cooperation with neighboring villages to plant Moringa	38	38: Wide-area deployment covering the Moringa planting basin
40	36	36: Tourist effects such as student training tours occur	39	39: Job creation and income generation by industrial development
41	39	39: Job creation and income generation by industrial development	40	40: Moringa planting and landscape management win award
42	40	40: Moringa planting and landscape management win award	11	11: Mr.Z gains trust of villagers to promote actions as a village head
43	24	24: Develop farms to grow vegetables and flowers with economic value	42	42: Trial of sustainable and environmentally friendly integrated agriculture
44	42	42: Trial of sustainable and environmentally friendly integrated agriculture	43	43: Promotion of environmentally friendly agriculture is an issue
45	43	43: Promotion of environmentally friendly agriculture is an issue	44	44: Important agricultural exchange with Chembe
46	14	14: Efforts to realize tourism village	45	45: Big impact of International exchange of innovators
47	45	45: Big impact of International exchange of innovators	46	46: Needs of international exchange mechanism of innovators
48	32	32 : Develop activities through village under Mr.Z lead	47	47: Needs of ideas combine waste reduction and tourism
49	47	47: Needs of ideas combine waste reduction and tourism	48	48: Needs of a mechanism to continuously promote new ideas

No.8 Improvement of rice planting method through international exchange

link_id	from_id	from_label	to_id	to_label
1	1	1 The terraced rice field landscape is UNESCO World Cultural Heritage	2	2 Rice cultivation in rice terraces alone does not provide sufficient income
2	2	2 Rice cultivation in rice terraces alone does not provide sufficient income	3	3 Labor-centered generation flows out to urban areas
3	3	3 Labor-centered generation flows out to urban areas	5	5 Expansion of abandoned cultivated land and collapse of terraced rice fields
4	5	5 Expansion of abandoned cultivated land and collapse of terraced rice fields	8	8 Crisis of agricultural continuation and impact on local safety
5	8	8 Crisis of agricultural continuation and impact on local safety	9	9 Negative impact on tourism industry
6	9	9 Negative impact on tourism industry	24	24 Efforts to increase income in terraced rice fields
7	24	24 Efforts to increase income in terraced rice fields	29	29 Meeting various people through international exchange projects
8	29	29 Meeting various people through international exchange projects	37	37 Emergence of attitude to incorporate external opinions
9	37	37 Emergence of attitude to incorporate external opinions	10	10 Sharing methods for efficient use of rice terraces in Japan
10	10	10 Sharing methods for efficient use of rice terraces in Japan	25	25 Proposal for change of planting interval and depth
11	25	25 Proposal for change of planting interval and depth	26	26 Proposal changing amount of water in rice terraces
12	26	26 Proposal changing amount of water in rice terraces	12	12 Determination of trial of proposal contents
13	12	12 Determination of trial of proposal contents	31	31 Decision to trial with native species
14	31	31 Decision to trial with native species	13	13 Start of trial in part of own rice terraces
15	13	13 Start of trial in part of own rice terraces	14	14 Maintain the conventional method in other rice terraces
16	14	14 Maintain the conventional method in other rice terraces	15	15 Less impact on overall harvest
17	15	15 Less impact on overall harvest	18	18 trials are continuing
18	13	13 Start of trial in part of own rice terraces	27	27 No recommend to poor farmers until sure of success
19	27	27 No recommend to poor farmers until sure of success	28	28 No risk for poor farmers
20	28	28 No risk for poor farmers	30	30 No opposite, but low interest of surrounding farmers
21	30	30 No opposite, but low interest of surrounding farmers	16	16 Farmers around the area who help appear
22	16	16 Farmers around the area who help appear	17	17 Expansion of farmer network
23	17	17 Expansion of farmer network	20	20 Possibility of leading to maintenance of terraced rice landscape
24	18	18 trials are continuing	19	19Potential increase in rice yield and income
25	19	19Potential increase in rice yield and income	17	17 Expansion of farmer network
26	20	20 Possibility of leading to maintenance of terraced rice landscape	22	22 Possibility of increasing safety in the community
27	22	22 Possibility of increasing safety in the community	23	23 Potential to lead to the maintenance of tourism
28	23	23 Potential to lead to the maintenance of tourism	24	24 Efforts to increase income in terraced rice fields
29	19	19Potential increase in rice yield and income	35	35 Necessity of scientific verification of trial results
30	17	17 Expansion of farmer network	36	36 Improving the quality of life of individual farmers
31	36	36 Improving the quality of life of individual farmers	50	50 Improving motivation to continue rice cultivation in rice terraces
32	50	50 Improving motivation to continue rice cultivation in rice terraces	51	51 Stop the decline in rice farmers
33	51	51 Stop the decline in rice farmers	32	32 Activation of rice cultivation in many farmers
34	32	32 Activation of rice cultivation in many farmers	33	33 Reduction of abandoned cultivated land
35	33	33 Reduction of abandoned cultivated land	52	52 Improvement of rice terrace management

No.9 Diversification of production activities of natural rubber plantations

link_id	from_id	from_label	to_id	to_label
1	1	1: Local natural rubber production expands	2	2: Needs for highly technical tappers
2	2	2: Needs for highly technical tappers	40	40: Emergence of female Tapper (mother)
3	40	40: Emergence of female Tapper (mother)	5	5: Production and sales of rubber seeds and seedlings by grafting
4	5	5: Production and sales of rubber seeds and seedlings by grafting	6	6: Accumulation of capital
6	6	6: Accumulation of capital	9	9: Purchase of thin land
7	9	9: Purchase of thin land	10	10: Land improvement through banana production
8	10	10: Land improvement through banana production	11	11: Accumulate capital in production of cucumbers, chili, etc.
9	11	11: Accumulate capital in production of cucumbers, chili, etc.	101	101: Development of natural rubber plantations
10	101	101: Development of natural rubber plantations	39	39: Need support employee families' live
11	39	39: Need support employee families' live	102	102: Start efforts to stabilize income
12	15	15: Unstable natural rubber production in the dry season	102	102: Start efforts to stabilize income
13	102	102: Start efforts to stabilize income	12	12: Construction for processing (smoking) facilities
14	12	12: Construction for processing (smoking) facilities	6	6: Accumulation of capital
15	102	102: Start efforts to stabilize income	99	99: Utilization of processing (smoking) facilities
16	99	99: Utilization of processing (smoking) facilities	13	13: Expansion of natural rubber production
17	13	13: Expansion of natural rubber production	16	16: Durian cultivation
18	16	16: Durian cultivation	17	17: Mahogany, Longkon, Ginger cultivation
19	17	17: Mahogany, Longkon, Ginger cultivation	19	19: Mosouchiku cultivation
20	19	19: Mosouchiku cultivation	20	20: Realization of crop diversification
21	20	20: Realization of crop diversification	23	23: Secure stable income in dry season
22	23	23: Secure stable income in dry season	27	27: Realization of modern farm management
23	27	27: Realization of modern farm management	102	102: Start efforts to stabilize income
24	102	102: Start efforts to stabilize income	100	100: Business expansion such as apartment management
25	100	100: Business expansion such as apartment management	21	21: Realization of diversified management
26	21	21: Realization of diversified management	32	32: Employment of surrounding micro-farmers when prices are low
27	32	32: Employment of surrounding micro-farmers when prices are low	30	30: Enhance residence and tuition support for high-skill employees
28	30	30: Enhance residence and tuition support for high-skill employees	40	40: The condition of the trees improves
29	40	40: The condition of the trees improves	25	25: Realization of latex production in the dry season
30	25	25: Realization of latex production in the dry season	24	24: Realization of high-quality rubber production
31	24	24: Realization of high-quality rubber production	41	41: No need to join a union
32	41	41: No need to join a union	26	26: Direct deal with compression plant
33	26	26: Direct deal with compression plant	27	27: Realization of modern farm management
34	27	27: Realization of modern farm management	37	37: Increase added value through end product production
35	99	99: Utilization of processing (smoking) facilities	42	42: Reduction of environmental load by wastewater treatment

No.10 Reorganization and utilization of traditional salt making techniques

link_id	from_id	from_label	to_id	to_label
1	1	1: Wai district, dig holes in tidal flats to collect saltwater since ancient	2	2: Salt production by boiling down wood from coastal mangrove forests is popular
2	2	2: Salt production by boiling down wood from coastal mangrove forests is popular	6	6: Traditional salt production gradually forgotten
3	6	6: Traditional salt production gradually forgotten	8	8: Translator function by conservation organization
4	8	8: Translator function by conservation organization	11	______11: Cross-generational collaborative efforts to revive traditional culture
5	11	______11: Cross-generational collaborative efforts to revive traditional culture	9	9: In 2000, efforts to revive salt production in the entire village began
6	9	9: In 2000, efforts to revive salt production in the entire village began	10	10: Skill acquisition by seven relative women group from village
7	10	10: Skill acquisition by seven relative women group from village	12	12: Waste utilization, introduction of aluminum pots, ingenuity to wrap products in foil
8	12	12: Waste utilization, introduction of aluminum pots, ingenuity to wrap products in foil	13	13: Traditional basket with hand-knitted mangrove branches
9	13	13: Traditional basket with hand-knitted mangrove branches	14	14: Miniaturization to meet the needs of tourists
10	14	14: Miniaturization to meet the needs of tourists	15	15: Use dead trees and other tree species as firewood
11	15	15: Use dead trees and other tree species as firewood	16	16: Reduce the load on resources such as mangrove forests
12	11	______11: Cross-generational collaborative efforts to revive traditional culture	18	18: Young members start promoting online
13	18	18: Young members start promoting online	19	19: Attract tourists and earn cash income by selling salt
14	19	19: Attract tourists and earn cash income by selling salt	20	20: Salt is sold to villagers and medical institutions as a "health food"
15	16	16: Reduce the load on resources such as mangrove forests	25	25: Male participation and network expansion
16	20	20: Salt is sold to villagers and medical institutions as a "health food"	21	21: In 2016, the salt-making committee was reorganized under a male chairperson.
17	21	21: In 2016, the salt-making committee was reorganized under a male chairperson.	24	24: Cooperation of "Zongo", the decision-making body of the village
18	24	24: Cooperation of "Zongo", the decision-making body of the village	27	27: In 2017, a salt factory was developed
19	27	27: In 2017, a salt factory was developed	25	25: Male participation and network expansion
20	25	25: Male participation and network expansion	45	45: New well was dug near salt factory
21	45	45: New well was dug near salt factory	26	26: Strengthening the network of people
22	26	26: Strengthening the network of people	28	28: Committee manages visitor's admission fees and salt sales (account book)
23	28	28: Committee manages visitor's admission fees and salt sales (account book)	29	29: Most proceeds allocate to activities of the committee
24	29	29: Most proceeds allocate to activities of the committee	30	30: Build foundation for sustainable implementation of traditional salt production begun
25	11	______11: Cross-generational collaborative efforts to revive traditional culture	22	22: Collaboration with wide-area relatives such as local governments, and neighbor Kumbuna.
26	22	22: Collaboration with wide-area relatives such as local governments, and neighbor Kumbuna.	39	39: Possibility of reorganizing wide-area kinship networks
27	21	21: In 2016, the salt-making committee was reorganized under a male chairperson.	23	23: Enhanced collaboration with travel agencies
28	23	23: Enhanced collaboration with travel agencies	36	36: Hotels and travel agencies listen to reputaion and approach village
29	36	36: Hotels and travel agencies listen to reputaion and approach village	45	45: Discussions in the village
30	45	45: Discussions in the village	37	37: Selling to tourists business, "tourism salt making business," is beginning to develop
31	37	37: Selling to tourists business, "tourism salt making business," is beginning to develop	33	33: Gradual increase in sales over the past 5 years
32	30	30: Build foundation for sustainable implementation of traditional salt production begun	31	31: Revival of traditional salt production
33	31	31: Revival of traditional salt production	32	32: Pride and attachment to traditional culture grow among people
34	32	32: Pride and attachment to traditional culture grow among people	11	______11: Cross-generational collaborative efforts to revive traditional culture
35	33	33: Gradual increase in sales over the past 5 years	34	34: The possibility of improving people's lives through the tourism industry, etc. was born
36	34	34: The possibility of improving people's lives through the tourism industry, etc. was born	19	19: Attract tourists and earn cash income by selling salt
37	37	37: Selling to tourists business, "tourism salt making business," is beginning to develop	40	40: Further increase sales and expand financial base of salt-making committee
38	25	25: Male participation and network expansion	41	41: Need to stably supply wages, etc. for villagers who are responsible for heavy labor
39	41	41: Need to stably supply wages, etc. for villagers who are responsible for heavy labor	42	42: Women's groups involved in salt production alone cannot secure enough work time
40	42	42: Women's groups involved in salt production alone cannot secure enough work time	43	43: Requires equipment to increase work efficiency such as pumps and chainsaws
41	40	40: Further increase sales and expand financial base of salt-making committee	44	44: Further ingenuity is needed in the method of profit sharing

No.11 Small-scale aquaculture and multi-species cultivation

link_id	from_id	from_label	to_id	to_label
1	1	1: Lack of animal protein sources and cash resources of farmers	2	2: Government recommends small-scale aquaculture
2	2	2: Government recommends small-scale aquaculture	3	3: Slow growth of fish and no sales mechanism
3	3	3: Slow growth of fish and no sales mechanism	4	4: Small-scale aquaculture does not spread
4	4	4: Small-scale aquaculture does not spread	10	10: Practice of multi-cultivation and aquaculture with own ideas
5	5	5: Agricultural subjects in junior high school are substantial	6	6: Practice of agricultural practice
6	6	6: Practice of agricultural practice	7	7: Learn about multi-cultivation and aquaculture
7	7	7: Learn about multi-cultivation and aquaculture	8	8: It does not lead to practice
8	8	8: It does not lead to practice	9	9: Persuade father based on learning in junior high school at age 14
9	9	9: Persuade father based on learning in junior high school at age 14	10	10: Practice of multi-cultivation and aquaculture with own ideas
10	10	10: Practice of multi-cultivation and aquaculture with own ideas	11	11: Use abundant underground water as a water source
11	11	11: Use abundant underground water as a water source	12	12: Connect multiple aquaculture ponds in a cascade type
12	12	12: Connect multiple aquaculture ponds in a cascade type	13	13: The top pond has water even in the dry season
13	13	13: The top pond has water even in the dry season	17	17: Challenges in selecting fish species and cultivated crops
14	17	17: Challenges in selecting fish species and cultivated crops	18	18: Repeat trial and error
15	14	14: Technical guidance from the Fisheries Bureau and NGOs	15	15: Assistance is unstable
16	15	15: Assistance is unstable	16	16: Not getting the results I want
17	16	16: Not getting the results I want	18	18: Repeat trial and error
18	18	18: Repeat trial and error	19	19: Banana cultivation
19	19	19: Banana cultivation	20	20: Growing pineapple and avocado
20	20	20: Growing pineapple and avocado	21	21: Responding to changes in market demand
21	21	21: Responding to changes in market demand	22	22: Trial cultivation of guava and sugarcane
22	22	22: Trial cultivation of guava and sugarcane	23	23: Successful large-scale cultivation of pineapple
23	23	23: Successful large-scale cultivation of pineapple	24	24: Use of sugar cane to strengthen pond embankments
24	24	24: Use of sugar cane to strengthen pond embankments	32	32: Improvement of management technology for aquaculture ponds
25	24	24: Use of sugar cane to strengthen pond embankments	49	49: Strong awareness of economies of scale
26	32	32: Improvement of management technology for aquaculture ponds	47	47: Practice of small-scale irrigation in the dry season
27	47	47: Practice of small-scale irrigation in the dry season	41	41: Cultivate different crops in different seasons
28	18	18: Repeat trial and error	25	25: Start farming locally available fish species
29	25	25: Start farming locally available fish species	26	26: Introduction of fish species recommended by the Fisheries Bureau
30	26	26: Introduction of fish species recommended by the Fisheries Bureau	28	28: Establishment of aquaculture schedule
31	32	32: Improvement of management technology for aquaculture ponds	29	29: Sold in the dry season when the lake is less landed
32	28	28: Establishment of aquaculture schedule	32	32: Improvement of management technology for aquaculture ponds
33	29	29: Sold in the dry season when the lake is less landed	30	30: Learn and utilize your own manufacturing technology
34	30	30: Learn and utilize your own manufacturing technology	31	31: Profits from aquaculture
35	31	31: Profits from aquaculture	34	34: Realizing diversified management
36	49	49: Strong awareness of economies of scale	33	33: Profit improvement with scale expansion
37	33	33: Profit improvement with scale expansion	34	34: Realizing diversified management
38	34	34: Realizing diversified management	40	40: Centralized production of the most profitable crops
39	40	40: Centralized production of the most profitable crops	41	41: Cultivate different crops in different seasons
40	41	41: Cultivate different crops in different seasons	42	42: Stable income throughout the year
41	42	42: Stable income throughout the year	10	10: Practice of multi-cultivation and aquaculture with own ideas
42	34	34: Realizing diversified management	35	35: Strengthening cooperation with neighboring farmers
43	35	35: Strengthening cooperation with neighboring farmers	36	36: Building a system to learn from each other
44	36	36: Building a system to learn from each other	37	37: Cultivation trial of new crops
45	37	37: Cultivation trial of new crops	38	38: Enjoying a new attempt
46	38	38: Enjoying a new attempt	39	39: Fostering pride as a farmer
47	39	39: Fostering pride as a farmer	10	10: Practice of multi-cultivation and aquaculture with own ideas
48	34	34: Realizing diversified management	44	44: Stabilization of management in response to changes in demand, etc.
49	31	31: Profits from aquaculture	45	45: Devising sales channels for farmed fish
50	35	35: Strengthening cooperation with neighboring farmers	46	46: Organizing farmers and sharing management skills
51	46	46: Organizing farmers and sharing management skills	49	49: Strengthening collaboration with scientists
52	47	47: Practice of small-scale irrigation in the dry season	48	48: Expansion of irrigation scale

No.12 Seasonal fishing bans around Mbenji Island by traditional chiefs and communities

link_id	from_id	from_label	to_id	to_label
1	1	1: Rich in fishing ground around the island	51	51: Open access to fishing ground
2	51	51: Open access to fishing ground	3	3: Increace in number of access to the fishing ground
3	3	3: Increace in number of access to the fishing ground	7	7: Rise of risks to the fishermen
4	7	7: Rise of risks to the fishermen	17	17: Shift to other subsistence in the rainy season
5	17	17: Shift to other subsistence in the rainy season	9	9: Practice of prohibited fishing in the rainy season
6	12	12: Recognition of the island as a sacred place	29	29: Local enforcement (governing and controlling)
7	9	9: Practice of prohibited fishing in the rainy season	15	15: Limited fishing activities during the rainy season
8	15	15: Limited fishing activities during the rainy season	13	13: Less frequency of reaching out to the island
9	13	13: Less frequency of reaching out to the island	16	16: Decreasing of fishing pressure during the rainy season
10	16	16: Decreasing of fishing pressure during the rainy season	18	18: Utaka’s spawning season protection in rainy season
11	18	18: Utaka’s spawning season protection in rainy season	19	19: Stabilization of utaka resources
12	19	19: Stabilization of utaka resources	21	21: Recognition of utaka resources being in good condition
13	21	21: Recognition of utaka resources being in good condition	22	22: Recognition of resources’ being in good condition as a result of efforts to prohibit fishing
14	22	22: Recognition of resources’ being in good condition as a result of efforts to prohibit fishing	23	23: Increasing awareness of local ties
15	23	23: Increasing awareness of local ties	24	24: Increasing awareness of local pride
16	24	24: Increasing awareness of local pride	26	26: Increasing reliability of traditional leaders
17	26	26: Increasing reliability of traditional leaders	32	32: External actor`s collaboration (govenments, NGOs, etc)
18	32	32: External actor`s collaboration (govenments, NGOs, etc)	31	31: Demonstration of translator function
19	31	31: Demonstration of translator function	33	33: Increasing opportunities to select and accept information and knowledge
20	33	33: Increasing opportunities to select and accept information and knowledge	10	10: Recongnision of the rainy season as utaka's spawning season
21	10	10: Recongnision of the rainy season as utaka's spawning season	11	11: Prohibited fishing leads to preserve utaka's spawning season
22	11	11: Prohibited fishing leads to preserve utaka's spawning season	29	29: Local enforcement (governing and controlling)
23	29	29: Local enforcement (governing and controlling)	9	9: Practice of prohibited fishing in the rainy season
24	32	32: External actor`s collaboration (govenments, NGOs, etc)	100	100: Disseminating the values of local resource management practices
25	100	100: Disseminating the values of local resource management practices	54	54: Impact on national policies and other communities
26	29	29: Local enforcement (governing and controlling)	53	53: Existence of advisory committee
27	53	53: Existence of advisory committee	50	50: A solemn ceremony and review of fishing activities
28	50	50: A solemn ceremony and review of fishing activities	48	48: Penalties for violators
29	48	48: Penalties for violators	55	55: Recognition that prohibited fishing is not always appropriate
30	55	55: Recognition that prohibited fishing is not always appropriate	49	49: System for measuring and registering the mesh size of fishing nets
31	49	49: System for measuring and registering the mesh size of fishing nets	30	30: Trial of more appropriate and feasible resource management tools
32	30	30: Trial of more appropriate and feasible resource management tools	26	26: Increasing reliability of traditional leaders
33	19	19: Stabilization of utaka resources	41	41: Practice and realization of value-added distribution
34	41	41: Practice and realization of value-added distribution	43	43: Securing profits commensurate with value-added distribution

No.13 Formation and operation of a tour guide association by local residents

link_id	from_id	from_label	to_id	to_label
1	1	1: Chembe village in a tourist area	2	2: There are many tourist lodges
2	2	2: There are many tourist lodges	3	3: Increase in tourists
3	3	3: Increase in tourists	4	4: Emergence of souvenir sellers and self-proclaimed tour guides
4	4	4: Emergence of souvenir sellers and self-proclaimed tour guides	5	5: A villager's guide to theft damage was suspected
5	5	5: A villager's guide to theft damage was suspected	7	7: Occurrence of feuds with lodges
6	7	7: Occurrence of feuds with lodges	8	8: Growing desire to be recognized as a proper career as a tour guide
7	10	____10: Movement of lodges to build good relationships with village	12	12: Guided discussion
8	12	12: Guided discussion	13	13: Three young people formed a tour guide association
9	13	13: Three young people formed a tour guide association	17	17: Lodge supports movement
10	14	14: Malawi entry visa price goes up	15	15: Fewer tourists from abroad
11	15	15: Fewer tourists from abroad	16	16: Domestic tourists mainstream
12	16	16: Domestic tourists mainstream	42	42: Improving the quality of tourists
13	18	18: 56 guides (all villagers) join the union	22	22: Tour fee is set uniformly
14	19	19: Responsible for guiding guests at each lodge on a rotating basis	20	20: Revenues are evenly distributed
15	18	18: 56 guides (all villagers) join the union	38	38: Job creation
16	38	38: Job creation	29	29: Avoid excessive competition by limiting the number of guides
17	23	23: Union members pay monthly membership fees	21	21: Fairness between guides is guaranteed
18	21	21: Fairness between guides is guaranteed	24	24: Donations to support the lives of ill friends who are unable to work
19	24	24: Donations to support the lives of ill friends who are unable to work	25	25: Mutual aid mechanisms are at work
20	25	25: Mutual aid mechanisms are at work	37	37: Improving the stability of life
21	22	22: Tour fee is set uniformly	26	26: Only union members can guide tourists
22	26	26: Only union members can guide tourists	27	27: Lodges Wear Vests with the Name of Guides for Union Members
23	27	27: Lodges Wear Vests with the Name of Guides for Union Members	28	28: Proof as a guide
24	29	29: Avoid excessive competition by limiting the number of guides	19	19: Responsible for guiding guests at each lodge on a rotating basis
25	31	31: Strengthening trust with lodges	32	32: 15 people obtained government certification (2017)
26	32	32: 15 people obtained government certification (2017)	33	33: Recognition of being a solid tour guide
27	33	33: Recognition of being a solid tour guide	41	41: All lodges accept unions
28	41	41: All lodges accept unions	34	34: Moves by guide self to increase reliability of guide
29	40	40: Improving the performance of tour guides	35	35: Pride of Being a Legitimate Professions
30	33	33: Recognition of being a solid tour guide	46	46: Fewer opportunities to train guides
31	8	8: Growing desire to be recognized as a proper career as a tour guide	34	34: Moves by guide self to increase reliability of guide
32	34	34: Moves by guide self to increase reliability of guide	10	____10: Movement of lodges to build good relationships with village
33	17	17: Lodge supports movement	18	18: 56 guides (all villagers) join the union
34	20	20: Revenues are evenly distributed	23	23: Union members pay monthly membership fees
35	28	28: Proof as a guide	31	31: Strengthening trust with lodges
36	35	35: Pride of Being a Legitimate Professions	36	36: Spiritual satisfaction
37	36	36: Spiritual satisfaction	39	39: Emergence and increase of female guides
38	37	37: Improving the stability of life	40	40: Improving the performance of tour guides
39	39	39: Emergence and increase of female guides	49	49: Emergence of Women's Occupation Options
40	49	49: Emergence of Women's Occupation Options	34	34: Moves by guide self to increase reliability of guide
41	42	42: Improving the quality of tourists	43	43: Increase in study tours, etc.
42	43	43: Increase in study tours, etc.	39	39: Emergence and increase of female guides
43	34	34: Moves by guide self to increase reliability of guide	44	44: Develop activities to keep nature and village in good condition
44	44	44: Develop activities to keep nature and village in good condition	45	45: Conflict with fishermen over Mbuna, a tourism resource
45	46	46: Fewer opportunities to train guides	47	47: Further improvement of tour quality is an issue
46	47	47: Further improvement of tour quality is an issue	48	48: Necessity of trying private lodging and fishing village tours

No.14 Cape Maclear Cleanup project and recycling center

link_id	from_id	from_label	to_id	to_label
1	1	1: Chembe village attracts tourists from inside and outside the country	2	2: Existence of a tourist lodge
2	2	2: Existence of a tourist lodge	3	3: Existence of tour guide association
3	3	3: Existence of tour guide association	15	15: Launched Cape Maclear Cleanup project
4	5	5: Accumulation of diverse garbage from lodges and homes	6	6: Deteriorate landscape of villages and coasts as tourism resource
5	6	6: Deteriorate landscape of villages and coasts as tourism resource	7	7: Damage to the tourism industry
6	7	7: Damage to the tourism industry	9	9: No mechanism to collect and dispose of various garbage
7	9	9: No mechanism to collect and dispose of various garbage	8	8: Activities of beach cleanup by tour guide association
8	8	8: Activities of beach cleanup by tour guide association	15	15: Launched Cape Maclear Cleanup project
9	15	15: Launched Cape Maclear Cleanup project	11	11: Collaboration and funding with all lodges
10	11	11: Collaboration and funding with all lodges	46	46: Establishment of recycling center
11	46	46: Establishment of recycling center	16	16: Employment of staff
12	16	16: Employment of staff	17	17: Garbage collection from cooperating lodges
13	17	17: Garbage collection from cooperating lodges	18	18: Large amount of garbage accumulation in the recycling center
14	18	18: Large amount of garbage accumulation in the recycling center	38	38: Insufficient segregation of lodges
15	38	38: Insufficient segregation of lodges	39	39: Sorting at the recycling center
16	39	39: Sorting at the recycling center	19	19: Foster awareness of see garbage as resource
17	19	19: Foster awareness of see garbage as resource	20	20: Collaborate with technological people in village
18	20	20: Collaborate with technological people in village	21	21: The bottle is cut and the glass etc.
19	21	21: The bottle is cut and the glass etc.	22	22: Can be an ashtray, etc.
20	20	20: Collaborate with technological people in village	23	23: Composted food waste and paper distributed in free
21	23	23: Composted food waste and paper distributed in free	25	25: Make vegetables at recycling center
22	22	22: Can be an ashtray, etc.	26	26: Commercialization through collaboration
23	25	25: Make vegetables at recycling center	26	26: Commercialization through collaboration
24	26	26: Commercialization through collaboration	27	27: Sales by shop
25	27	27: Sales by shop	28	28: Mechanism to generate profits through recycling
26	28	28: Mechanism to generate profits through recycling	100	100: Promoting the reuse of resources
27	100	100: Promoting the reuse of resources	15	15: Launched Cape Maclear Cleanup project
28	28	28: Mechanism to generate profits through recycling	30	30: Fair distribution of profits to all involved in processing and sales
29	30	30: Fair distribution of profits to all involved in processing and sales	31	31: Percentage of profit distribution stated on product label
30	31	31: Percentage of profit distribution stated on product label	32	32: Customers are reassured that revenue is distributed to villagers
31	32	32: Customers are reassured that revenue is distributed to villagers	101	101: Improved product attractiveness
32	101	101: Improved product attractiveness	20	20: Collaborate with technological people in village
33	46	46: Establishment of recycling center	33	33: Start campaign to collect garbage accumulated in village
34	33	33: Start campaign to collect garbage accumulated in village	34	34: Start fundraising on the web
35	34	34: Start fundraising on the web	35	35: Garbage collection event
36	35	35: Garbage collection event	36	36: Installation of trash cans for household garbage collection
37	36	36: Installation of trash cans for household garbage collection	37	37: Awareness of garbage changes little by little
38	37	37: Awareness of garbage changes little by little	19	19: Foster awareness of see garbage as resource
39	100	100: Promoting the reuse of resources	40	40: Processing isn't keep up accumulation speed
40	40	40: Processing isn't keep up accumulation speed	41	41: Need to speed up recycling
41	40	40: Processing isn't keep up accumulation speed	42	42: Waste reduction is a challenge
42	28	28: Mechanism to generate profits through recycling	44	44: Profits have not risen sufficiently
43	44	44: Profits have not risen sufficiently	45	45: Create sales promotion mechanism and expand sales channels

No.15 Organic farming by small-scale irrigation linked to educational activities

link_id	from_id	from_label	to_id	to_label
1	1	1: not enough number of preschool education in village	2	2: kids had challenges to perform well in primary school
2	100	100: Signs of undernourishment in preschool kids	2	2: kids had challenges to perform well in primary school
3	2	2: kids had challenges to perform well in primary school	3	3: less opportunities of adult social education
4	3	3: less opportunities of adult social education	4	4: growing needs of education invillage
5	4	4: growing needs of education invillage	7	7: launch of Sinthana project by Chembe villagers (2012～)
6	6	6: support from international donors	7	7: launch of Sinthana project by Chembe villagers (2012～)
7	40	40: model of profit-making businesses for high public values activities	7	7: launch of Sinthana project by Chembe villagers (2012～)
8	7	7: launch of Sinthana project by Chembe villagers (2012～)	8	8: Sinthana's practices of preschool and social education
9	8	8: Sinthana's practices of preschool and social education	9	9: Sinthana started supplemenrary classes of secondary school (2017～)
10	9	9: Sinthana started supplemenrary classes of secondary school (2017～)	12	12: needs of securing stable funding
11	12	12: needs of securing stable funding	13	13: developed ideas of growing cash crops by small-scale irrigation
12	13	13: developed ideas of growing cash crops by small-scale irrigation	14	14: start of small-scale irrigation by hand carrying lake water
13	14	14: start of small-scale irrigation by hand carrying lake water	15	15: start selling vegetables to tourist lodges
14	15	15: start selling vegetables to tourist lodges	16	16: started poultry farming
15	16	16: started poultry farming	17	17: developed profit-making businesses for activities of public interests
16	17	17: developed profit-making businesses for activities of public interests	18	18: produced vegetables and chickens used for nutrition of kids
17	18	18: produced vegetables and chickens used for nutrition of kids	24	24: introduce and practice organic farming for safe food of kids
18	24	24: introduce and practice organic farming for safe food of kids	19	19: nutritional conditions of kids improved
19	19	19: nutritional conditions of kids improved	20	20: groing awareness of public values preschool education in village
20	20	20: groing awareness of public values preschool education in village	21	21: better evaluation of Sinthana's activities with high public values
21	21	21: better evaluation of Sinthana's activities with high public values	22	22: match with need of tourist lodges to contribute to the community
22	22	22: match with need of tourist lodges to contribute to the community	23	23: lodges increased purchasing shinthana vegetables and chickens
23	29	29: demands of lodges for safe and high quality agricultural products	23	23: lodges increased purchasing shinthana vegetables and chickens
24	23	23: lodges increased purchasing shinthana vegetables and chickens	25	25: coaching organic agriculture to individual farmers as knowledge center
25	25	25: coaching organic agriculture to individual farmers as knowledge center	27	27: collaboration with 10 individual farmers
26	28	28: technical development and dissemination of organic farming	31	31: organic farming using chicken manure
27	28	28: technical development and dissemination of organic farming	32	32: use of local maize
28	28	28: technical development and dissemination of organic farming	33	33: use of indigenous trees as insecticides
29	31	31: organic farming using chicken manure	34	34: reputation of fresh, cheap and tasty products
30	32	32: use of local maize	34	34: reputation of fresh, cheap and tasty products
31	33	33: use of indigenous trees as insecticides	34	34: reputation of fresh, cheap and tasty products
32	34	34: reputation of fresh, cheap and tasty products	24	24: introduce and practice organic farming for safe food of kids
33	27	27: collaboration with 10 individual farmers	35	35: introduce members of farmers network to lodges
34	35	35: introduce members of farmers network to lodges	26	26: improve central roles of farmers network
35	26	26: improve central roles of farmers network	28	28: technical development and dissemination of organic farming
36	23	23: lodges increased purchasing shinthana vegetables and chickens	36	36: expanding small-scale irrigation by introducing pump-up of lake water (2017～)
37	36	36: expanding small-scale irrigation by introducing pump-up of lake water (2017～)	37	37: 80% funding covered by expanded agriculture production
38	37	37: 80% funding covered by expanded agriculture production	38	38: agriculture for preschool education supported by villagers and lodges
39	38	38: agriculture for preschool education supported by villagers and lodges	39	39: small-scale agriculture and organic farming spread in village
40	39	39: small-scale agriculture and organic farming spread in village	40	40: model of profit-making businesses for high public values activities
41	37	37: 80% funding covered by expanded agriculture production	41	41: needs of capital accumulation and scaling up
42	41	41: needs of capital accumulation and scaling up	42	42: needs of long-term sustainability
43	24	24: introduce and practice organic farming for safe food of kids	44	44: needs of strengthening scientific bases
44	44	44: needs of strengthening scientific bases	50	50: examining the possibility of introducing aquaponics
45	50	50: examining the possibility of introducing aquaponics	51	51: possibility of utilizing and selling products
46	50	50: examining the possibility of introducing aquaponics	52	52: expansion of organic farming through small-scale irrigation

No.16 Efforts by fishers to create satoumi-type fishing grounds

link_id	from_id	from_label	to_id	to_label
1	1	01: Chembe village benefit from good fishery grounds	2	02: high level of fishing activities
2	2	02: high level of fishing activities	3	03: Beach Village Committee organized in each coastal village
3	3	03: Beach Village Committee organized in each coastal village	4	04: important resource management organizations in the coast of the lake
4	4	04: important resource management organizations in the coast of the lake	5	05: BVCs organized in Chembe
5	5	05: BVCs organized in Chembe	6	06: participation of diverse coastal stakeholders in addition to fishers
6	6	06: participation of diverse coastal stakeholders in addition to fishers	7	07: activities revitalized
7	7	07: activities revitalized	12	12: awareness to deal with long distance to fishing grounds
8	12	12: awareness to deal with long distance to fishing grounds	13	13: place a chirundu near the village
9	9	09: support from USAID	8	08: artificial chirundu constructed
10	8	08: artificial chirundu constructed	11	11: failed due to long distance to and bad design of chirundu
11	11	11: failed due to long distance to and bad design of chirundu	15	15: BVC woman leader was motivated with ideas
12	15	15: BVC woman leader was motivated with ideas	13	13: place a chirundu near the village
13	13	13: place a chirundu near the village	16	16: repeated dialogue among BVC and scientists
14	16	16: repeated dialogue among BVC and scientists	17	17: new design of chirundu developed
15	16	16: repeated dialogue among BVC and scientists	14	14: make it easy to monitor and manage
16	14	14: make it easy to monitor and manage	20	20: dialogue with national park office
17	20	20: dialogue with national park office	51	51: decided chirundu site at sandy area between village and PA
18	51	51: decided chirundu site at sandy area between village and PA	22	22: construction of chirundu
19	17	17: new design of chirundu developed	18	18: to produce upwelling current by stone piles
20	18	18: to produce upwelling current by stone piles	21	21: plan to make hiding place by sinking old canoes and tree branches
21	21	21: plan to make hiding place by sinking old canoes and tree branches	22	22: construction of chirundu
22	22	22: construction of chirundu	23	23: first test trial of fishing
23	23	23: first test trial of fishing	24	24: a big catch of 5 litter Utaka with 2 big Ncheni
24	24	24: a big catch of 5 litter Utaka with 2 big Ncheni	25	25: no catch at 200m away from chirundu (June and September)
25	25	25: no catch at 200m away from chirundu (June and September)	28	28: underwater observation by diving (June and September)
26	28	28: underwater observation by diving (June and September)	105	105: confirmation of diverse fish species
27	105	105: confirmation of diverse fish species	32	32: chirundu provided hiding places and food for fish by upwelling current
28	32	32: chirundu provided hiding places and food for fish by upwelling current	33	33: new fish habitat formed around chirundu on flat sand bottom
29	33	33: new fish habitat formed around chirundu on flat sand bottom	104	104: breeding of important fisheries species confirmed
30	104	104: breeding of important fisheries species confirmed	34	34: artificial chirundu increased environmental diversity
31	34	34: artificial chirundu increased environmental diversity	35	35: provision of new fish habitat improved resource status
32	35	35: provision of new fish habitat improved resource status	36	36: Sato-Umi type effects demonstrated
33	36	36: Sato-Umi type effects demonstrated	37	37: chirundu structure was relatively stable after 5 months
34	37	37: chirundu structure was relatively stable after 5 months	38	38: provision of fish habitat with long-term stability
35	38	38: provision of fish habitat with long-term stability	39	39: success of artificial chirundu
36	39	39: success of artificial chirundu	40	40: enthusiasm of BVC members on resource enhancement
37	40	40: enthusiasm of BVC members on resource enhancement	7	07: activities revitalized
38	39	39: success of artificial chirundu	41	41: huge ripple effects in the whole Chembe village
39	41	41: huge ripple effects in the whole Chembe village	42	42: emergence of people with similar ideas
40	42	42: emergence of people with similar ideas	7	07: activities revitalized
41	22	22: construction of chirundu	43	43: subsistence fishing at chirundu by vulnerable people using wood canoes
42	43	43: subsistence fishing at chirundu by vulnerable people using wood canoes	44	44: increase resources for subsistence fishing
43	44	44: increase resources for subsistence fishing	45	45: transformation of meaning of close distance to fishing ground
44	45	45: transformation of meaning of close distance to fishing ground	13	13: place a chirundu near the village
45	51	51: decided chirundu site at sandy area between village and PA	46	46: fish migration promoted between chirundu and PA
46	46	46: fish migration promoted between chirundu and PA	100	100: OUV species settled and bred at stone piles of chirundu
47	100	100: OUV species settled and bred at stone piles of chirundu	101	101: creation of new habitats of OUV species
48	101	101: creation of new habitats of OUV species	103	103: potential rufugia created for OUV species
49	103	103: potential rufugia created for OUV species	47	47: positive impacts on PA biodiversity
50	47	47: positive impacts on PA biodiversity	34	34: artificial chirundu increased environmental diversity
51	34	34: artificial chirundu increased environmental diversity	48	48: needs to develop monitoring systems
52	34	34: artificial chirundu increased environmental diversity	49	49: needs to demonstrate upwelling currents
53	44	44: increase resources for subsistence fishing	50	50: consider ways to support subsistence fisheries and economic impacts
54	45	45: transformation of meaning of close distance to fishing ground	52	52: search for potentials of application to tourism

No.17 Cultivation and sale of pickled salad melons requiring small amount of irrigation water

link_id	from_id	from_label	to_id	to_label
1	1	1: Fertile farmland around the city of Kalapnar	2	2: Difficulty in developing water resources
2	2	2: Difficulty in developing water resources	3	3: Large-scale wheat cultivation dependent on groundwater irrigation expands
3	3	3: Large-scale wheat cultivation dependent on groundwater irrigation expands	4	4: Groundwater declines year by year
4	34	34: There are small farmers as well as large farmers	6	6: Quesmez area is unsuitable for large modern irrigated agriculture with sandy soils
5	4	4: Groundwater declines year by year	35	35: Recognition of severe groundwater depletion
6	35	35: Recognition of severe groundwater depletion	5	5: The need for agriculture that is less dependent on groundwater
7	6	6: Quesmez area is unsuitable for large modern irrigated agriculture with sandy soils	7	7: Need to grow small but high-income products
8	7	7: Need to grow small but high-income products	5	5: The need for agriculture that is less dependent on groundwater
9	5	5: The need for agriculture that is less dependent on groundwater	8	8: Focus on traditional pickle melons
10	10	10: Problem that large melons are cheap as mass-produced	11	11: Large melons require a large amount of groundwater
11	8	8: Focus on traditional pickle melons	9	9: Pickling melons can be grown in sandy soils
12	9	9: Pickling melons can be grown in sandy soils	12	12: Pickle melons irrigated on small scale and harvested early
13	11	11: Large melons require a large amount of groundwater	8	8: Focus on traditional pickle melons
14	12	12: Pickle melons irrigated on small scale and harvested early	22	22: High demand and fans
15	22	22: High demand and fans	13	13: Pickle melons are widely distributed in Turkey
16	13	13: Pickle melons are widely distributed in Turkey	14	14: Surplus can be sold on the street near the field
17	14	14: Surplus can be sold on the street near the field	16	16: Started growing pickle melons
18	16	16: Started growing pickle melons	15	15: Revenue of $20,000/ha in a short period
19	16	16: Started growing pickle melons	17	17: Export to UK through street dealer
20	17	17: Export to UK through street dealer	18	18: It turns out that there is a demand for salad melons
21	18	18: It turns out that there is a demand for salad melons	19	19: Melon farmers gather to form a farmers' union
22	19	19: Melon farmers gather to form a farmers' union	20	20: Commencement of exports to the UK through unions
23	20	20: Commencement of exports to the UK through unions	24	24: Individual farmer's production volume may be small due to union
24	24	24: Individual farmer's production volume may be small due to union	21	21: Union expands exports through multiple vendors
25	15	15: Revenue of $20,000/ha in a short period	23	23: High income even if production volume is low
26	21	21: Union expands exports through multiple vendors	23	23: High income even if production volume is low
27	23	23: High income even if production volume is low	25	25: Promotes melon production with small amounts of irrigation
28	25	25: Promotes melon production with small amounts of irrigation	36	36: Requires 1/3 of normal groundwater volume
29	36	36: Requires 1/3 of normal groundwater volume	26	26: Contributing to the conservation of groundwater
30	26	26: Contributing to the conservation of groundwater	35	35: Recognition of severe groundwater depletion
31	16	16: Started growing pickle melons	27	27: Large labor required for manual work
32	27	27: Large labor required for manual work	28	28: Collaboration between farmers is taking place
33	28	28: Collaboration between farmers is taking place	29	29: Number of melon farmers has decreased due to workload
34	29	29: Number of melon farmers has decreased due to workload	30	30: Depends on migrants from Eastern Europe
35	21	21: Union expands exports through multiple vendors	31	31: Low interest in farmers' supply chains
36	31	31: Low interest in farmers' supply chains	32	32: No attempt at processing and sales is born
37	32	32: No attempt at processing and sales is born	33	33: Necessity of branding
