# Supplementary material for: Mechanisms of synergy creation for social-ecological transformation: Leverage point analysis of the emergence of autonomous innovations
Source: PLoS One. 2025 May 14;20(5):e0323451. doi: 10.1371/journal.pone.0323451 (PMC12077674; doi:10.1371/journal.pone.0323451)
Supplement: S3 — The betweenness centrality and leverage centrality values for all nodes of causal networks are summarized and LPs are indicated for each autonomous innovation. (PDF) [file pone.0323451.s003.pdf]

No.1 Community-based marine tourism

id	label	Betweenness centrality	Bet_rank	Bet_rank_racio	Leverage Centrality			Lev.type	Node color	Node size
					Lev.all	Lev.in	Lev.out			
1	2: About 80% of villagers depend on small-scale tuna fishing	0	31.5	0.926470588	-0.333333333		0		white	1
2	4: There is no destructive fishing	0.068126521	26.5	0.779411765	0.166666667	1	0		white	4.406326034
3	6: Fish are caught but fishing grounds become far from village	0.131386861	23.5	0.691176471	0	0	0		white	7.569343066
4	1: A sea blessed with coral reefs etc. around fishing villages	0	31.5	0.926470588	-0.6		-0.333333333		white	1
5	8: Options for employment opportunities other than fishing	0.846715328	3	0.088235294	0.316666667	0.666666667	-0.333333333	in	#008000	43.33576642
6	5: Your income is never high	0.189781022	21	0.617647059	-0.166666667	0	0		white	10.48905109
7	7: Higher Education of Fishermen's Children	0	31.5	0.926470588	-0.6		0		white	1
8	9: Expansion of farmland by upstream farmers (10% of residents)	0	31.5	0.926470588	-0.333333333		0		white	1
9	23: Deterioration of coral reefs due to farmland expansion	0.063260341	28	0.823529412	0.066666667	1	0		white	4.163017032
10	26: Promotion of community based tourism	0.734793187	4	0.117647059	0.085714286	-0.333333333	0.333333333	out	#ffc0cb	37.73965937
11	19: Miguels and village youth contract and start diving tourism	0.445255474	10	0.294117647	-0.166666667	-0.333333333	0		white	23.26277372
12	46: 3 young people get dive masters	0.442822384	11	0.323529412	0	0	0		white	23.14111922
13	47: Attracting overseas customers by the website	0.440389294	12	0.352941176	0	0	0		white	23.01946472
14	14: Designation of conservation area by prefecture	0.394160584	18	0.529411765	0	0	0		white	20.7080292
15	21: Increase in tourists	0.437956204	13	0.382352941	0	0	0		white	22.89781022
16	22: Coral reef's deterioration by tourists stepping on	0.433090024	15	0.441176471	0	0	0		white	22.65450122
17	33: Growing awareness of coral conservation	0.554744526	5	0.147058824	0.2	0.333333333	0	in	#008000	28.73722628
18	37: Control of unguided tourists	0.131386861	23.5	0.691176471	-0.1	0	0		white	7.569343066
19	28: Familiarity with the sea environment around the village	0.552311436	6	0.176470588	-0.1	-0.333333333	0		white	28.61557178
20	27: Improvement and development of glass boats by ourselves	0.535279805	8	0.235294118	-0.1	0	0		white	27.76399027
21	38: Cooperation with prefectures and outsiders	0.068126521	26.5	0.779411765	0.166666667	0	1		white	4.406326034
22	32: Increased choice of means of livelihood	0.532846715	9	0.264705882	-0.166666667	0	0		white	27.64233577
23	25: Dive Masters teach how not destroy corals	0.430656934	16	0.470588235	-0.1	0	0		white	22.53284672
24	16: Sea area zoning by village ordinance	0.391727494	19	0.558823529	-0.166666667	0	-0.333333333		white	20.5863747
25	50: Promotion of conservation-type tourism	1	1	0.029411765	0.285714286	0.333333333	0.166666667	all	#0000ff	51
26	29: Tourists increase to turn down about 40% of applicants	0.435523114	14	0.411764706	0	0	0		white	22.77615572
27	45: Conservation through tourism by dividing the sea area	0.549878345	7	0.205882353	-0.166666667	0	-0.333333333		white	28.49391727
28	13: Start snorkeling and diving tourism	0.907542579	2	0.058823529	0.316666667	0.4	0.333333333	all	#0000ff	46.37712895
29	51: Growing awareness of the value of coral reefs	0.396593674	17	0.5	-0.166666667	-0.333333333	0		white	20.8296837
30	52: conservation tourism is not enough for reduce environmental impact	0.197080292	20	0.588235294	-0.166666667	-0.333333333	0		white	10.8540146
31	53: Necessity of preventing sediment inflow from upstream farmland	0.136253041	22	0.647058824	0	0	0		white	7.812652068
32	54: Collaboration with upstream farmers is a challenge	0.070559611	25	0.735294118	0.166666667	0	1		white	4.527980535
33	44: Year-round diving tourism	0	31.5	0.926470588	-0.333333333	0			white	1
34	55: Promote Environmental Conservation Agriculture for Coral Reef	0	31.5	0.926470588	-0.333333333	0			white	1

No.2 Improving the quality of cacao raw materials and high value-added distribution

id	label	Betweenness centrality	Bet_rank	Bet_rank_racio	Leverage Centrality			Lev.type	Node color	Node size
					Lev.all	Lev.in	Lev.out			
1	1: Cacao cultivation started with government support (1983)	0	40.5	0.920454545	-0.333333333		0		white	1
2	2: Expand production and increase revenue	0.056631893	31	0.704545455	0.166666667	1	0		white	3.831594635
3	3: Soil degradation by using chemical fertilizers and pesticides	0.110283159	28	0.636363636	-0.1	0	0		white	6.514157973
4	6: Old age of trees	0	40.5	0.920454545	-0.5		0		white	1
5	5: Decline in cacao production and quality deterioration	0.214605067	27	0.613636364	0.3	0.666666667	0		white	11.73025335
6	51: U Co. formation and farmers' efforts building capacity and livelihoods	0.260804769	26	0.590909091	-0.1	-0.333333333	0		white	14.04023845
7	13: Japanese chocolate maker D resonates (2012)	0.304023845	18	0.409090909	-0.214285714	0	-0.333333333		white	16.20119225
8	9: Purchase price of major vendors is same regardless of fermentation	0	40.5	0.920454545	-0.333333333		0		white	1
9	10: No fermentation makes quality deterioration	0.050670641	35	0.795454545	-0.047619048	1	-0.333333333		white	3.533532042
10	30: Major distributor B adds 900 rupiah / kg to fermented cacao	0	40.5	0.920454545	-0.6		-0.333333333		white	1
11	16: Technology development for high-quality production	1	1	0.022727273	0.428571429	0.5	0.333333333	all	#0000ff	51
12	17: Dissemination of fermenting technology	0.263785395	22	0.5	-0.214285714	-0.5	0		white	14.18926975
13	24: 2 days Anaerobic fermentation in banana peel wrapped wooden box	0.263785395	22	0.5	0	0	0		white	14.18926975
14	25: In addition, normal fermentation for 3 days	0.263785395	22	0.5	0	0	0		white	14.18926975
15	21: Increase in tourists	0.263785395	22	0.5	0	0	0		white	14.18926975
16	26: Improved flavor	0.263785395	22	0.5	0	0	0		white	14.18926975
17	27: Dry cacao for about 5 days	0.263785395	22	0.5	0	0	0		white	14.18926975
18	50: Production processing system for high-quality chocolate demand	0.263785395	22	0.5	-0.1	0	0		white	14.18926975
19	55: Providing high-quality fermented cacao	0.898658718	3.5	0.079545455	0.2	0.333333333	0	in	#008000	45.93293592
20	37: Farmers' Pride in High-Quality cacao Production Increases	0.898658718	3.5	0.079545455	-0.266666667	-0.333333333	-0.333333333		white	45.93293592
21	19: D Co. purchases fermented cacao at high price (3,000 rupiah/kg)	0.949329359	2	0.045454545	0.4	0.666666667	0.333333333	all	#0000ff	48.46646796
22	32: Provides great incentives for fermentation	0.797317437	7.5	0.170454545	-0.166666667	-0.333333333	0		white	40.86587183
23	36: Construction of value-added distribution system	0.797317437	7.5	0.170454545	0	0	0		white	40.86587183
24	33: Sell unfermented if you want quick cash	0.797317437	7.5	0.170454545	0	0	0		white	40.86587183
25	34: If you can spend time and effort, ferment and sell to D Co.	0.797317437	7.5	0.170454545	0	0	0		white	40.86587183
26	56: Strengthening cooperation with farmers through the local D Co.	0.797317437	7.5	0.170454545	-0.166666667	0	-0.5		white	40.86587183
27	35: Farmers' options expand	0.797317437	7.5	0.170454545	0.333333333	0	0.5	out	#ffc0cb	40.86587183
28	57: Increased purchase of high-quality cacao by D Co.	0.543964232	17	0.386363636	-0.380952381	0	-0.333333333		white	28.19821162
29	20: Using Compost	0.687034277	13	0.295454545	-0.214285714	-0.5	0		white	35.35171386
30	21: Technology development of ground cover by cacao leaves instead of herbicides	0.687034277	13	0.295454545	0	0	0		white	35.35171386
31	22: Developed a mechanism to prevent the pests by devising the tree arrangement	0.687034277	13	0.295454545	0	0	0		white	35.35171386
32	23: Attempts to cultivate pest-resistant strains	0.687034277	13	0.295454545	-0.1	0	-0.333333333		white	35.35171386
33	15: Control the use of chemical fertilizers and chemicals	0.687034277	13	0.295454545	0.2	0	0.333333333	out	#ffc0cb	35.35171386
34	36: Improvement of farmland management technology	0.585692996	16	0.363636364	-0.2	0	0		white	30.28464978
35	40: Pest control is difficult	0.050670641	35	0.795454545	0.066666667	0	1		white	3.533532042
36	42: Need generation change of aged farmers	0.101341282	29.5	0.670454545	-0.166666667	0	0		white	6.067064083
37	47: Create jobs for young generation in processing and distribution	0.052160954	32.5	0.738636364	0.166666667	0	1		white	3.60804769
38	60: Possibility of cacao farm tourism for D's customers	0.050670641	35	0.795454545	0	0	1		white	3.533532042
39	44: Extremely large market without requirement of fermentation	0.101341282	29.5	0.670454545	-0.166666667	-0.333333333	0		white	6.067064083
40	48: Function as a translator of U Co.	0.052160954	32.5	0.738636364	0.166666667	0	1		white	3.60804769
41	41: Need to deepen ecosystem approach	0	40.5	0.920454545	-0.333333333	0			white	1
42	43: Improving sustainability as an industry	0	40.5	0.920454545	-0.333333333	0			white	1
43	61: Working with consumers who value their cacao	0	40.5	0.920454545	-0.333333333	0			white	1
44	46: Movement to change the attitude of Company B	0	40.5	0.920454545	-0.333333333	0			white	1

No.3 Improving cacao farm management

id	label	Betweenness centrality	Bet_rank	Bet_rank_racio	Leverage Centrality			Lev.type	Node color	Node size
					Lev.all	Lev.in	Lev.out			
1	2: Growing international demand for cacao	0	48.5	0.950980392	−0.333333333		0		white	1
2	3: Improving Farmers’ Profits	0.035332786	43	0.843137255	0.166666667	1	0		white	2.766639277
3	4: Foster passive attitude to provide raw materials to major distributors	0.069022186	35	0.68627451	0	0	0		white	4.451109285
4	5: Little capital accumulation	0.1010682	32	0.62745098	−0.214285714	0	−0.333333333		white	6.053410025
5	1: Most cacao farmers are small−scale farmers of about 1 ha	0	48.5	0.950980392	−0.333333333		0		white	1
6	6: Farmers are mainly in their 40s and 50s	0.034511093	44.5	0.87254902	0.166666667	1	0		white	2.725554643
7	7: Insufficient training of young farmers	0.0673788	37.5	0.735294118	−0.1	0	0		white	4.368940016
8	10: Cacao production unstable due to climatic conditions	0	48.5	0.950980392	−0.333333333		0		white	1
9	11: Emergence of migrant workers to neighbor countries' oil palm farms	0.034511093	44.5	0.87254902	0.166666667	1	0		white	2.725554643
10	12: Farmland management during migrant work is neglected	0.0673788	37.5	0.735294118	−0.214285714	0	−0.333333333		white	4.368940016
11	13: Improving farm management and creating a system that can take pride in agriculture	0.568611339	9	0.176470588	0.085714286	0.166666667	0	in	#008000	29.43056697
12	14: U Co. starts activities with farmers as equal partners	0.566146261	10	0.196078431	−0.1	−0.333333333	0		white	29.30731306
13	15: Major distributor B and Japanese chocolate maker D collaborate in various ways	0.563681183	11	0.215686275	0	0	0		white	29.18405916
14	17: U Co.'s Mr.E appointed as coordinator of B Co.	0.561216105	12	0.235294118	−0.214285714	0	−0.333333333		white	29.06080526
15	21: Increase in tourists	0.794576828	5	0.098039216	0.428571429	0.5	0.333333333	all	#0000ff	40.72884141
16	19: Formation of farmer groups	0.688578472	6	0.117647059	−0.214285714	−0.5	0		white	35.42892358
17	20: U Co.'s Mr.C provides excellent cacao cultivation technology	0.686113394	7	0.137254902	−0.214285714	0	−0.333333333		white	35.30566968
18	21: Strengthening the network of farmer groups	1	1	0.019607843	0.476190476	0.5	0.666666667	all	#0000ff	51
19	22: Create system to convey excellent cultivation techniques and management methods to farmers.	0.961380444	2	0.039215686	−0.214285714	−0.5	0		white	49.06902219
20	23: New method developed by farmers spreads through U Co.	0.958915366	3	0.058823529	−0.166666667	0	−0.5		white	48.94576828
21	24: U Co. functions as a bidirectional translator	0.956450288	4	0.078431373	0.333333333	0	0.5	out	#ffc0cb	48.82251438
22	33: The emergence of advanced farmers 1	0.202136401	18	0.352941176	−0.166666667	0	0		white	11.10682005
23	34: Farmer A forms a farmer group with 26 neighboring farmers	0.199671323	19	0.37254902	0	0	0		white	10.98356615
24	37: Prevention of erosion by terrace−like farmland management	0.197206245	20	0.392156863	0	0	0		white	10.86031224
25	35: Obtained UTZ certification	0.194741167	21.5	0.421568627	0	0	0		white	10.73705834
26	36: Half Farmers Start Fermentation at Home	0.192276089	23.5	0.460784314	0	0	0		white	10.61380444
27	38: Management is smooth and stable	0.189811011	25.5	0.5	0	0	0		white	10.49055053
28	25: Hiring some farmers as field coordinators	0.068200493	36	0.705882353	−0.214285714	−0.5	0		white	4.410024651
29	26: Emergence of new employment opportunities	0.065735415	39	0.764705882	0	0	0		white	4.286770748
30	28: Started production of high−quality fermented cacao required by D Co.	0.486442071	13	0.254901961	−0.166666667	0	0		white	25.32210353
31	29: U Co. collection of fermented cacao from farmers	0.483976993	14	0.274509804	0	0	0		white	25.19884963
32	30: Equipment provided by D Co.	0.481511915	15	0.294117647	0	0	0		white	25.07559573
33	31: Start of cacao mass processing and export by U Co.	0.479046836	16	0.31372549	−0.1	0	−0.333333333		white	24.95234182
34	32: Increased sales options for farmers	0.476581758	17	0.333333333	0.085714286	0	0.166666667		white	24.82908792
35	45: Successful use of two sales channels, B Co. and D Co.	0.179950698	31	0.607843137	−0.166666667	0	−0.333333333		white	9.997534922
36	46: The emergence of farmers with advanced business sense	0.578471652	8	0.156862745	0.238095238	0.333333333	0.333333333	all	#0000ff	29.92358258
37	39: The emergence of advanced farmers 2	0.194741167	21.5	0.421568627	−0.166666667	0	0		white	10.73705834
38	40: Farmer A's soil management using traditional techniques	0.192276089	23.5	0.460784314	0	0	0		white	10.61380444
39	41: Controlling the use of chemical fertilizers and chemicals	0.189811011	25.5	0.5	0	0	0		white	10.49055053
40	42: Reduction of expenses	0.187345933	27.5	0.539215686	0	0	0		white	10.36729663
41	43: Expansion of farmland to 2 hectares	0.184880855	29	0.568627451	0	0	0		white	10.24404273
42	44: Ferment 30% of cacao and wholesale it to D Co.	0.182415776	30	0.588235294	0	0	0		white	10.12078882
43	47: Fostering pride and pride in being a farmer	0.187345933	27.5	0.539215686	−0.214285714	0	−0.333333333		white	10.36729663
44	27: Promote participation of youth in the agricultural sector	0.063270337	40	0.784313725	−0.214285714	0	−0.333333333		white	4.163516845
45	48: Only a small percentage of advanced farmers remain	0.072308956	33.5	0.656862745	−0.166666667	−0.333333333	0		white	4.615447823
46	49: Insufficient business mindset	0.036976171	41.5	0.81372549	0.166666667	0	1		white	2.848808546
47	53: D Co.'s sales channel is small	0.072308956	33.5	0.656862745	−0.1	0	0		white	4.615447823
48	54: The beneficiaries are limited to one part	0.036976171	41.5	0.81372549	0.166666667	0	1		white	2.848808546
49	50: Necessity of changing farmers' mindset	0	48.5	0.950980392	−0.333333333	0			white	1
50	52: Necessity of forming a farmers' union	0	48.5	0.950980392	−0.666666667	−0.5			white	1
51	55: Need to expand high value−added distribution system	0	48.5	0.950980392	−0.333333333	0			white	1

No.4 Multi-species cultivation on cacao farmland

id	label	Betweenness centrality	Bet_rank	Bet_rank_racio	Leverage Centrality			Lev.type	Node color	Node size
					Lev.all	Lev.in	Lev.out			
1	4: International increase in cacao demand and monoculture spread	0	33.5	0.930555556	−0.333333333		0		white	1
2	5: Mass injection of chemical fertilizers and chemicals becomes common	0.053097345	29	0.805555556	0.166666667	1	0		white	3.654867257
3	6: cacao harvest is twice a year and no income sometimes	0	33.5	0.930555556	−0.333333333		0		white	1
4	7: Uncertain factors such as climate and price fluctuations	0.055309735	27	0.75	0.166666667	1	0		white	3.765486726
5	44: Dry season's lack of water reduced the harvest	0.10619469	21	0.583333333	0	0	0		white	6.309734513
6	8: Management of cacao production alone is unstable	0.152654867	19	0.527777778	−0.166666667	0	−0.333333333		white	8.632743363
7	55: cacao needs shade crops	0	33.5	0.930555556	−0.333333333		0		white	1
8	15: Various crops can be grown in the forest floor	0.053097345	29	0.805555556	0.166666667	1	0		white	3.654867257
9	56: Local farmers have originally cultivated many varieties	0.101769912	23	0.638888889	−0.166666667	0	0		white	6.088495575
10	14: U Co.'s activities to increase the potential value of cacao farms	0.825221239	2	0.055555556	0.25	0.333333333	0.333333333	all	#0000ff	42.26106195
11	48: Reviewing the value of multi-cultivation	0.615044248	4	0.111111111	0.25	0.4	0	in	#008000	31.75221239
12	42: Degradation of soil and cacao bean quality	0.101769912	23	0.638888889	−0.166666667	0	0		white	6.088495575
13	49: Organic cultivation using compost	0.597345133	5	0.138888889	−0.166666667	−0.5	0		white	30.86725664
14	18: Take lemongrass and turmeric's therapeutic effects on cacao disease	0.579646018	6	0.166666667	0	0	0		white	29.98230088
15	21: Increase in tourists	0.431415929	13	0.361111111	−0.166666667	−0.333333333	0		white	22.57079646
16	22: Securing diverse sources of income	0.413716814	14	0.388888889	0	0	0		white	21.68584071
17	50: Found to lead to stable farm management	0.396017699	15	0.416666667	0	0	0		white	20.80088496
18	51: Multi-cultivation spreads to many farmers	0.378318584	16	0.444444444	0	0	0		white	19.9159292
19	24: Development of new varieties	0.360619469	17	0.472222222	0	0	0		white	19.03097345
20	26: Ideas and attempts such as raising goats on cacao farms	0.342920354	18	0.5	−0.1	0	0		white	18.1460177
21	23: Farmers devise production methods to meet their conditions	0.477876106	10	0.277777778	0.2	0.333333333	0	in	#008000	24.89380531
22	31: Income increases compared to monoculture farmers	0.460176991	11	0.305555556	−0.1	−0.333333333	0		white	24.00884956
23	40: Reduce the use of chemicals and chemical fertilizers	0.561946903	7	0.194444444	0	0	0		white	29.09734513
24	43: Cost reduction	0.544247788	8	0.222222222	0	0	0		white	28.21238938
25	30: Prevention of soil deterioration	0.526548673	9	0.25	−0.214285714	0	−0.5		white	27.32743363
26	19: Stabilization of income through multi-cultivation	0.442477876	12	0.333333333	−0.214285714	0	−0.5		white	23.12389381
27	32: Improving ecosystem functions and services in farm's environment	1	1	0.027777778	0.428571429	0.333333333	0.5	all	#0000ff	51
28	27: Increasing the resilience of farmers	0.639380531	3	0.083333333	−0.380952381	−0.333333333	−0.333333333		white	32.96902655
29	52: Extreme weather in 2017	0	33.5	0.930555556	−0.333333333		0		white	1
30	53: The harvest of many crops has drastically decreased	0.053097345	29	0.805555556	0.166666667	1	0		white	3.654867257
31	35: The Need for Adaptation to Climate Change	0.101769912	23	0.638888889	−0.1	0	0		white	6.088495575
32	41: Developing diverse products that are resilient to climate change	0.068584071	26	0.722222222	−0.047619048	−0.333333333	1		white	4.42920354
33	60: Diverse products provide attractive ingredients	0.137168142	20	0.555555556	−0.214285714	−0.333333333	0		white	7.85840708
34	61: Improving the attractiveness of cacao farmland ecosystems	0.07079646	25	0.694444444	0.166666667	0	1		white	4.539823009
35	54: Increased resilience to extreme weather events	0	33.5	0.930555556	−0.333333333	0			white	1
36	62: Possibility of promoting tourism on cacao farms	0	33.5	0.930555556	−0.333333333	0			white	1

No.5 Development of cacao farm tourism

id	label	Betweenness centrality	Bet_rank	Bet_rank_racio	Leverage Centrality			Lev.type	Node color	Node size
					Lev.all	Lev.in	Lev.out			
1	1: Polewari's main industries are agriculture and fishing	0	46	0.958333333	-0.333333333		0		white	1
2	2: Not blessed with tourism resources	0.043196544	42.5	0.885416667	0.166666667	1	0		white	3.159827214
3	3: Only a few attempts to develop tourism resources	0.084233261	38	0.791666667	0	0	0		white	5.211663067
4	5: Progress of multi-cultivation on cacao farms	0.123110151	34	0.708333333	0	0	0		white	7.155507559
5	6: Providing diverse and attractive ingredients	0.159827214	27	0.5625	-0.166666667	0	0		white	8.991360691
6	_7: Recognize possibility of tourism use of environment friendly cacao farming	0.766738661	10	0.208333333	0.333333333	0.5	0	in	#008000	39.33693305
7	8: Strength cooperation with D Co. through high-quality cacao production	0	46	0.958333333	-0.333333333		0		white	1
8	9: Improve attractiveness of D Co.'s products to consumers	0.044276458	40	0.833333333	0.166666667	1	0		white	3.213822894
9	10: Japanese consumers find value in connection D Co. and farmers	0.086393089	36	0.75	0	0	0		white	5.319654428
10	11: Grow interest in cacao farmers' production sites	0.126349892	33	0.6875	0	0	0		white	7.3174946
11	13: D Co.'s president visited Polewari in 2011	0.164146868	26	0.541666667	0	0	0		white	9.207343413
12	14: D Co.'s companion pleased the farm experience	0.199784017	19	0.395833333	-0.1	0	0		white	10.98920086
13	_12: Increased interest in cacao farm experience tourism	0.76349892	11	0.229166667	-0.266666667	-0.5	0		white	39.174946
14	15: U Co. emphasizes appeal of cacao farm experience tourism	1	1	0.020833333	0.2	0.333333333	0	in	#008000	51
15	21: Increase in tourists	0.996760259	2	0.041666667	-0.1	-0.333333333	0		white	50.83801296
16	17: D Co. plans to visit cacao farms in Japan	0.993520518	3	0.0625	-0.1	0	-0.333333333		white	50.67602592
17	44: Prefectural government also supports cacao farm tourism	0.990280778	4	0.083333333	0.2	0	0.333333333	out	#ffc0cb	50.51403888
18	18: Since 2014, Japanese consumers visited Polewari once a year	0.900647948	5	0.104166667	-0.1	0	0		white	46.03239741
19	19: Twice a year from 2016, a total of about 100 people visited	0.897408207	6	0.125	-0.1	0	-0.333333333		white	45.87041037
20	20: Mr.C's farm accepts tourists	0.894168467	7	0.145833333	0.2	0	0.333333333	out	#ffc0cb	45.70842333
21	21: Cacao farm tour	0.157667387	28	0.583333333	-0.1	0	0		white	8.88336933
22	22: Raw cacao tasting	0.154427646	29	0.604166667	0	0	0		white	8.721382289
23	23: Seedling planting	0.151187905	30	0.625	0	0	0		white	8.559395248
24	24: Experience of the fermentation process	0.147948164	31	0.645833333	0	0	0		white	8.397408207
25	25: Interaction with farmers and home cooking	0.144708423	32	0.666666667	-0.1	0	0		white	8.235421166
26	26: General consumers, students, and industry stakeholders participate	0.691144708	12	0.25	-0.1	0	0		white	35.55723542
27	27: Neighboring farmers of Mr. C also participate in acceptance	0.687904968	13	0.270833333	-0.1	0	0		white	35.39524838
28	28: Direct contact between farmers and consumers	0.86825054	8	0.166666667	0.085714286	0.333333333	-0.5	in	#008000	44.412527
29	29: Interaction with consumers stimulates farmers	0.865010799	9	0.1875	0.285714286	-0.333333333	0.5	out	#ffc0cb	44.25053996
30	30: Pride in their products	0.495680346	14	0.291666667	-0.266666667	0	-0.333333333		white	25.78401728
31	31: Strong motivation to improve the quality of farming	0.492440605	15	0.3125	0.2	0	0.333333333		white	25.62203024
32	32: Mindset change with consumers	0.359611231	16	0.333333333	-0.1	0	0		white	18.98056156
33	33: Movement to curb the use of chemicals	0.35637149	17	0.354166667	0	0	0		white	18.81857451
34	4: Expansion of environmental conservation type cacao farm management	0.353131749	18	0.375	-0.166666667	0	0		white	18.65658747
35	34: Strengthening ties between farmers and consumers	0.194384449	20	0.416666667	-0.166666667	0	0		white	10.71922246
36	35: Growing concern in cacao production overseas	0.191144708	21	0.4375	0	0	0		white	10.55723542
37	36: Change in consumer behavior	0.187904968	22	0.458333333	0	0	0		white	10.39524838
38	37: Farmers notice consumers who value their cacao	0.184665227	23	0.479166667	0	0	0		white	10.23326134
39	47: Improve farm management and production processes for consumer expectations	0.181425486	24	0.5	0	0	0		white	10.0712743
40	38: Community formation among producers and consumers by D Co.	0.178185745	25	0.520833333	-0.166666667	0	0		white	9.909287257
41	39: Build mechanism many farmers can participate is necessary	0.086393089	36	0.75	-0.166666667	0	0		white	5.319654428
42	40: Farmer guide training and possibility of farmhouse inn	0.044276458	40	0.833333333	0.166666667	0	1		white	3.213822894
43	45: Possibility to promote wide-area tourism	0.043196544	42.5	0.885416667	0.066666667	0	1		white	3.159827214
44	48: Utilization as tourism resource may improve quality of farm	0.086393089	36	0.75	-0.1	0	0		white	5.319654428
45	42: Risk of overuse as a tourism resource	0.044276458	40	0.833333333	0.166666667	0	1		white	3.213822894
46	41: Tourism may become a new livelihood option	0	46	0.958333333	-0.333333333	0			white	1
47	46: Expanding beneficiaries and participants is a challenge	0	46	0.958333333	-0.333333333	0			white	1
48	43: How tourism should not degrade the quality of farms	0	46	0.958333333	-0.333333333	0			white	1

No.6 Collaborative network construction

id	label	Betweenness centrality	Bet_rank	Bet_rank_racio	Leverage Centrality			Lev.type	Node color	Node size
					Lev.all	Lev.in	Lev.out			
1	1: Increasing charities to improve local children's nutrition（1998−)	0	34	0.971428571	−0.333333333		0		white	1
2	3: Composition of NGO (2003)	0.062921348	32	0.914285714	0.166666667	1	0		white	4.146067416
3	2: Building a network with farmers through charity activities	0.121348315	29	0.828571429	0	0	0		white	7.06741573
4	4: Expansion of activities	0.175280899	26	0.742857143	−0.1	0	0		white	9.764044944
5	11: Maintenance of irrigation canals from the Bilibili Dam (2007)	0	34	0.971428571	−0.333333333		0		white	1
6	12: Completion of secondary waterway for bilibili water (upstream)	0.065168539	31	0.885714286	0.166666667	1	0		white	4.258426966
7	13: Solving water shortages in the dry season (upstream)	0.125842697	28	0.8	0	0	0		white	7.292134831
8	15: Improving the lives of farmers	0.62247191	16	0.457142857	0.133333333	0	0.333333333		white	32.12359551
9	20: Irrigation channel management not in place (upstream)	0.182022472	25	0.714285714	0	0	0		white	10.1011236
10	50: Difficult to maintain waterways	0.233707865	20	0.571428571	−0.1	0	0		white	12.68539326
11	18: Problem of irrigated water not reaching farmers end waterway	0.568539326	17	0.485714286	0.133333333	0.333333333	0		white	29.42696629
12	19: Unfairness between upstream and downstream farmers	0.559550562	18	0.514285714	−0.2	−0.333333333	0		white	28.97752809
13	51: Creating a network to eliminate poverty in downstream areas	0.784269663	10	0.285714286	0.2	0.333333333	0	in	#008000	40.21348315
14	52: Construction of tertiary waterways utilizing networks	0.775280899	11	0.314285714	−0.1	−0.333333333	0		white	39.76404494
15	21: Increase in tourists	0.766292135	12	0.342857143	−0.1	0	0		white	39.31460674
16	28: Participation and collaboration of diverse stakeholders	1	1	0.028571429	0.2	0.333333333	0	in	#008000	51
17	29: Building a system to deliver water to downstream farmers	0.991011236	2	0.057142857	−0.1	−0.333333333	0		white	50.5505618
18	31: Strengthening water conservancy associations	0.982022472	3	0.085714286	0	0	0		white	50.1011236
19	30: Create mechanism to reflect farmers' voices on water distribution	0.973033708	4	0.114285714	0	0	0		white	49.65168539
20	34: Holding meetings with diverse stakeholders	0.964044944	5	0.142857143	0	0	0		white	49.20224719
21	53: Examination of mechanism for reaching water at the very end	0.95505618	6	0.171428571	0	0	0		white	48.75280899
22	35: Scheduling mechanism through collaboration with diverse stakeholders	0.946067416	7	0.2	0	0	0		white	48.30337079
23	36: Agreement on sluice schedule from up to downstream	0.937078652	8	0.228571429	−0.1	0	−0.333333333		white	47.85393258
24	38: Completion of manual and utilization by translator	0.928089888	9	0.257142857	0.2	0	0.333333333	out	#ffc0cb	47.40449438
25	39: Builde foundation for communication for water to reach very end	0.204494382	22	0.628571429	−0.1	0	0		white	11.2247191
26	48: Providing stakeholders with opportunities for interaction and dialogue	0.195505618	23	0.657142857	0	0	0		white	10.7752809
27	40: Distribution of irrigation water to the terminal area	0.640449438	14	0.4	0	0	0		white	33.02247191
28	41: Bifurcation is possible even in the terminal region	0.631460674	15	0.428571429	−0.1	0	−0.333333333		white	32.57303371
29	42: Still some farmers have no access to water in other end areas	0.269662921	19	0.542857143	−0.1	0	0		white	14.48314607
30	43: Need for wide−area expansion to end regions	0.208988764	21	0.6	0	0	0		white	11.4494382
31	44: In particular, the use of manuals is an important means	0.143820225	27	0.771428571	0	0	0		white	8.191011236
32	45: Utilization of manuals by excellent translators in other end regions	0.074157303	30	0.857142857	0.166666667	0	1		white	4.707865169
33	47: Collaborative network expands	0.186516854	24	0.685714286	−0.1	0	0		white	10.3258427
34	49: Open and close sluice gate mechanism where water reaches the very end first	0.649438202	13	0.371428571	−0.1	0	0		white	33.47191011
35	46: Network enhancement to facilitate dialogue	0	34	0.971428571	−0.333333333	0			white	1

No.7 Waste recycling and Tourism development

id	label	Betweenness centrality	Bet_rank	Bet_rank_racio	Leverage Centrality			Lev.type	Node color	Node size
					Lev.all	Lev.in	Lev.out			
1	1: Rice cultivation flourish at top irrigation channel	0	46	0.958333333	−0.333333333		0		white	1
2	2: Conflicts among farmers over water resources	0.046184739	39	0.8125	0.166666667	1	0		white	3.309236948
3	3: Large amount of waste in the waterway	0.090361446	37	0.770833333	0	0	0		white	5.518072289
4	4: Mr.Z collaborates NGO as water use association head	0.13253012	36	0.75	0	0	0		white	7.626506024
5	5: Build up dialogue with farmers	0.172690763	35	0.729166667	0	0	0		white	9.634538153
6	6: Recognize water shortage at downstream area by mass use in upstream	0.210843373	34	0.708333333	0	0	0		white	11.54216867
7	7: Various efforts to deliver water to downstream	0.246987952	33	0.6875	0	0	0		white	13.34939759
8	8: Scheduling for opening and closing gates	0.281124498	32	0.666666667	0	0	0		white	15.0562249
9	9: Recognize needs of treating waste hindering water flow	0.313253012	31	0.645833333	0	0	0		white	16.6626506
10	10: Successful attempt to deliver water downstream	0.343373494	20.5	0.427083333	−0.1	0	0		white	18.1686747
11	11: Mr.Z gains trust of villagers to promote actions as a village head	1	1	0.020833333	0.2	0.333333333	0	in	#008000	51
12	___12: Meeting an innovator in Malawi	0.998995984	2	0.041666667	−0.1	−0.333333333	0		white	50.9497992
13	13: Inspired by the recycling attempt of Chembe village	0.997991968	3	0.0625	−0.166666667	0	−0.5		white	50.89959839
14	14: Efforts to realize tourism village	0.996987952	4	0.083333333	0.333333333	0	0.5	out	#ffc0cb	50.84939759
15	21: Increase in tourists	0.330321285	24	0.5	−0.166666667	0	0		white	17.51606426
16	16: Demonstrate leadership in reducing waste in waterways	0.329317269	25	0.520833333	0	0	0		white	17.46586345
17	17: Focus on economic and health values of Moringa	0.328313253	26	0.541666667	0	0	0		white	17.41566265
18	18: Plant Moringa trees on banks with accumulated wastes	0.327309237	27	0.5625	0	0	0		white	17.36546185
19	19: Effective on landscape improvement and waste control	0.326305221	28	0.583333333	0	0	0		white	17.31526104
20	20: Expand tree planting to small waterways	0.325301205	29	0.604166667	0	0	0		white	17.26506024
21	21: Allocate bank plots to households to plant Moringa	0.324297189	30	0.625	−0.166666667	0	−0.333333333		white	17.21485944
22	23: Develop a park with bamboo forests for villagers to gather	0.549196787	10	0.208333333	−0.266666667	0	−0.333333333		white	28.45983936
23	24: Develop farms to grow vegetables and flowers with economic value	0.548192771	11	0.229166667	0.2	0	0.333333333	out	#ffc0cb	28.40963855
24	25: Improve landscape of residential areas through planting	0.429718876	12	0.25	−0.1	0	0		white	22.48594378
25	26: Progress in improving the landscape of the village	0.428714859	13	0.270833333	0	0	0		white	22.43574297
26	27: Set up trash cans in the village	0.427710843	14	0.291666667	0	0	0		white	22.38554217
27	28: Attempts to produce and sell crafts using waste	0.426706827	15	0.3125	0	0	0		white	22.33534137
28	29: Attempts to foster a recycling industry in the village	0.425702811	16	0.333333333	0	0	0		white	22.28514056
29	30: Production of bricks with low environmental impact using rice husk	0.424698795	17	0.354166667	0	0	0		white	22.23493976
30	31: Production of furniture by reusing building waste materials	0.423694779	18	0.375	−0.1	0	−0.333333333		white	22.18473896
31	32 : Develop activities through village under Mr.Z lead	0.422690763	19	0.395833333	0.2	0	0.333333333		white	22.13453815
32	33: Creating tourism resources by waste and landscape management	0.343373494	20.5	0.427083333	−0.1	0	0		white	18.1686747
33	49: People's awareness of the landscape has changed	0.342369478	22	0.458333333	0	0	0		white	18.1184739
34	41: Progress in landscape management by villagers themselves	0.341365462	23	0.479166667	−0.166666667	0	−0.333333333		white	18.06827309
35	22: Fostering villagers' Sence of Ownership	0.701807229	5	0.104166667	0.333333333	0.333333333	0.333333333	all	#0000ff	36.09036145
36	35: Progress in developing sustainable industries in villages	0.62248996	6	0.125	−0.166666667	−0.333333333	0		white	32.12449799
37	37: Promote cooperation with neighboring villages to plant Moringa	0.039156627	42	0.875	0	−0.333333333	1		white	2.957831325
38	36: Tourist effects such as student training tours occur	0.621485944	7	0.145833333	0	0	0		white	32.07429719
39	39: Job creation and income generation by industrial development	0.620481928	8	0.166666667	0	0	0		white	32.02409639
40	40: Moringa planting and landscape management win award	0.619477912	9	0.1875	−0.1	0	0		white	31.97389558
41	42: Trial of sustainable and environmentally friendly integrated agriculture	0.078313253	38	0.791666667	−0.1	0	0		white	4.915662651
42	43: Promotion of environmentally friendly agriculture is an issue	0.040160643	40	0.833333333	0.166666667	0	1		white	3.008032129
43	45: Big impact of International exchange of innovators	0.039156627	42	0.875	0	0	1		white	2.957831325
44	47: Needs of ideas combine waste reduction and tourism	0.039156627	42	0.875	0.066666667	0	1		white	2.957831325
45	38: Wide-area deployment covering the Moringa planting basin	0	46	0.958333333	−0.333333333	0			white	1
46	44: Important agricultural exchange with Chembe	0	46	0.958333333	−0.333333333	0			white	1
47	46: Needs of international exchange mechanism of innovators	0	46	0.958333333	−0.333333333	0			white	1
48	48: Needs of a mechanism to continuously promote new ideas	0	46	0.958333333	−0.333333333	0			white	1

No.8 Improvement of rice planting method through international exchange

id	label	Betweenness centrality	Bet_rank	Bet_rank_racio	Leverage Centrality			Lev.type	Node color	Node size
					Lev.all	Lev.in	Lev.out			
1	1 The terraced rice field landscape is UNESCO World Cultural Heritage	0	33	0.970588235	−0.333333333		0		white	1
2	2 Rice cultivation in rice terraces alone does not provide sufficient income	0.07960199	30	0.882352941	0.166666667	1	0		white	4.980099502
3	3 Labor−centered generation flows out to urban areas	0.154228856	28	0.823529412	0	0	0		white	8.711442786
4	5 Expansion of abandoned cultivated land and collapse of terraced rice fields	0.223880597	26	0.764705882	0	0	0		white	12.19402985
5	8 Crisis of agricultural continuation and impact on local safety	0.288557214	24	0.705882353	0	0	0		white	15.4278607
6	9 Negative impact on tourism industry	0.348258706	22	0.647058824	−0.1	0	0		white	18.41293532
7	24 Efforts to increase income in terraced rice fields	0.980099502	9	0.264705882	0.2	0.333333333	0	in	#008000	50.00497512
8	29 Meeting various people through international exchange projects	0.982587065	8	0.235294118	−0.1	−0.333333333	0		white	50.12935323
9	37 Emergence of attitude to incorporate external opinions	0.985074627	7	0.205882353	0	0	0		white	50.25373134
10	10 Sharing methods for efficient use of rice terraces in Japan	0.987562189	6	0.176470588	0	0	0		white	50.37810945
11	25 Proposal for change of planting interval and depth	0.990049751	5	0.147058824	0	0	0		white	50.50248756
12	26 Proposal changing amount of water in rice terraces	0.992537313	4	0.117647059	0	0	0		white	50.62686567
13	12 Determination of trial of proposal contents	0.995024876	3	0.088235294	0	0	0		white	50.75124378
14	31 Decision to trial with native species	0.997512438	2	0.058823529	−0.1	0	−0.333333333		white	50.87562189
15	21: Increase in tourists	1	1	0.029411765	0.2	0	0.333333333	out	#ffc0cb	51
16	14 Maintain the conventional method in other rice terraces	0.497512438	17	0.5	−0.1	0	0		white	25.87562189
17	15 Less impact on overall harvest	0.5	16	0.470588235	0	0	0		white	26
18	27 No recommend to poor farmers until sure of success	0.440298507	21	0.617647059	−0.1	0	0		white	23.01492537
19	28 No risk for poor farmers	0.44278607	20	0.588235294	0	0	0		white	23.13930348
20	30 No opposite, but low interest of surrounding farmers	0.445273632	19	0.558823529	0	0	0		white	23.26368159
21	16 Farmers around the area who help appear	0.447761194	18	0.529411765	−0.166666667	0	−0.333333333		white	23.3880597
22	17 Expansion of farmer network	0.955223881	10	0.294117647	0.285714286	0.333333333	0.333333333	all	#0000ff	48.76119403
23	18 trials are continuing	0.502487562	15	0.441176471	−0.1	0	−0.333333333		white	26.12437811
24	19Potential increase in rice yield and income	0.504975124	14	0.411764706	0.185714286	0	0.5		white	26.24875622
25	20 Possibility of leading to maintenance of terraced rice landscape	0.554726368	13	0.382352941	−0.166666667	−0.333333333	0		white	28.73631841
26	22 Possibility of increasing safety in the community	0.55721393	12	0.352941176	0	0	0		white	28.86069652
27	23 Potential to lead to the maintenance of tourism	0.559701493	11	0.323529412	−0.1	0	0		white	28.98507463
28	36 Improving the quality of life of individual farmers	0.335820896	23	0.676470588	−0.166666667	−0.333333333	0		white	17.79104478
29	50 Improving motivation to continue rice cultivation in rice terraces	0.278606965	25	0.735294118	0	0	0		white	14.93034826
30	51 Stop the decline in rice farmers	0.21641791	27	0.794117647	0	0	0		white	11.82089552
31	32 Activation of rice cultivation in many farmers	0.149253731	29	0.852941176	0	0	0		white	8.462686567
32	33 Reduction of abandoned cultivated land	0.077114428	31	0.911764706	0.166666667	0	1		white	4.855721393
33	35 Necessity of scientific verification of trial results	0	33	0.970588235	−0.5	0			white	1
34	52 Improvement of rice terrace management	0	33	0.970588235	−0.333333333	0			white	1

No.9 Diversification of production activities of natural rubber plantations

id	label	Betweenness centrality	Bet_rank	Bet_rank_racio	Leverage Centrality			Lev.type	Node color	Node size
					Lev.all	Lev.in	Lev.out			
1	1: Local natural rubber production expands	0	30.5	0.953125	−0.333333333		0		white	1
2	2: Needs for highly technical tappers	0.048821549	28	0.875	0.166666667	1	0		white	3.441077441
3	40: Emergence of female Tapper (mother)	0.094276094	27	0.84375	0	0	0		white	5.713804714
4	5: Production and sales of rubber seeds and seedlings by grafting	0.136363636	26	0.8125	−0.1	0	0		white	7.818181818
5	6: Accumulation of capital	0.393939394	3	0.09375	0.2	0.333333333	0	in	#008000	20.6969697
6	9: Purchase of thin land	0.388888889	4	0.125	−0.1	−0.333333333	0		white	20.44444444
7	10: Land improvement through banana production	0.383838384	5	0.15625	0	0	0		white	20.19191919
8	11: Accumulate capital in production of cucumbers, chili, etc.	0.378787879	6	0.1875	0	0	0		white	19.93939394
9	101: Development of natural rubber plantations	0.373737374	7	0.21875	0	0	0		white	19.68686869
10	39: Need support employee families' live	0.368686869	8	0.25	−0.25	0	−0.5		white	19.43434343
11	15: Unstable natural rubber production in the dry season	0	30.5	0.953125	−0.714285714		−0.5		white	1
12	102: Start efforts to stabilize income	1	1	0.03125	0.457936508	0.566666667	0.4	all	#0000ff	51
13	12: Construction for processing (smoking) facilities	0.217171717	25	0.78125	−0.35	−0.5	0		white	11.85858586
14	99: Utilization of processing (smoking) facilities	0.35016835	9	0.28125	0.122222222	−0.5	0.666666667	out	#ffc0cb	18.50841751
15	21: Increase in tourists	0.294612795	17.5	0.546875	−0.1	0	0		white	15.73063973
16	16: Durian cultivation	0.28956229	19.5	0.609375	0	0	0		white	15.47811448
17	17: Mahogany, Longkon, Ginger cultivation	0.284511785	21	0.65625	0	0	0		white	15.22558923
18	19: Mosouchiku cultivation	0.279461279	22	0.6875	0	0	0		white	14.97306397
19	20: Realization of crop diversification	0.274410774	23	0.71875	0	0	0		white	14.72053872
20	23: Secure stable income in dry season	0.269360269	24	0.75	−0.166666667	0	−0.333333333		white	14.46801347
21	27: Realization of modern farm management	0.597643098	2	0.0625	0.266666667	0.333333333	0.4	all	#0000ff	30.88215488
22	100: Business expansion such as apartment management	0.32996633	10	0.3125	−0.25	−0.5	0		white	17.4983165
23	21: Realization of diversified management	0.324915825	11	0.34375	0	0	0		white	17.24579125
24	32: Employment of surrounding micro-farmers when prices are low	0.31986532	12	0.375	0	0	0		white	16.99326599
25	30: Enhance residence and tuition support for high-skill employees	0.314814815	13	0.40625	0	0	0		white	16.74074074
26	40: The condition of the trees improves	0.30976431	14	0.4375	0	0	0		white	16.48821549
27	25: Realization of latex production in the dry season	0.304713805	15	0.46875	0	0	0		white	16.23569024
28	24: Realization of high-quality rubber production	0.2996633	16	0.5	0	0	0		white	15.98316498
29	41: No need to join a union	0.294612795	17.5	0.546875	0	0	0		white	15.73063973
30	26: Direct deal with compression plant	0.28956229	19.5	0.609375	−0.166666667	0	−0.333333333		white	15.47811448
31	37: Increase added value through end product production	0	30.5	0.953125	−0.6	−0.333333333			white	1
32	42: Reduction of environmental load by wastewater treatment	0	30.5	0.953125	−0.5	0			white	1

No.10 Reorganization and utilization of traditional salt making techniques

id	label	Betweenness centrality	Bet_rank	Bet_rank_racio	Leverage Centrality			Lev.type	Node color	Node size
					Lev.all	Lev.in	Lev.out			
1	1: Wai district, dig holes in tidal flats to collect saltwater since ancient	0	37.5	0.961538462	−0.333333333		0		white	1
2	2: Salt production by boiling down wood from coastal mangrove forests is popular	0.057453416	32	0.820512821	0.166666667	1	0		white	3.872670807
3	6: Traditional salt production gradually forgotten	0.111801242	30	0.769230769	0	0	0		white	6.590062112
4	8: Translator function by conservation organization	0.163043478	29	0.743589744	−0.214285714	0	−0.5		white	9.152173913
5	____11: Cross-generational collaborative efforts to revive traditional culture	1	1	0.025641026	0.428571429	0.333333333	0.5	all	#0000ff	51
6	9: In 2000, efforts to revive salt production in the entire village began	0.25621118	26	0.666666667	−0.214285714	−0.333333333	0		white	13.81055901
7	10: Skill acquisition by seven relative women group from village	0.260869565	25	0.641025641	0	0	0		white	14.04347826
8	12: Waste utilization, introduction of aluminum pots, ingenuity to wrap products in foil	0.26552795	24	0.615384615	0	0	0		white	14.27639752
9	13: Traditional basket with hand-knitted mangrove branches	0.270186335	23	0.58974359	0	0	0		white	14.50931677
10	14: Miniaturization to meet the needs of tourists	0.27484472	22	0.564102564	0	0	0		white	14.74223602
11	15: Use dead trees and other tree species as firewood	0.279503106	21	0.538461538	0	0	0		white	14.97515528
12	18: Young members start promoting online	0.600931677	13	0.333333333	−0.314285714	−0.333333333	0		white	31.04658385
13	19: Attract tourists and earn cash income by selling salt	0.891304348	5	0.128205128	0.2	0.333333333	0	in	#008000	45.56521739
14	16: Reduce the load on resources such as mangrove forests	0.284161491	20	0.512820513	−0.166666667	0	−0.333333333		white	15.20807453
15	21: Increase in tourists	0.895962733	3	0.076923077	−0.2	−0.333333333	−0.333333333		white	45.79813665
16	21: In 2016, the salt-making committee was reorganized under a male chairperson.	0.900621118	2	0.051282051	0.2	0	0.333333333	out	#ffc0cb	46.0310559
17	24: Cooperation of "Zongo", the decision-making body of the village	0.548136646	15	0.384615385	−0.1	0	0		white	28.4068323
18	27: In 2017, a salt factory was developed	0.552795031	14	0.358974359	−0.166666667	0	−0.333333333		white	28.63975155
19	25: Male participation and network expansion	0.894409938	4	0.102564103	0.333333333	0.333333333	0.333333333	all	#0000ff	45.72049689
20	45: New well was dug near salt factory	0.75	12	0.307692308	−0.166666667	−0.333333333	0		white	38.5
21	26: Strengthening the network of people	0.754658385	11	0.282051282	0	0	0		white	38.73291925
22	28: Committee manages visitor's admission fees and salt sales (account book)	0.75931677	10	0.256410256	0	0	0		white	38.96583851
23	29: Most proceeds allocate to activities of the committee	0.763975155	9	0.230769231	0	0	0		white	39.19875776
24	22: Collaboration with wide-area relatives such as local governments, and neighbor Kumbuna.	0.049689441	34.5	0.884615385	−0.047619048	−0.333333333	1		white	3.48447205
25	23: Enhanced collaboration with travel agencies	0.309006211	19	0.487179487	−0.1	0	0		white	16.45031056
26	36: Hotels and travel agencies listen to reputaion and approach village	0.313664596	18	0.461538462	0	0	0		white	16.68322981
27	45: Discussions in the village	0.318322981	17	0.435897436	−0.1	0	−0.333333333		white	16.91614907
28	37: Selling to tourists business, "tourism salt making business," is beginning to develop	0.322981366	16	0.41025641	0.2	0	0.333333333		white	17.14906832
29	30: Build foundation for sustainable implementation of traditional salt production begun	0.76863354	8	0.205128205	0	0	0		white	39.43167702
30	31: Revival of traditional salt production	0.773291925	7	0.179487179	0	0	0		white	39.66459627
31	32: Pride and attachment to traditional culture grow among people	0.777950311	6	0.153846154	−0.214285714	0	−0.5		white	39.89751553
32	33: Gradual increase in sales over the past 5 years	0.22826087	28	0.717948718	−0.1	0	0		white	12.41304348
33	34: The possibility of improving people's lives through the tourism industry, etc. was born	0.232919255	27	0.692307692	−0.1	0	0		white	12.64596273
34	41: Need to stably supply wages, etc. for villagers who are responsible for heavy labor	0.099378882	31	0.794871795	−0.166666667	−0.333333333	0		white	5.968944099
35	42: Women's groups involved in salt production alone cannot secure enough work time	0.051242236	33	0.846153846	0.166666667	0	1		white	3.562111801
36	40: Further increase sales and expand financial base of salt-making committee	0.049689441	34.5	0.884615385	0.066666667	0	1		white	3.48447205
37	39: Possibility of reorganizing wide-area kinship networks	0	37.5	0.961538462	−0.333333333	0			white	1
38	43: Requires equipment to increase work efficiency such as pumps and chainsaws	0	37.5	0.961538462	−0.333333333	0			white	1
39	44: Further ingenuity is needed in the method of profit sharing	0	37.5	0.961538462	−0.333333333	0			white	1

No.11 Small-scale aquaculture and multi-species cultivation

id	label	Betweenness centrality	Bet_rank	Bet_rank_racio	Leverage Centrality			Lev.type	Node color	Node size
					Lev.all	Lev.in	Lev.out			
1	1: Lack of animal protein sources and cash resources of farmers	0	45	0.9375	-0.333333333		0		white	1
2	2: Government recommends small-scale aquaculture	0.04021164	40	0.833333333	0.166666667	1	0		white	3.010582011
3	3: Slow growth of fish and no sales mechanism	0.078306878	36	0.75	0	0	0		white	4.915343915
4	4: Small-scale aquaculture does not spread	0.114285714	32	0.666666667	-0.214285714	0	0		white	6.714285714
5	5: Agricultural subjects in junior high school are substantial	0	45	0.9375	-0.333333333		0		white	1
6	6: Practice of agricultural practice	0.041269841	39	0.8125	0.166666667	1	0		white	3.063492063
7	7: Learn about multi-cultivation and aquaculture	0.08042328	35	0.729166667	0	0	0		white	5.021164021
8	8: It does not lead to practice	0.117460317	31	0.645833333	0	0	0		white	6.873015873
9	9: Persuade father based on learning in junior high school at age 14	0.152380952	26.5	0.552083333	-0.214285714	0	0		white	8.619047619
10	10: Practice of multi-cultivation and aquaculture with own ideas	0.922751323	2	0.041666667	0.428571429	0.6	0	in	#008000	47.13756614
11	11: Use abundant underground water as a water source	0.915343915	3	0.0625	-0.214285714	-0.6	0		white	46.76719577
12	12: Connect multiple aquaculture ponds in a cascade type	0.907936508	4	0.083333333	0	0	0		white	46.3968254
13	13: The top pond has water even in the dry season	0.900529101	5	0.104166667	0	0	0		white	46.02645503
14	17: Challenges in selecting fish species and cultivated crops	0.893121693	6	0.125	-0.166666667	0	-0.333333333		white	45.65608466
15	21: Increase in tourists	0	45	0.9375	-0.333333333		0		white	1
16	15: Assistance is unstable	0.039153439	41	0.854166667	0.166666667	1	0		white	2.957671958
17	16: Not getting the results I want	0.076190476	37	0.770833333	-0.166666667	0	-0.333333333		white	4.80952381
18	18: Repeat trial and error	1	1	0.020833333	0.333333333	0.333333333	0.333333333	all	#0000ff	51
19	19: Banana cultivation	0.281481481	18	0.375	-0.166666667	-0.333333333	0		white	15.07407407
20	20: Growing pineapple and avocado	0.274074074	19	0.395833333	0	0	0		white	14.7037037
21	21: Responding to changes in market demand	0.266666667	20	0.416666667	0	0	0		white	14.33333333
22	22: Trial cultivation of guava and sugarcane	0.259259259	21	0.4375	0	0	0		white	13.96296296
23	23: Successful large-scale cultivation of pineapple	0.251851852	23	0.479166667	-0.1	0	-0.333333333		white	13.59259259
24	24: Use of sugar cane to strengthen pond embankments	0.244444444	24	0.5	0.085714286	0	0.166666667		white	13.22222222
25	32: Improvement of management technology for aquaculture ponds	0.774603175	7	0.145833333	0.238095238	0.333333333	0.166666667	all	#0000ff	39.73015873
26	47: Practice of small-scale irrigation in the dry season	0.285714286	17	0.354166667	0.119047619	-0.333333333	0.666666667		white	15.28571429
27	25: Start farming locally available fish species	0.666666667	8	0.166666667	-0.166666667	-0.333333333	0		white	34.33333333
28	26: Introduction of fish species recommended by the Fisheries Bureau	0.659259259	9	0.1875	0	0	0		white	33.96296296
29	28: Establishment of aquaculture schedule	0.651851852	10	0.208333333	-0.166666667	0	-0.333333333		white	33.59259259
30	29: Sold in the dry season when the lake is less landed	0.437037037	12	0.25	-0.166666667	-0.333333333	0		white	22.85185185
31	30: Learn and utilize your own manufacturing technology	0.42962963	13	0.270833333	-0.1	0	-0.333333333		white	22.48148148
32	31: Profits from aquaculture	0.422222222	14.5	0.302083333	0.15	0	0.4		white	22.11111111
33	49: Strong awareness of economies of scale	0.106878307	33	0.6875	-0.1	0	0		white	6.343915344
34	33: Profit improvement with scale expansion	0.099470899	34	0.708333333	-0.214285714	0	-0.5		white	5.973544974
35	34: Realizing diversified management	0.505820106	11	0.229166667	0.404761905	0.333333333	0.566666667	all	#0000ff	26.29100529
36	40: Centralized production of the most profitable crops	0.152380952	26.5	0.552083333	-0.314285714	-0.333333333	0		white	8.619047619
37	41: Cultivate different crops in different seasons	0.422222222	14.5	0.302083333	0.133333333	0.333333333	0		white	22.11111111
38	42: Stable income throughout the year	0.414814815	16	0.333333333	-0.314285714	-0.333333333	0		white	21.74074074
39	35: Strengthening cooperation with neighboring farmers	0.256084656	22	0.458333333	0.05	-0.333333333	0.333333333		white	13.8042328
40	36: Building a system to learn from each other	0.157671958	25	0.520833333	-0.1	0	0		white	8.883597884
41	37: Cultivation trial of new crops	0.15026455	28	0.583333333	0	0	0		white	8.513227513
42	38: Enjoying a new attempt	0.142857143	29	0.604166667	0	0	0		white	8.142857143
43	39: Fostering pride as a farmer	0.135449735	30	0.625	-0.214285714	0	0		white	7.772486772
44	46: Organizing farmers and sharing management skills	0.045502646	38	0.791666667	0.066666667	0	1		white	3.275132275
45	44: Stabilization of management in response to changes in demand, etc.	0	45	0.9375	-0.666666667	-0.333333333			white	1
46	45: Devising sales channels for farmed fish	0	45	0.9375	-0.5	0			white	1
47	49: Strengthening collaboration with scientists	0	45	0.9375	-0.333333333	0			white	1
48	48: Expansion of irrigation scale	0	45	0.9375	-0.5	0			white	1

No.12 Seasonal fishing bans around Mbenji Island by traditional chiefs and communities

id	label	Betweenness centrality	Bet_rank	Bet_rank_racio	Leverage Centrality			Lev.type	Node color	Node size
					Lev.all	Lev.in	Lev.out			
1	1: Rich in fishing ground around the island	0	31.5	0.954545455	-0.333333333		0		white	1
2	51: Open access to fishing ground	0.069767442	27	0.818181818	0.166666667	1	0		white	4.488372093
3	3: Increace in number of access to the fishing ground	0.134883721	26	0.787878788	0	0	0		white	7.744186047
4	7: Rise of risks to the fishermen	0.195348837	25	0.757575758	0	0	0		white	10.76744186
5	17: Shift to other subsistence in the rainy season	0.251162791	24	0.727272727	-0.1	0	0		white	13.55813953
6	12: Recognition of the island as a sacred place	0	31.5	0.954545455	-0.6		-0.333333333		white	1
7	9: Practice of prohibited fishing in the rainy season	0.762790698	8	0.242424242	0.085714286	0.166666667	0	in	#008000	39.13953488
8	15: Limited fishing activities during the rainy season	0.758139535	9	0.272727273	-0.1	-0.333333333	0		white	38.90697674
9	13: Less frequency of reaching out to the island	0.753488372	10	0.303030303	0	0	0		white	38.6744186
10	16: Decreasing of fishing pressure during the rainy season	0.748837209	11	0.333333333	0	0	0		white	38.44186047
11	18: Utaka's spawning season protection in rainy season	0.744186047	12	0.363636364	-0.1	0	-0.333333333		white	38.20930233
12	19: Stabilization of utaka resources	0.739534884	13	0.393939394	0.2	0	0.333333333		white	37.97674419
13	21: Recognition of utaka resources being in good condition	0.6	14	0.424242424	-0.1	0	0		white	31
14	22: Recognition of resources' being in good condition as a result of efforts to prohibit fishing	0.595348837	15	0.454545455	0	0	0		white	30.76744186
15	21: Increase in tourists	0.590697674	16	0.484848485	0	0	0		white	30.53488372
16	24: Increasing awareness of local pride	0.586046512	17	0.515151515	-0.1	0	0		white	30.30232558
17	26: Increasing reliability of traditional leaders	1	1	0.03030303	0.133333333	0.333333333	-0.333333333	in	#008000	51
18	32: External actor`s collaboration (govenments, NGOs, etc)	0.995348837	2	0.060606061	0.133333333	-0.333333333	0.333333333	out	#ffc0cb	50.76744186
19	31: Demonstration of translator function	0.855813953	4	0.121212121	-0.1	0	0		white	43.79069767
20	33: Increasing opportunities to select and accept information and knowledge	0.851162791	5	0.151515152	0	0	0		white	43.55813953
21	10: Recongnision of the rainy season as utaka's spawning season	0.846511628	6	0.181818182	0	0	0		white	43.3255814
22	11: Prohibited fishing leads to preserve utaka's spawning season	0.841860465	7	0.212121212	-0.166666667	0	-0.333333333		white	43.09302326
23	29: Local enforcement (governing and controlling)	0.9	3	0.090909091	0.352380952	0.666666667	0.333333333	all	#0000ff	46
24	100: Disseminating the values of local resource management practices	0.06744186	28.5	0.863636364	0.066666667	0	1		white	4.372093023
25	53: Existence of advisory committee	0.381395349	18	0.545454545	-0.166666667	-0.333333333	0		white	20.06976744
26	50: A solemn ceremony and review of fishing activities	0.376744186	19	0.575757576	0	0	0		white	19.8372093
27	48: Penalties for violators	0.372093023	20	0.606060606	0	0	0		white	19.60465116
28	55: Recognition that prohibited fishing is not always appropriate	0.36744186	21	0.636363636	0	0	0		white	19.37209302
29	49: System for measuring and registering the mesh size of fishing nets	0.362790698	22	0.666666667	0	0	0		white	19.13953488
30	30: Trial of more appropriate and feasible resource management tools	0.358139535	23	0.696969697	-0.1	0	0		white	18.90697674
31	41: Practice and realization of value-added distribution	0.06744186	28.5	0.863636364	0.066666667	0	1		white	4.372093023
32	54: Impact on national policies and other communities	0	31.5	0.954545455	-0.333333333	0			white	1
33	43: Securing profits commensurate with value-added distribution	0	31.5	0.954545455	-0.333333333	0			white	1

No.13 Formation and operation of a tour guide association by local residents

id	label	Betweenness centrality	Bet_rank	Bet_rank_racio	Leverage Centrality			Lev.type	Node color	Node size
					Lev.all	Lev.in	Lev.out			
1	1: Chembe village in a tourist area	0	43.5	0.966666667	−0.333333333		0		white	1
2	2: There are many tourist lodges	0.040598291	40	0.888888889	0.166666667	1	0		white	3.02991453
3	3: Increase in tourists	0.079059829	36	0.8	0	0	0		white	4.952991453
4	4: Emergence of souvenir sellers and self-proclaimed tour guides	0.115384615	33	0.733333333	0	0	0		white	6.769230769
5	5: A villager's guide to theft damage was suspected	0.14957265	31	0.688888889	0	0	0		white	8.478632479
6	7: Occurrence of feuds with lodges	0.181623932	30	0.666666667	0	0	0		white	10.08119658
7	___10: Movement of lodges to build good relationships with village	0.907051282	2	0.044444444	−0.214285714	−0.5	0		white	46.3525641
8	12: Guided discussion	0.89957265	3	0.066666667	0	0	0		white	45.97863248
9	13: Three young people formed a tour guide association	0.892094017	4	0.088888889	0	0	0		white	45.60470085
10	14: Malawi entry visa price goes up	0	43.5	0.966666667	−0.333333333		0		white	1
11	15: Fewer tourists from abroad	0.038461538	41	0.911111111	0.166666667	1	0		white	2.923076923
12	16: Domestic tourists mainstream	0.074786325	37	0.822222222	0	0	0		white	4.739316239
13	18: 56 guides (all villagers) join the union	0.877136752	6	0.133333333	0.2	0	0.333333333	out	#ffc0cb	44.85683761
14	19: Responsible for guiding guests at each lodge on a rotating basis	0.418803419	11	0.244444444	0	0	0		white	21.94017094
15	21: Increase in tourists	0.433760684	9	0.2	−0.1	0	0		white	22.68803419
16	23: Union members pay monthly membership fees	0.403846154	13	0.288888889	0	0	0		white	21.19230769
17	21: Fairness between guides is guaranteed	0.396367521	14	0.311111111	0	0	0		white	20.81837607
18	24: Donations to support the lives of ill friends who are unable to work	0.388888889	16	0.355555556	0	0	0		white	20.44444444
19	25: Mutual aid mechanisms are at work	0.381410256	18	0.4	0	0	0		white	20.07051282
20	22: Tour fee is set uniformly	0.394230769	15	0.333333333	−0.1	0	0		white	20.71153846
21	26: Only union members can guide tourists	0.386752137	17	0.377777778	0	0	0		white	20.33760684
22	27: Lodges Wear Vests with the Name of Guides for Union Members	0.379273504	19	0.422222222	0	0	0		white	19.96367521
23	29: Avoid excessive competition by limiting the number of guides	0.426282051	10	0.222222222	0	0	0		white	22.31410256
24	31: Strengthening trust with lodges	0.364316239	23	0.511111111	0	0	0		white	19.21581197
25	32: 15 people obtained government certification (2017)	0.356837607	25	0.555555556	−0.1	0	−0.333333333		white	18.84188034
26	33: Recognition of being a solid tour guide	0.349358974	27	0.6	0.2	0	0.333333333		white	18.46794872
27	41: All lodges accept unions	0.213675214	28	0.622222222	−0.314285714	0	−0.333333333		white	11.68376068
28	40: Improving the performance of tour guides	0.366452991	22	0.488888889	0	0	0		white	19.32264957
29	8: Growing desire to be recognized as a proper career as a tour guide	0.211538462	29	0.644444444	−0.214285714	0	−0.333333333		white	11.57692308
30	34: Moves by guide self to increase reliability of guide	1	1	0.022222222	0.428571429	0.5	0.333333333	all	#0000ff	51
31	17: Lodge supports movement	0.884615385	5	0.111111111	−0.1	0	−0.333333333		white	45.23076923
32	20: Revenues are evenly distributed	0.411324786	12	0.266666667	0	0	0		white	21.56623932
33	28: Proof as a guide	0.371794872	21	0.466666667	0	0	0		white	19.58974359
34	35: Pride of Being a Legitimate Professions	0.358974359	24	0.533333333	0	0	0		white	18.94871795
35	36: Spiritual satisfaction	0.351495726	26	0.577777778	−0.1	0	0		white	18.57478632
36	37: Improving the stability of life	0.373931624	20	0.444444444	0	0	0		white	19.6965812
37	39: Emergence and increase of female guides	0.520299145	7	0.155555556	0.2	0.333333333	0	in	#008000	27.01495726
38	49: Emergence of Women's Occupation Options	0.512820513	8	0.177777778	−0.314285714	−0.333333333	−0.333333333		white	26.64102564
39	42: Improving the quality of tourists	0.108974359	34	0.755555556	0	0	0		white	6.448717949
40	43: Increase in study tours, etc.	0.141025641	32	0.711111111	−0.1	0	0		white	8.051282051
41	44: Develop activities to keep nature and village in good condition	0.042735043	39	0.866666667	−0.047619048	−0.5	1		white	3.136752137
42	46: Fewer opportunities to train guides	0.085470085	35	0.777777778	−0.1	0	0		white	5.273504274
43	47: Further improvement of tour quality is an issue	0.043803419	38	0.844444444	0.166666667	0	1		white	3.19017094
44	45: Conflict with fishermen over Mbuna, a tourism resource	0	43.5	0.966666667	−0.333333333	0			white	1
45	48: Necessity of trying private lodging and fishing village tours	0	43.5	0.966666667	−0.333333333	0			white	1

No.14 Cape Maclear Cleanup project and recycling center

id	label	Betweenness centrality	Bet_rank	Bet_rank_racio	Leverage Centrality			Lev.type	Node color	Node size
					Lev.all	Lev.in	Lev.out			
1	1: Chembe village attracts tourists from inside and outside the country	0	38	0.95	−0.333333333		0		white	1
2	2: Existence of a tourist lodge	0.051401869	35	0.875	0.166666667	1	0		white	3.570093458
3	3: Existence of tour guide association	0.099688474	32	0.8	−0.166666667	0	0		white	5.984423676
4	5: Accumulation of diverse garbage from lodges and homes	0	38	0.95	−0.333333333		0		white	1
5	6: Deteriorate landscape of villages and coasts as tourism resource	0.054517134	33.5	0.8375	0.166666667	1	0		white	3.725856698
6	7: Damage to the tourism industry	0.105919003	31	0.775	0	0	0		white	6.295950156
7	9: No mechanism to collect and dispose of various garbage	0.154205607	29	0.725	0	0	0		white	8.710280374
8	8: Activities of beach cleanup by tour guide association	0.199376947	24.5	0.6125	−0.166666667	0	0		white	10.96884735
9	15: Launched Cape Maclear Cleanup project	0.831775701	5	0.125	0.285714286	0.5	0	in	#008000	42.58878505
10	11: Collaboration and funding with all lodges	0.827102804	6	0.15	−0.266666667	−0.5	−0.333333333		white	42.35514019
11	46: Establishment of recycling center	0.822429907	7	0.175	0.2	0	0.333333333	out	#ffc0cb	42.12149533
12	16: Employment of staff	0.382398754	14.5	0.3625	−0.1	0	0		white	20.11993769
13	17: Garbage collection from cooperating lodges	0.377725857	16.5	0.4125	0	0	0		white	19.88629283
14	18: Large amount of garbage accumulation in the recycling center	0.37305296	18.5	0.4625	0	0	0		white	19.65264798
15	21: Increase in tourists	0.368380062	20.5	0.5125	0	0	0		white	19.41900312
16	39: Sorting at the recycling center	0.363707165	22.5	0.5625	−0.1	0	0		white	19.18535826
17	19: Foster awareness of see garbage as resource	0.771028037	8	0.2	0.085714286	0.333333333	−0.333333333	in	#008000	39.55140187
18	20: Collaborate with technological people in village	1	1	0.025	0.285714286	0.166666667	0.333333333	all	#0000ff	51
19	21: The bottle is cut and the glass etc.	0.471183801	10.5	0.2625	−0.166666667	−0.333333333	0		white	24.55919003
20	23: Composted food waste and paper distributed in free	0.471183801	10.5	0.2625	−0.166666667	−0.333333333	0		white	24.55919003
21	22: Can be an ashtray, etc.	0.466510903	12.5	0.3125	−0.1	0	0		white	24.32554517
22	25: Make vegetables at recycling center	0.466510903	12.5	0.3125	−0.1	0	0		white	24.32554517
23	26: Commercialization through collaboration	0.976635514	2	0.05	0.2	0.333333333	0	in	#008000	49.8317757
24	27: Sales by shop	0.971962617	3	0.075	−0.266666667	−0.333333333	−0.5		white	49.59813084
25	28: Mechanism to generate profits through recycling	0.96728972	4	0.1	0.285714286	0	0.4	out	#ffc0cb	49.36448598
26	100: Promoting the reuse of resources	0.601246106	9	0.225	−0.095238095	0	0.166666667		white	31.0623053
27	30: Fair distribution of profits to all involved in processing and sales	0.199376947	24.5	0.6125	−0.166666667	0	0		white	10.96884735
28	31: Percentage of profit distribution stated on product label	0.19470405	26	0.65	0	0	0		white	10.73520249
29	32: Customers are reassured that revenue is distributed to villagers	0.190031153	27	0.675	0	0	0		white	10.50155763
30	101: Improved product attractiveness	0.185358255	28	0.7	−0.166666667	0	−0.333333333		white	10.26791277
31	33: Start campaign to collect garbage accumulated in village	0.382398754	14.5	0.3625	−0.1	0	0		white	20.11993769
32	34: Start fundraising on the web	0.377725857	16.5	0.4125	0	0	0		white	19.88629283
33	35: Garbage collection event	0.37305296	18.5	0.4625	0	0	0		white	19.65264798
34	36: Installation of trash cans for household garbage collection	0.368380062	20.5	0.5125	0	0	0		white	19.41900312
35	37: Awareness of garbage changes little by little	0.363707165	22.5	0.5625	−0.1	0	0		white	19.18535826
36	40: Processing isn't keep up accumulation speed	0.109034268	30	0.75	0.333333333	0	1		white	6.451713396
37	44: Profits have not risen sufficiently	0.054517134	33.5	0.8375	0	0	1		white	3.725856698
38	41: Need to speed up recycling	0	38	0.95	−0.5	0			white	1
39	42: Waste reduction is a challenge	0	38	0.95	−0.5	0			white	1
40	45: Create sales promotion mechanism and expand sales channels	0	38	0.95	−0.333333333	0			white	1

No.15 Organic farming by small-scale irrigation linked to educational activities

id	label	Betweenness centrality	Bet_rank	Bet_rank_racio	Leverage Centrality			Lev.type	Node color	Node size
					Lev.all	Lev.in	Lev.out			
1	1: not enough number of preschool education in village	0	40	0.930232558	−0.5		0		white	1
2	100: Signs of undernourishment in preschool kids	0	40	0.930232558	−0.5		0		white	1
3	2: kids had challenges to perform well in primary school	0.088167053	31.5	0.73255814	0.4	1	0		white	5.408352668
4	3: less opportunities of adult social education	0.128770302	29.5	0.686046512	−0.1	−0.333333333	0		white	7.438515081
5	4: growing needs of education invillage	0.167053364	28	0.651162791	−0.166666667	0	0		white	9.352668213
6	6: support from international donors	0	40	0.930232558	−0.6		0		white	1
7	40: model of profit-making businesses for high public values activities	0.383990719	21	0.488372093	−0.166666667	0	0		white	20.19953596
8	7: launch of Sinthana project by Chembe villagers (2012〜)	0.633410673	7	0.162790698	0.4	0.666666667	0	in	#008000	32.67053364
9	8: Sinthana's practices of preschool and social education	0.63225058	8	0.186046512	−0.166666667	−0.5	0		white	32.612529
10	9: Sinthana started supplemenrary classes of secondary school (2017〜)	0.631090487	9	0.209302326	0	0	0		white	32.55452436
11	12: needs of securing stable funding	0.629930394	10	0.23255814	0	0	0		white	32.49651972
12	13: developed ideas of growing cash crops by small-scale irrigation	0.628770302	11	0.255813953	0	0	0		white	32.43851508
13	14: start of small-scale irrigation by hand carrying lake water	0.627610209	12	0.279069767	0	0	0		white	32.38051044
14	15: start selling vegetables to tourist lodges	0.626450116	13	0.302325581	0	0	0		white	32.3225058
15	21: Increase in tourists	0.625290023	14	0.325581395	0	0	0		white	32.26450116
16	17: developed profit-making businesses for activities of public interests	0.62412993	15	0.348837209	0	0	0		white	32.20649652
17	18: produced vegetables and chickens used for nutrition of kids	0.622969838	16	0.372093023	−0.166666667	0	−0.333333333		white	32.14849188
18	24: introduce and practice organic farming for safe food of kids	1	1	0.023255814	0.25	0.066666667	0.333333333	all	#0000ff	51
19	19: nutritional conditions of kids improved	0.827146172	3	0.069767442	−0.166666667	−0.333333333	0		white	42.35730858
20	20: groing awareness of public values preschool education in village	0.825986079	4	0.093023256	0	0	0		white	42.29930394
21	21: better evaluation of Sinthana's activities with high public values	0.824825986	5	0.11627907	0	0	0		white	42.2412993
22	22: match with need of tourist lodges to contribute to the community	0.823665893	6	0.139534884	−0.166666667	0	−0.333333333		white	42.18329466
23	29: demands of lodges for safe and high quality agricultural products	0	40	0.930232558	−0.6		−0.333333333		white	1
24	23: lodges increased purchasing shinthana vegetables and chickens	0.864269142	2	0.046511628	0.4	0.666666667	0.333333333	all	#0000ff	44.21345708
25	25: coaching organic agriculture to individual farmers as knowledge center	0.346867749	22	0.511627907	−0.166666667	−0.333333333	0		white	18.34338747
26	28: technical development and dissemination of organic farming	0.342227378	26	0.604651163	0.333333333	0	0.5		white	18.11136891
27	31: organic farming using chicken manure	0.085846868	34	0.790697674	−0.333333333	0	0		white	5.292343387
28	32: use of local maize	0.085846868	34	0.790697674	−0.333333333	0	0		white	5.292343387
29	33: use of indigenous trees as insecticides	0.085846868	34	0.790697674	−0.333333333	0	0		white	5.292343387
30	34: reputation of fresh, cheap and tasty products	0.337587007	27	0.627906977	0.25	0.5	−0.333333333		white	17.87935035
31	27: collaboration with 10 individual farmers	0.345707657	23	0.534883721	0	0	0		white	18.28538283
32	35: introduce members of farmers network to lodges	0.344547564	24	0.558139535	0	0	0		white	18.22737819
33	26: improve central roles of farmers network	0.343387471	25	0.581395349	−0.166666667	0	−0.5		white	18.16937355
34	36: expanding small-scale irrigation by introducing pump-up of lake water (2017〜)	0.474477958	17	0.395348837	−0.266666667	−0.333333333	−0.333333333		white	24.72389791
35	37: 80% funding covered by expanded agriculture production	0.473317865	18	0.418604651	0.2	0	0.333333333		white	24.66589327
36	38: agriculture for preschool education supported by villagers and lodges	0.386310905	19	0.441860465	−0.1	0	0		white	20.31554524
37	39: small-scale agriculture and organic farming spread in village	0.385150812	20	0.465116279	0	0	0		white	20.2575406
38	41: needs of capital accumulation and scaling up	0.042923434	36	0.837209302	0.066666667	0	1		white	3.146171694
39	44: needs of strengthening scientific bases	0.128770302	29.5	0.686046512	−0.266666667	−0.333333333	−0.333333333		white	7.438515081
40	50: examining the possibility of introducing aquaponics	0.088167053	31.5	0.73255814	0.4	0	1		white	5.408352668
41	42: needs of long-term sustainability	0	40	0.930232558	−0.333333333	0			white	1
42	51: possibility of utilizing and selling products	0	40	0.930232558	−0.5	0			white	1
43	52: expansion of organic farming through small-scale irrigation	0	40	0.930232558	−0.5	0			white	1

No.16 Efforts by fishers to create satoumi-type fishing grounds

id	label	Betweenness centrality	Bet_rank	Bet_rank_racio	Leverage Centrality			Lev.type	Node color	Node size
					Lev.all	Lev.in	Lev.out			
1	01: Chembe village benefit from good fishery grounds	0	47.5	0.95	−0.333333333		0		white	1
2	02: high level of fishing activities	0.036943745	41.5	0.83	0.166666667	1	0		white	2.847187238
3	03: Beach Village Committee organized in each coastal village	0.072208228	39	0.78	0	0	0		white	4.610411419
4	04: important resource management organizations in the coast of the lake	0.105793451	37	0.74	0	0	0		white	6.289672544
5	05: BVCs organized in Chembe	0.137699412	36	0.72	0	0	0		white	7.884970613
6	06: participation of diverse coastal stakeholders in addition to fishers	0.167926113	34	0.68	−0.166666667	0	0		white	9.396305626
7	07: activities revitalized	0.729638959	3	0.06	0.333333333	0.5	0	in	#008000	37.48194794
8	12: awareness to deal with long distance to fishing grounds	0.724601175	4	0.08	−0.333333333	−0.5	0		white	37.23005877
9	09: support from USAID	0	47.5	0.95	−0.333333333		0		white	1
10	08: artificial chirundu constructed	0.035264484	43	0.86	0.166666667	1	0		white	2.763224181
11	11: failed due to long distance to and bad design of chirundu	0.068849706	40	0.8	0	0	0		white	4.442485306
12	15: BVC woman leader was motivated with ideas	0.100755668	38	0.76	−0.166666667	0	0		white	6.037783375
13	13: place a chirundu near the village	1	1	0.02	0.238095238	0.5	−0.333333333	in	#008000	51
14	16: repeated dialogue among BVC and scientists	0.994962217	2	0.04	0.085714286	−0.5	0.333333333	out	#ffc0cb	50.74811083
15	21: Increase in tourists	0.669185558	5	0.1	−0.1	0	0		white	34.45927792
16	20: dialogue with national park office	0.664147775	6	0.12	−0.1	0	−0.333333333		white	34.20738875
17	51: decided chirundu site at sandy area between village and PA	0.659109992	7	0.14	0.085714286	0	0.166666667	out	#ffc0cb	33.95549958
18	17: new design of chirundu developed	0.2829555	22	0.44	−0.1	0	0		white	15.14777498
19	18: to produce upwelling current by stone piles	0.277917716	24	0.48	0	0	0		white	14.89588581
20	21: plan to make hiding place by sinking old canoes and tree branches	0.272879933	26	0.52	−0.166666667	0	−0.333333333		white	14.64399664
21	22: construction of chirundu	0.528127624	14	0.28	0.285714286	0.333333333	0.333333333	all	#0000ff	27.40638119
22	23: first test trial of fishing	0.284634761	21	0.42	−0.166666667	−0.333333333	0		white	15.23173804
23	24: a big catch of 5 litter Utaka with 2 big Ncheni	0.279596977	23	0.46	0	0	0		white	14.97984887
24	25: no catch at 200m away from chirundu (June and September)	0.274559194	25	0.5	0	0	0		white	14.7279597
25	28: underwater observation by diving (June and September)	0.269521411	27	0.54	0	0	0		white	14.47607053
26	105: confirmation of diverse fish species	0.264483627	28	0.56	0	0	0		white	14.22418136
27	32: chirundu provided hiding places and food for fish by upwelling current	0.259445844	29	0.58	0	0	0		white	13.97229219
28	33: new fish habitat formed around chirundu on flat sand bottom	0.25440806	30	0.6	0	0	0		white	13.72040302
29	104: breeding of important fisheries species confirmed	0.249370277	31	0.62	−0.214285714	0	−0.5		white	13.46851385
30	34: artificial chirundu increased environmental diversity	0.650713686	8	0.16	0.523809524	0.333333333	0.833333333	all	#0000ff	33.5356843
31	35: provision of new fish habitat improved resource status	0.568429891	9	0.18	−0.214285714	−0.333333333	0		white	29.42149454
32	36: Sato-Umi type effects demonstrated	0.563392107	10	0.2	0	0	0		white	29.16960537
33	37: chirundu structure was relatively stable after 5 months	0.558354324	11	0.22	0	0	0		white	28.9177162
34	38: provision of fish habitat with long-term stability	0.553316541	12	0.24	−0.1	0	−0.333333333		white	28.66582704
35	39: success of artificial chirundu	0.548278757	13	0.26	0.2	0	0.333333333	out	#ffc0cb	28.41393787
36	40: enthusiasm of BVC members on resource enhancement	0.468513854	15	0.3	−0.266666667	0	0		white	24.4256927
37	41: huge ripple effects in the whole Chembe village	0.036943745	41.5	0.83	−0.1	0	0		white	2.847187238
38	42: emergence of people with similar ideas	0.031905961	44	0.88	−0.166666667	0	0		white	2.595298069
39	43: subsistence fishing at chirundu by vulnerable people using wood canoes	0.200671704	32	0.64	−0.266666667	−0.333333333	−0.333333333		white	11.03358522
40	44: increase resources for subsistence fishing	0.195633921	33	0.66	0.233333333	0	0.5		white	10.78169605
41	45: transformation of meaning of close distance to fishing ground	0.151973132	35	0.7	0.119047619	0	0.666666667		white	8.598656591
42	46: fish migration promoted between chirundu and PA	0.393786734	16	0.32	−0.1	0	0		white	20.68933669
43	100: OUV species settled and bred at stone piles of chirundu	0.38874895	17	0.34	0	0	0		white	20.43744752
44	101: creation of new habitats of OUV species	0.383711167	18	0.36	0	0	0		white	20.18555835
45	103: potential rufugia created for OUV species	0.378673384	19	0.38	0	0	0		white	19.93366919
46	47: positive impacts on PA biodiversity	0.3736356	20	0.4	−0.214285714	0	−0.5		white	19.68178002
47	48: needs to develop monitoring systems	0	47.5	0.95	−0.666666667	−0.333333333			white	1
48	49: needs to demonstrate upwelling currents	0	47.5	0.95	−0.666666667	−0.333333333			white	1
49	50: consider ways to support subsistence fisheries and economic impacts	0	47.5	0.95	−0.5	0			white	1
50	52: search for potentials of application to tourism	0	47.5	0.95	−0.5	0			white	1

No.17 Cultivation and sale of pickled salad melons requiring small amount of irrigation water

id	label	Betweenness centrality	Bet_rank	Bet_rank_racio	Leverage Centrality			Lev.type	Node color	Node size
					Lev.all	Lev.in	Lev.out			
1	1: Fertile farmland around the city of Kalapnar	0	34	0.944444444	−0.333333333		0		white	1
2	2: Difficulty in developing water resources	0.061310782	29	0.805555556	0.166666667	1	0		white	4.065539112
3	3: Large-scale wheat cultivation dependent on groundwater irrigation expands	0.118393235	25	0.694444444	0	0	0		white	6.919661734
4	34: There are small farmers as well as large farmers	0	34	0.944444444	−0.333333333		0		white	1
5	4: Groundwater declines year by year	0.171247357	22	0.611111111	−0.1	0	0		white	9.562367865
6	35: Recognition of severe groundwater depletion	0.723044397	9	0.25	0.133333333	0.333333333	0	in	#008000	37.15221987
7	6: Quesmez area is unsuitable for large modern irrigated agriculture with sandy soils	0.059196617	30	0.833333333	0.166666667	1	0		white	3.959830867
8	7: Need to grow small but high-income products	0.114164905	26	0.722222222	−0.1	0	0		white	6.708245243
9	5: The need for agriculture that is less dependent on groundwater	0.890063425	8	0.222222222	0.066666667	0.166666667	0	in	#008000	45.50317125
10	10: Problem that large melons are cheap as mass-produced	0	34	0.944444444	−0.333333333		0		white	1
11	8: Focus on traditional pickle melons	1	1	0.027777778	0.133333333	0.166666667	0	in	#008000	51
12	9: Pickling melons can be grown in sandy soils	0.99577167	2	0.055555556	−0.1	−0.333333333	0		white	50.78858351
13	11: Large melons require a large amount of groundwater	0.057082452	31	0.861111111	0.066666667	1	0		white	3.854122622
14	12: Pickle melons irrigated on small scale and harvested early	0.99154334	3	0.083333333	0	0	0		white	50.57716702
15	21: Increase in tourists	0.987315011	4	0.111111111	0	0	0		white	50.36575053
16	13: Pickle melons are widely distributed in Turkey	0.983086681	5	0.138888889	0	0	0		white	50.15433404
17	14: Surplus can be sold on the street near the field	0.978858351	6	0.166666667	−0.166666667	0	−0.5		white	49.94291755
18	16: Started growing pickle melons	0.974630021	7	0.194444444	0.333333333	0	0.5	out	#ffc0cb	49.73150106
19	17: Export to UK through street dealer	0.410147992	14	0.388888889	−0.166666667	0	0		white	21.50739958
20	18: It turns out that there is a demand for salad melons	0.405919662	15	0.416666667	0	0	0		white	21.29598309
21	19: Melon farmers gather to form a farmers' union	0.401691332	16	0.444444444	0	0	0		white	21.0845666
22	20: Commencement of exports to the UK through unions	0.397463002	17	0.472222222	0	0	0		white	20.87315011
23	24: Individual farmer's production volume may be small due to union	0.393234672	18	0.5	−0.1	0	−0.333333333		white	20.66173362
24	15: Revenue of $20,000/ha in a short period	0.255813953	20	0.555555556	−0.266666667	0	0		white	13.79069767
25	21: Union expands exports through multiple vendors	0.389006342	19	0.527777778	0.133333333	0	0.333333333		white	20.45031712
26	23: High income even if production volume is low	0.511627907	10	0.277777778	0.133333333	0.333333333	0	in	#008000	26.58139535
27	25: Promotes melon production with small amounts of irrigation	0.507399577	11	0.305555556	−0.1	−0.333333333	0		white	26.36997886
28	36: Requires 1/3 of normal groundwater volume	0.503171247	12	0.333333333	0	0	0		white	26.15856237
29	26: Contributing to the conservation of groundwater	0.498942918	13	0.361111111	−0.1	0	0		white	25.94714588
30	27: Large labor required for manual work	0.183932347	21	0.583333333	−0.166666667	0	0		white	10.19661734
31	28: Collaboration between farmers is taking place	0.126849894	23	0.638888889	0	0	0		white	7.342494715
32	29: Number of melon farmers has decreased due to workload	0.065539112	27	0.75	0.166666667	0	1		white	4.276955603
33	31: Low interest in farmers' supply chains	0.122621564	24	0.666666667	−0.1	0	0		white	7.131078224
34	32: No attempt at processing and sales is born	0.063424947	28	0.777777778	0.166666667	0	1		white	4.171247357
35	30: Depends on migrants from Eastern Europe	0	34	0.944444444	−0.333333333	0			white	1
36	33: Necessity of branding	0	34	0.944444444	−0.333333333	0			white	1
