# Supplementary material for: Mechanisms of synergy creation for social-ecological transformation: Leverage point analysis of the emergence of autonomous innovations
Source: PLoS One. 2025 May 14;20(5):e0323451. doi: 10.1371/journal.pone.0323451 (PMC12077674; doi:10.1371/journal.pone.0323451)
Supplement: S4 — The data for analysis to draw Fig 3 is represented. (PDF) [file pone.0323451.s004.pdf]

synergistic(10)	single(7)
3	3
4	1
6	1
3	2
6	3
4	2
2	5
4	
6	
3	
