# Supplementary material for: Mechanisms of synergy creation for social-ecological transformation: Leverage point analysis of the emergence of autonomous innovations
Source: PLoS One. 2025 May 14;20(5):e0323451. doi: 10.1371/journal.pone.0323451 (PMC12077674; doi:10.1371/journal.pone.0323451)
Supplement: Attachment — Submitted filename: Response to Reviewers.docx [file pone.0323451.s006.docx]

Ref. No.: PONE-D-24-53688

Title: Mechanisms of synergy creation for social-ecological transformation: Leverage point analysis of the emergence of autonomous innovations

PLOS ONE

Dear Editor,

Thank you for the opportunity to revise and resubmit our manuscript to PLOS ONE. We are grateful to the two reviewers whose suggestions have allowed us to significantly improve the article.

We made a major revision according to the comments by the reviewers. Below, please find a point-by-point response to each of the referees' comments, organized in a table for clarity.

| **Reviewer #1:** | **Response** |
| --- | --- |
| Thank you for giving me the possibility to read and evaluate your work. The article covers an interesting and extremely timely topic, exploring the social and ecological transformation of systems and related challenges related to today's uncertainty, that is coherent with the focus of Plos One journal.  Therefore, the manuscript covers an extremely up-to-date and interesting topic. Despite the interest in the topic, I believe the article can be further enriched, and I hope my advice and suggestions can help the author(s) in strengthening their manuscript. | Thank you for the constructive, positive, and stimulating comments on our paper. |
| - Introduction:  The introduction sections in my opinion are too fragmented and reduce the readability of the article. I suggest the authors keep section 1, i.e., introduction, but without providing sub-sections for individual aspects. As for the introduction section, I suggest checking and reinforcing a few crucial elements that must be present in the introduction of a paper namely (1) the purpose of the study; (2) the relevance and significance of the study; (3) how it seeks to answer the set research question; and (4) the main contributions of the article. | The first section of introduction (Transformation of social-ecological systems through synergies) was restructured into four paragraphs representing (1) to (4) and explanations were added in each paragraph to emphasize critical elements proposed by the reviewer #1. |
| In my opinion, some of the content included in the introduction could be channeled into a section 2 called for example literature review or theoretical framework as it is more specific and focused on more detail, compared to the initial preamble. Particularly in that the theoretical lens through which the topic is to be analyzed is specified.  Therefore, I would advise the authors to restructure pages 3-5 by constructing an introduction with the purpose and research question and then a second, more theoretical section explaining in detail the theoretical lens chosen for the article and consequently renumber the sections to follow. | The Introduction was divided into “Transformation of social-ecological systems through synergies,” “Research questions,” and “Theoretical framework” sections.  The Theoretical framework section was further divided into two subsections, “Network analysis for leverage point identification” and “Synergies to promote transformation of complex systems,” to highlight important concepts. |
| - Materials and Methods:  I suggest the authors place the Table within the text closely so that the text can be read on page 6.  I also suggest the authors avoid excessive fragmentation in the second session however by providing a subdivision only for data collection and implemented data analysis. | Table 1 was moved to the end of the first paragraph of methods.  We reorganized Materials and Methods into two sections covering initial data processing (Classification of autonomous innovations) and analytical procedures （Analyses of processes and factors of synergy creation）. |
| - Results and Discussion:  Also, for this section, I would avoid excessive fragmentation of the text and provide for splitting the Results and Discussion sections. I would advise the authors to enrich a bit their results, which appear a bit too descriptive and fail to represent more qualitatively the results that emerged also considering the theory and potential implications. This part could go to enrich an individual section based on the Discussion of Emerging Findings. | We made “Results,” “Discussion of Emerging Findings,” and “Conclusion, Limitations, and Future Research Agenda” sections. The theory and potential implications were covered in the “Conclusion, Limitations, and Future Research Agenda” section.  We moved fig 3 to the “Discussion of Emerging Findings” section and renumbered it as fig 5 to summarize the emerging findings on the processes and mechanisms of synergy creation in autonomous innovations. |
| Enhancing what may be the implications for society and policymakers. I would enrich this concluding part by providing a section on “Conclusion, Limitations and Future Research Agenda” precisely to highlight the limitations of the study and how these can be overcome by future research. | Implications for societal actors and policymakers/development agencies were summarized at the end of the “Conclusion and guiding principles” subsection in the “Conclusion, Limitations, and Future Research Agenda” section.  We added discussions on the limitations of this study and current ideas to overcome these challenges in the “Limitations and Future Research Agenda” subsection. |
| I hope you will find these comments and suggestions helpful in further developing this study and I wish you all the best for developing your work! | We are strongly stimulated and motivated by your comments. We hope that we have made significant improvement in the clarity and logical consistency of the paper. |
|  |  |
| **Reviewer #2** | **Response** |
| The manuscript titled “Mechanisms of synergy creation for social-ecological transformation” explores an innovative and relevant topic by analyzing mechanisms for creating synergies in socio-ecological transformations through leverage points. This approach is particularly effective for studying complex adaptive systems. It employs a well-justified and clearly explained methodology, using causal network diagrams to analyze processes of autonomous innovation. This technique allows for precise mapping of key factors and interactions. Moreover, the article provides six practical guiding principles for fostering socio-ecological system transformations, designed for implementation by practitioners, interdisciplinary scientists, and local leaders. Its robust findings, derived from a comparison between “synergistic” and “non-synergistic” cases, highlight critical factors for synergy creation, offering valuable insights for both theory and practice. The evidence base is strong, drawing on 17 cases of autonomous innovations across diverse geographical and cultural contexts, enhancing the generalizability of the conclusions. | We are delighted to receive positive and stimulating comments to improve our paper from the viewpoint of complex adaptive systems. Thank you very much for your feedback. |
| The introduction, while detailed, could benefit from greater clarity and conciseness, focusing on synthesizing key concepts such as the role of synergies and leverage points. | The Introduction was entirely reorganized to emphasize the key concepts in the theoretical framework subsection to achieve greater clarity.  Please also refer to the response to reviewer #1. |
| Some results, including the specific roles of different leverage points (LP(in), LP(out), LP(all)), would be more comprehensible with concrete examples from the analyzed cases. | Examples were given in the methods at the part of explaining LP types using Fig 1A and 1B. Please refer to the paragraph from line 251 to 268. |
| Although the article acknowledges the complexity of socio-ecological systems, it lacks a thorough discussion of methodological limitations, such as potential subjectivity in narrative analysis. | We discussed potential subjectivity referring to our previous papers in the first paragraph of the “Analyses of processes and factors of synergy creation” subsection in “Materials and Methods.”.  The difficulties in avoiding arbitrariness and further research challenges were also discussed in detail in the “Limitations and Future Research Agenda” subsection at the end of the paper in reference to the transdisciplinary approach. |
| While the article references a broad range of literature, a tighter integration of its findings with existing research on complex systems and socio-ecological transformations would be beneficial. | We added a paragraph at the beginning of the “Network analysis for leverage point identification” subsection in “Theoretical framework” to explain the relevance of this study in the complex adaptive system research.  We also discussed the importance of this study in the context of complex systems in the first paragraph of the“Discussion of Emerging Findings” section.  Eight new references were added in these paragraphs. |
| The figures, particularly Figures 2 and 3, could be visually enhanced with clearer labels and more detailed explanations. | All network diagrams, including Figures 2, were revised to high-quality TIFF files to produce better visibility. Node labels were improved to make it clear. All figures were checked using PACE to meet PLOS requirements. Explanations were added in the text related to figure 2 at the second paragraph of the “Results” section.  We moved fig 3 to the “Discussion of Emerging Findings” section and renumbered it as fig 5. It was also checked using PACE. Explanations were added to summarize the emerging findings on the processes and mechanisms of synergy creation. |
| Lastly, certain technical terms, such as “synergy-related value creation” could be better explained to improve accessibility for a non-specialist audience. | We carefully checked inadequate jargon and rewrote it for non-specialist audiences. For example, “synergy-related value creation” was rephrased to “creation of values leading to synergies.” |
| The article makes a significant contribution to the field of socio-ecological transformations and autonomous innovations. Enhancements in clarity, methodological transparency, and integration with existing literature would further strengthen its scientific and practical impact. | Thank you very much for your encouragement. We tried our best to strengthen the scientific and practical impacts of our paper. |
