# Supplementary material for: Mechanisms of synergy creation for social-ecological transformation: Leverage point analysis of the emergence of autonomous innovations
Source: PLoS One. 2025 May 14;20(5):e0323451. doi: 10.1371/journal.pone.0323451 (PMC12077674; doi:10.1371/journal.pone.0323451)
Supplement: Attachment — Submitted filename: Response_to_Reviewers_auresp_2.docx [file pone.0323451.s007.docx]

Ref. No.: PONE-D-24-53688R1

Title: Mechanisms of synergy creation for social-ecological transformation: Leverage point analysis of the emergence of autonomous innovations

PLOS ONE

Dear Editor,

Thank you for the opportunity to revise and resubmit the second revision of our manuscript to PLOS ONE. We are grateful to the two reviewers whose suggestions have allowed us to improve the manuscript to be publishable.

We made further revisions according to the comments. Please find below a point-by-point response to the referee's comments.

Reviewer #1: Dear Author(s),

Thank you for allowing me to review your work again. I believe that the revision process you

implemented has improved the overall quality of the manuscript and its readability, greatly

increasing its potential. However, in my opinion, there are still some aspects that need to be

resolved. I hope that the following recommendations will improve and strengthen the

manuscript:

Thank you very much for your positive and constructive comments on the manuscript. We try our best to improve the quality of the manuscript as indicated below following your valuable comments.

• Introduction: I consider that the introductory section of the paper has been greatly improved,

making some key aspects of the manuscript much more identifiable, such as the purpose of the

article, which is now clearly identifiable. I recommend that the author(s) include some

concrete examples of social ecological systems (SESs) to make the topic more accessible to

the reader and reduce its abstractness.

We briefly introduced examples of SESs (UNESCO Biosphere Reserves and Social-ecological Production Landscapes and Seascapes) in the introduction with three new references.

I would suggest giving some additional references for

what is meant by ‘autonomous innovation’, even if they are then detailed further in the

‘Materials and Methods’ section, to give the reader an overview right from the start of the

paper.

We added a sentence to explain the concept of autonomous innovation, referring to two papers.

I suggest the author(s) remove the subsection ‘Research Question’ and make the text all

in a single section with the research questions inside, then move on to section 2, ‘Theoretical

Framework’.

We removed the subsection ‘Research Question.’

• Theoretical Framework: I appreciated the restructuring of the document, which made it

much more readable and understandable, explaining the theoretical key of leverage points

(LP) and the reference context that considers uncertainty and the achievement of the SDGs

through potential synergies.

Thank you very much for your kind comments.

.

• Materials and Methods/Findings: I consider this section much improved in terms of structure

and readability.

We are pleased to know the improved readability of these sections.

• Discussion: I would recommend that the author(s) call this section simply ‘Discussion’.

The title of this section was changed to ‘Discussion’.

I also recommend that the author(s) link the discussion of the results more closely to the

theoretical lens adopted in the paper, specified in the ‘Theoretical Framework’, making it clear

how the work fits in with existing theory, and citing some of the key references discussed in

section 2.

We added or modified five sentences in ‘Discussion’ to link our arguments with the theoretical lens proposed in ‘Theoretical Framework.’ Five key references were cited to show the relevance of our arguments with existing theory.

• Conclusion, Limitations and Future Research Agenda: I consider the concluding section to

be much improved.

Thank you very much. We also added another paragraph in ‘Limitation and Future Research Agenda’ to address future research opportunities on synergy creation mechanisms across much broader societal sectors such as education, health, and social welfare. Two new references were added with this regard.

I congratulate the author(s) on the extensive improvements made and hope that these

additional comments will increase the potential and quality of the study. Good luck with your

research!

We would like to express our deep gratitude for receiving your valuable comments throughout the review process. It was an important learning opportunity for us to improve our research.

Reviewer #2: Dear Authors,

Thanks for your revisions according to the comments. I am thankful for the effort you have

put into enhancing the manuscript's clarity, structure, and overall quality. The reorganization

of the Introduction, along with the addition of examples and supplementary references, truly

helps to strengthen the theoretical foundation and make the paper easier to read for a broader

readership.

Your diligent attempt to provide for potential subjectivity and to mention the challenges of

avoiding arbitrariness adds depth along with transparency to your methodological approach.

The changes to the figures, including the enhancement of

Overall, I am pleased with the revisions and believe that the manuscript is now publishable. I

commend you for attempting to enhance the paper and address the reviewers' comments

thoroughly.

I have no comments at this time and recommend the manuscript for acceptance.

We are pleased to receive your in-depth understanding of the efforts we have made to improve the manuscript according to your comments. It was a great opportunity for us to deepen our understanding of the complex adaptive systems and transparency in narrative analyses. We are really indebted to your valuable thoughts and comments.
